# A handheld platform for target protein detection and quantification using disposable nanopore strips

**DOI:** 10.1038/s41598-018-33086-7

**Published:** 2018-10-04

**Authors:** Trevor J. Morin, William L. McKenna, Tyler D. Shropshire, Dustin A. Wride, Joshua D. Deschamps, Xu Liu, Reto Stamm, Hongyun Wang, William B. Dunbar

**Affiliations:** 1 Two Pore Guys Inc., Santa Cruz, CA USA; 2 Baskin School of Engineering, University of California, Santa Cruz, CA USA

## Abstract

Accessible point-of-care technologies that can provide immunoassay and molecular modalities could dramatically enhance diagnostics, particularly for infectious disease control in low-resource settings. Solid-state nanopores are simple and durable sensors with low-energy instrumentation requirements. While nanopore sensors have demonstrated efficacy for nucleic acid targets, selective detection and quantification of target proteins from sample background has not been demonstrated. We present a simple approach for electronic detection and quantification of target proteins that combines novel biomolecular engineering methods, a portable reader device and disposable nanopore test strips. The target of interest can be varied by swapping the binding domain on our engineered detection reagent, which eficiently binds in the bulk-phase to the target and subsequently generates a unique signature when passing through the pore. We show modularity of the detection reagent for two HIV antibodies, TNF*α* and tetanus toxin as targets. A saliva swab-to-result is demonstrated for clinically relevant HIV antibody levels (0.4–20 mg/liter) in under 60 seconds. While other strip-like assays are qualitative, the presented method is quantitative and sets the stage for simultaneous immunoassay and molecular diagnostic functionality within a single portable platform.

## Introduction

To address disease control needs in resource-limited settings, the WHO Sexually Transmitted Diseases Diagnostics Initiative created the ASSURED criteria^1^: Affordable, Sensitive, Specific, User-friendly, Rapid and robust, Equipment-free or “untethered”^2^, and Deliverable to end-users. Although the study focused on molecular diagnosis of curable STDs, the same criteria is relevant for any prospective point-of-care (PoC) technology. To maximize impact, a technology positioned to achieve the ASSURED criteria should also permit multiplexing and support molecular and immunoassays.

We developed a nanopore-based technology that could be a sensing platform upon which the ASSURED criteria can be realized, while supporting a multiplexed and multi-modal menu. Here we demonstrate a novel method for selective nanopore detection of protein targets from sample background, including saliva, serum and plasma^3^. The target binding domains are attached to DNA “scaffolds” using bioengineering methods that can accommodate a large menu of domain types, including peptides, aptamers, affibodies and antibodies. Thus, binding domains optimized for sensitivity and specificity within any other assay format can be incorporated into our methodology. Prior nanopore studies on protein detection were performed in ideal buffer conditions using unmodified solid-state nanopores^4–6^ or surface modified pores to enable target specificity^7,8^. These approaches, however, suffered from poor yield due to a high nanopore failure rate (90% loss in^8^). Methods similar to ours have been employed for selective target detection^9–12^, with only^10^ addressing quantification but through the use of fluorescent polarization data and not the nanopore data.

In traditional diagnostics, the gold-standard laboratory technology is the enzyme-linked immunosorbent assay (ELISA). However, resource-limited settings lack the infrastructure to support standard ELISA protocols, which take hours. Several studies have sought to compress and integrate the ELISA protocol to achieve a Point-of-Care technology. By exchanging quantification for qualitative results, minimalist protocols can substantially reduce cost and permit multiplexing with low sample input in 15–20 min^13,14^, or produce a visual yes/no test with high sensitivity^15^. Recapitulating quantification, however, requires more instrumentation and/or human involvement due to the requirement for multiple wash steps^16^. Single molecule array technologies such as Simoa by Quanterix and other digital ELISA formats may outperform standard ELISA, but remain expensive, lab-confined and time intensive^17–19^.

When qualitative results are acceptable and targets are in sufficient supply (>50 *μ*g/liter), lateral flow immunoassays (LFAs) are an inexpensive sample-to-answer test that require no additional equipment and produce results in 10–15 min^20,21^. While LFAs are not amenable to multiplexing beyond a few test lines, inexpensive paper-based immunoassays are capable of multiplexing^22^. Since LFAs are often not sufficiently sensitive or specific, they do not “… meet the ideal ASSURED product profile”^1^. Instrument-fortified LFAs have demonstrated quantification and enhanced sensitivity and specificity. Commercial examples (Lepu Leccurate) and comparable technologies (Alere Triage system, Abbott i-STAT, Abaxis and Gyrolab centrifugal CD systems) were recently reviewed in the context of immunoassays for cardiovascular diseases (CVDs), the leading causes of death worldwide^23^, but these technologies are lab-confined.

Next generation PoC technologies will likely be enabled by novel microfluidics and nanotechnologies. Published results have demonstrated unprecedented multiplexing from reduced sample volumes; however, the instrumentation required is often bulky, complex and expensive. A barcoding chip achieved a multiplexed panel (8–12) of plasma proteins from a finger prick of whole blood in 10 min, but an expensive microarray scanner was needed for quantification^24^. A plasmon resonance device achieved 8-log range and 0.4 ng/liter limit of detection, but required a multi-light spectral tool or cartridge-mounted movable stage^25^. A comprehensive review on microfluidic immunoassay chips that spanned optical, electrochemical and mechanical methods found common challenges^26^. Functionalized surface-based sensors that maintain target specificity in the presence of sample matrices was cited as a key challenge. The need for channel flow for mass transport to promote surface binding, common to many protocols, often translates to long (~1 hour) incubation periods^25,27^, though shorter periods have been achieved (15 min^28^). Another challenge was a common need for wash steps that, like ELISA, translates to more human interventions or higher instrumentation costs to automate the micofluidics. Closest to nanopores are electrochemical readouts from functionalized nanowires^29^ and nanoribbons^30^. While these sensors have demonstrated high sensitivity (low *μ*g/liter), it is unclear if they can be fabricated at sufficiently high yield to keep device costs acceptably low.

We achieved target specificity by engineering a molecule that has three functions: (1) to capture the target in bulk phase via a binding domain, (2) to shuttle the target through the pore via a charged polymeric scaffold attached to the binding domain, and (3) to generate a unique electrical signature when it passes through the pore with target bound. Specificity and sensitivity are governed by the affinity of the target binding domain, and the detectability and uniqueness of the electrical signature. Since binding between the target and engineered molecules happens by diffusion in the bulk phase chamber, incubation periods sufficient to achieve equilibrium are faster than for surface-binding assays that also rely on diffusion^31^. Including only 30 seconds of incubation, our saliva swab-to-result is demonstrated for 0.4–20 mg/liter HIV antibody in under 60 seconds total. We demonstrate selective detection of two different HIV antibodies (150 kDa), TNF*α* protein (52 kDa), and tetanus toxin protein (TT, 150 kDa). Using these data, we present a novel mathematical framework for target quantification.

## Results

### Test strips for single molecule sensing

**Figure 1:**
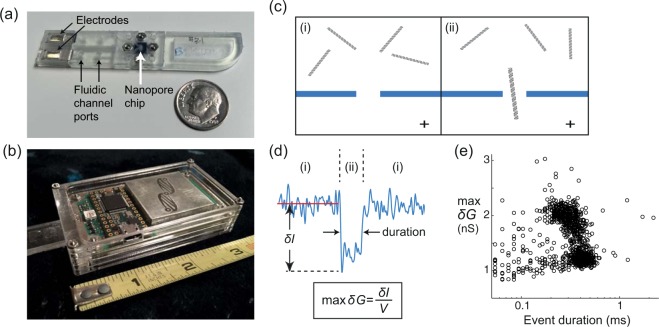
Single-molecule sensing by inserting a disposable strip into a USB-enabled mobile device. (**a**) The 3D printed strip houses the fluidics and nanopore chip (Supplementary Fig. S1). Replaceable Ag/AgCl electrodes connect the fluidic channels with the circuitry in the reader device. Reagents and buffer are loaded into one channel, with buffer only in the other channel. (**b**) The strip is inserted into the handheld device that houses voltage-clamp circuitry, which supplies a voltage across the pore and measures ionic current through the pore. Data transfer is through a USB port for event detection and analysis on a PC or laptop (Supplementary Figs S3 and S4). (**c**) Schematic of diffusing DNA above the nanopore on the reagent-added side, during (i) an open nanopore period, and (ii) passage of a single DNA through the pore into the voltage-positive side. (**d**) A representative electrical current event caused by a single dsDNA passing through a nanopore, annotating (i) the open pore periods and (ii) the passage duration. Events are quantified by the passage duration and the maximum conductance depth (max *δG* = *δI*/*V*, *V* = 0.1 V). (**e**) An all-event scatter plot of max *δG* versus duration displays 1049 events of 5.6 kb dsDNA recorded in 5 minutes with a 22 nm diameter nanopore.

The reader device and test strips used in this study (Fig. 1a,b) were designed to support laboratory-based development protocols, while future iterations are being integrated to support sample-to-answer applications. The buffer is pipetted into one channel and reagents and buffer are pipetted into the other channel and the strip is inserted into the reader, which is connected to a laptop by a USB cable. Custom software then drives the reader to supply a voltage and record the ionic current through the pore. The voltage captures the molecule and drives it through the pore into the opposing chamber when a single charged molecule such as DNA diffuses sufficiently close to the pore (Fig. 1c). The passage “event” is detected by the reader circuitry as a temporary shift in the ionic current, which is quantified by the passage duration and maximum conductance depth, max *δG* (Fig. 1d). A population of single molecule events is recorded over time (Fig. 1e), earning our instrument the moniker “MOM” for Molecule Occlusion Meter (Supplementary Table S1). Our platform also performs comparably to the Molecular Devices 700B amplifier (Supplementary Table S2, Supplementary Figs S5 and S6), the most commonly used brand in nanopore research. We fabricated our own nanopore chips and verified that they perform comparably to state-of-the-art low-noise solid state nanopore devices^32^ (Supplementary Fig. 2, Supplementary Methods).

### Target detection by single molecule event sorting

Selective detection of a target protein is achieved by sorting every recorded event into one of two categories: target positive or target negative. This is in contrast to the event-pattern mining used in^9–12^, where a non-trivial fraction of events (up to 85% in^9^) are trimmed, undermining quantification. These methods also require glass nanopipettes that are difficult to mass manufacture and ruggedize. By contrast, the structurally robust nanopores utilized in this study cover a wide range of diameters, spanning 18–46 nm for the 20 pores used for HIV Ab targets, and are amenable to manufacturing using high volume fabrication tools.

Our method is achieved by first creating a detection reagent that generically combines a charged polymer scaffold molecule with a bi-functional fusion molecule. The fusion molecule has one domain for attachment to the scaffold, and a second domain for binding the target. The charged polymer, commonly single or double-stranded DNA (dsDNA), is chosen for facile nanopore capture and detection. We combined a 1074 bp dsDNA scaffold with a bisPNA-peptide as the bi-functional fusion molecule (Methods). The bisPNA was designed to bind centrally to the dsDNA with high stability^33^. The peptide sequence comprises an epitope within the third variable loop (V3) of the HIV-1 viral envelope glycoprotein^34^. People infected with genotype clade E HIV are known to generate primarily non-neutralizing antibodies to the V3 loop sequence, making it a good epitope for diagnosis^35^.

Single-molecule sorting is accomplished when the target-bound detection molecule (DNA/PNA-peptide/antibody) creates a unique signature compared to other molecules present in sample passing through the nanopore. Other molecules include unbound detection reagent (DNA/PNA-peptide), any free sub-components of the detection reagent, free antibody target, molecules from diluted sample matrices when present, or any combinations thereof. Reagents comprising the detection molecule and known concentrations of target, including human monoclonal antibody 447-52D in a first set of experiments, and recombinant monoclonal antibody HGD65 in a second set of experiments, were tested as model targets.

**Figure 2:**
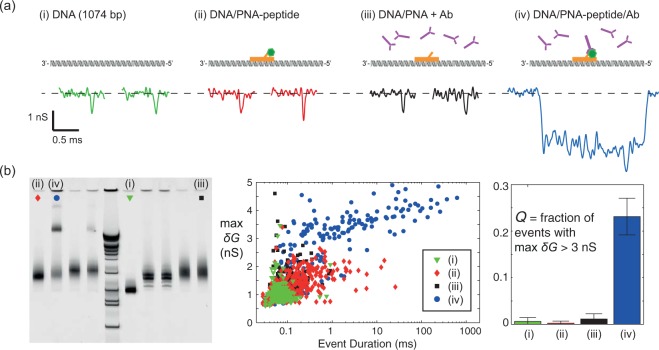
Selective detection of HIV antibody 447-52D using a single nanopore and an engineered detection reagent. (**a**) Contrasting molecular cartoons and recorded nanopore event signatures for (i) DNA alone, (ii) DNA/PNA-peptide detection reagent, (iii) DNA/PNA (*sans* peptide) with antibody and (iv) detection reagent bound by antibody. Each reagent (i–iv) was measured sequentially on the same 27 nm diameter pore. In between each reagent, the nanopore was flushed with clean buffer for 5 minutes. The number of events *N* and recording periods were: (i) *N* = 332 in 16 min, (ii) *N* = 434 in 13 min, (iii) *N* = 353 in 18 min, (iv) *N* = 441 in 19 min. (**b**) EMSA and nanopore event plots display consistent trends for (i–iv). Unlabelled lanes were not analyzed on the nanopore in this study. The gel upper band that is indicative of full complex (DNA/PNA-peptide/antibody) matches the pronounced increase in nanopore events with larger max *δ*G values. In particular, while less than 2% of events for (i–iii) are above max *δ*G = 3 nS, the fraction increases to 23% for full complex (iv), a trend used for positive detection with 99% confidence as described in the main text. The variable *Q* denotes the fraction of events tagged as target positive, with 99% confidence interval $2.58\sqrt{Q\mathrm{(1}-Q)/N}$.

In each nanopore experiment, a set of reagents are sequentially measured on the same pore, with flushing of buffer between reagent pairs. Reagent sets here include positive controls, which include detection reagent and the target at varying concentrations, and negative controls, which exclude the target or some necessary component of the detection reagent. Prior to nanopore experiments, electrophoretic mobility shift assays (EMSAs) verified that full DNA/PNA-peptide/447-52D complex formation proceeded the incubation conditions employed (Fig. 2b, Methods, Supplementary Fig. S8). Nanopore experiments with positive control reagents revealed a unique population of events with larger max *δG* attributable to full DNA/PNA-peptide/447-52D complex, consistent with a unique EMSA band (Fig. 2a,b, reagent iv). Negative controls run on the same nanopore, including detection reagent in the absence of 447-52D and DNA/PNA without peptide but with 447-52D, generate event populations similar to DNA alone (Fig. 2a,b, reagents i–iii). These negative controls do not recapitulate the pronounced fraction of larger max *δG* events that are observed with positive control reagent sets.

We implemented our previously developed method to calculate target positive vs. negative^36^ (Supplementary Methods). The first step is to create an event-based criterion with which to sort events as target positive or target negative. Based on the data in Fig. 2, events were target positive if max *δ*G > 3 nS. Next, each event has random variable *Z* defined to be 1 when the event is tagged, and 0 if not tagged. While *Z* has a Bernoulli distribution, the fraction of *N* events meeting the criteria $Q={\sum }_{j=1}^{N}\,{Z}_{j}/N$ has a Binomial distribution with 99% confidence interval ${Q}_{\ast }=2.58\sqrt{Q\mathrm{(1}-Q)/N}$. We refer to *Q* as the tagged-fraction of events. Applying the criteria to reagents (i-iii) produces *Q* ± Q_*_ values (after converting to percentage) of 0.6 ± 1.1%, 0.2 ± 0.6% and 1.1 ± 1.5%, respectively, while reagent (iv) measured 23.1 ± 5.2%. By assigning a false positive threshold on the fractional scale as *Q*_f.p._ = 0.02 (2%), the negative control reagents produce negative results while the positive control with both detection reagent and 447-52D present produced a positive result with 99% confidence, since 0.231−0.052 > 0.02.

For the data in Fig. 2 and all subsequent nanopore data sets, the test for presence of a target protein is positive with 99% confidence if *Q*−Q_*_ > *Q*_f.p._; otherwise, the result is negative. The negative controls for each pore or across of set of pores are used to identify the max *δ*G threshold such that *Q* ≤ *Q*_f.p._, keeping *Q*_f.p._ = 0.02. All reagents with detection reagent and target present are then tested using *Q* − Q_*_ > *Q*_f.p._ and the same max *δ*G threshold. For each 99% positive result, we report the sensing time-to-positive (TTP), defined as the first time at which *Q* − Q_*_ > *Q*_f.p._ is true and remains true. The TTP for reagent (iv) in Fig. 2 is 1.35 min. Of the seven total reagent sets run on this pore, only the two with full complex present (i.e., the two positive controls) produced positive results (pore P1, Supplementary Table S3, Supplementary Fig. S7).

Comparable reagent sets to those shown in Fig. 2 were tested on two other nanopores (P2-P3, Supplementary Table S3, Supplementary Fig. S7). Nanopore and EMSA assays determined that the peptide located at the bisPNA terminus produced superior target binding performance compared to an interior location (Supplementary Methods). The reagents were also tested using traditional benchtop nanopore setups (P1-P3) and the MOM and test strip setup (P4-P6, Supplementary Table S4) to compare performance. In all cases, the presence of DNA/PNA-peptide/447-52D registered positive with 99% confidence as a distinct population above negative control reagents, even in the presence of diluted serum above the nanopore (1:1000, 1:333, 1:250; Supplementary Fig. S9). One reagent set with a longer scaffold (3250 bp) was also tested for target binding efficiency, which reduced the incubation period to less than 10 seconds and produced a 99% positive with TTP of only 7 seconds (P6, Supplementary Fig. S10).

EMSA verified full complex formation of the second HIV Ab target HGD65, followed by seven separate nanopore experiments (pores P7-P13, Supplementary Tables S5 and S12, Supplementary Figs S11–14). Two shorter scaffolds (217 bp, 108 bp) were also tested in four nanopore experiments (pores 14–17, Supplementary Table S19). All eleven nanopore experiments (P7-P17) resulted in 99% positive target detection for all HGD65 concentrations tested, spanning two logs (0.1–20 nM) above the pore with TTP values from seconds to minutes (Supplementary Tables). Positive detection and efficient TTP were also observed for three nanopore experiments with 1:250 dilution of serum (P18-P20, Supplementary Table S26) and six nanopore experiments with 1:100 dilution of saliva (P21-P26, Supplementary Tables S30 and S37). Two of the experiments (P25, P26) utilized 30 second incubations (10% saliva), achieving sample-to-positive in less than 60 seconds total for 0.266-13.3 nM HGD65 (Supplementary Fig. S26). Concentrations reported here are after a dilution step (pre-dilution concentrations are reported in the referenced Supplementary Tables).

### Target quantification from sorted single molecules

We measured a known set of HGD65 concentrations while keeping scaffold/fusion concentration constant. A set of reagents were sequentially measured, with adequate flushing of buffer between each pair of reagents, on a single nanopore to generate data for modeling. A model was then fit to the data, and model-based predictions were tested against the known target concentrations. The variable *X* is defined as the target-to-scaffold/fusion concentration ratio, and the tagged fraction of events is modeled as *Q* vs. *X* for sub-saturating and saturating conditions. We derived a phenomenological model (Supplementary Methods) for the fraction of tagged events *F*_tag_(*X*):

1 $$\begin{array}{c}{F}_{{\rm{tag}}}(X)=\frac{\alpha {q}_{1}{F}_{{\rm{bound}}}(X)}{1+({q}_{1}-\mathrm{1)}{F}_{{\rm{bound}}}(X)},\\ \,\,\,\,\,\,{\rm{with}}\,{F}_{{\rm{bound}}}(X)={q}_{2}+1+X-\sqrt{{({q}_{2}+1+X)}^{2}-4X}.\end{array}$$

**Figure 3:**
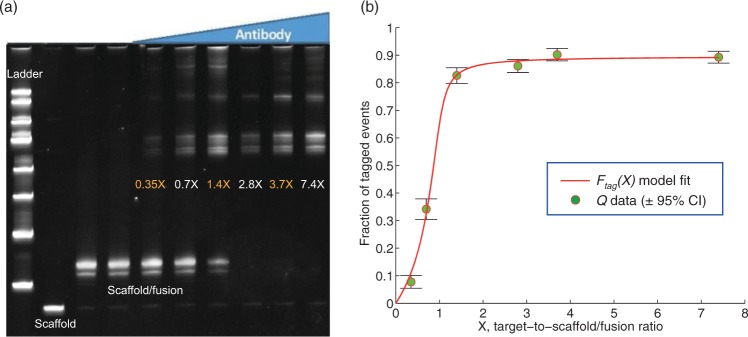
Measuring and modeling the fraction of tagged events as a function of HGD65 target-to-scaffold/fusion ratio. (**a**) EMSA assay showed increasing fraction of target-bound scaffold/fusion molecules as HGD65 antibody concentration increases, using a 217 bp/PNA-peptide detection reagent. (**b**) The data are the tagged fraction of recorded events with the 95% confidence interval $(Q\pm 1.96\sqrt{Q\mathrm{(1}-Q)/N})$ at six different target-to-scaffold/fusion ratios *X*. The fitted three-parameter model *F*_tag_(*X*) is defined in Eq. (1). The reagent for each ratio *X* was measured sequentially on the same pore (P15, Supplementary Table S19), with 5 minutes of event-free buffer only recording in between reagents. The number of events *N* and ordered recording periods for each *X* were: (i) *X* = 0, *N* = 475 in 7 min, (ii) *X* = 2.8, *N* = 819 in 17 min, (iii) *X* = 1.4, *N* = 685 in 14 min, (iv) *X* = 0.7, *N* = 615 in 13 min, (v) *X* = 0.35, *N* = 516 in 10 min, (vi) *X* = 3.7, *N* = 663 in 16 min, (vii) *X* = 7.4, *N* = 783 in 14 min. Model fitting error was 2.2% using a normalized root-mean-square (Supplementary Table S20). The *Q* data for *X* = (0.35, 0.7, 1.4) are sufficiently below saturation to permit concentration estimation, as described in the main text. The results are estimated (0.53 ± 0.07, 1.4 ± 0.05, 2.8 ± 0.3) nM compared to knowns (0.7, 1.4, 2.8) nM (Supplementary Table S21). Thus, a single pore nominally has a 2-log detection range and 1-log quantitative range.

There are three parameters: *α* is the probability of tagging an event caused by a scaffold/fusion/target molecule (i.e., the true positive probability); *q*_1_ is the ratio of capture rate constants between the scaffold/fusion/target and the unbound scaffold/fusion; and *q*_2_ is the ratio of *K*_*d*_ to known total scaffold/fusion concentration. The three parameters (*α*, *q*_1_, *q*_2_) are fit to the data using direct simplex search optimization. The *Q* vs. *X* data and model fit for pore P15 are shown in Fig. 3b as a representative example.

A simplified two parameter model ignores the difference in capture rate constants between target-bound and unbound scaffold/fusion molecules by setting *q*_1_ = 1, resulting in *F*_tag_(*X*) = *αF*_bound_(*X*). Using a normalized root-mean-square (NRMS) error to quantify model fitting performance, the three-parameter model (NRMS = 2.2%) outperformed the two-parameter model (NMRS = 14%) for P15 in Fig. 3. In fact, the three-parameter model outperformed the two-parameter model aross all experiments with quantitative data, including ten buffer-only control experiments (Supplementary Tables S6, S13, S20) and six experiments with saliva background (Supplementary Tables S31, S38). The three- and two-parameter models were also adapted to fit to the capture rate of tagged events (Supplementary Methods), which generally did not perform as well as modeling the *Q* data. Of related published research^9–12^, only^10^ claimed quantitative results; however, only fluorescent polarization data was modeled by fitting *K*_*d*_ with the two parameter model *F*_bound_(*X*).

For the purpose of estimating *K*_*d*_ between the target and binding domain, the two parameter model was consistent with ELISA (Supplementary Methods). Specifically, *K*_*d*_ = 0.86 ± 0.58 nM from 5 experiments with 1074 bp scaffold and *K*_*d*_ = 0.24 ± 0.25 nM from 8 experiments with 217 bp scaffold, while duplicate ELISAs run on a plates coated with PNA-peptide and peptide alone produced *K*_*d*_ = 0.4 ± 0.2 nM and *K*_*d*_ = 2.2 ± 1.1 nM, respectively (Methods).

Concentration estimation is based on inversion of model Eq. (1) by replacing *F*_tag_ with *Q* and solving for *X*, given the measured data *Q* and model parameters values (*α*, *q*_1_, *q*_2_). Performance of this method and estimation using the two parameter model are assessed and compared in detail in the Supplementary Methods. Interestingly, single-pore estimation performance is not uniformly distributed within the predictive range, but appears optimal for 0.5 < *X* < 5 (Supplementary Fig. S23). Model-based estimation using the tagged event rate was also examined (Supplementary Fig. S22).

### Demonstrating reagent modularity with TNF*α* and tetanus toxin

**Figure 4:**
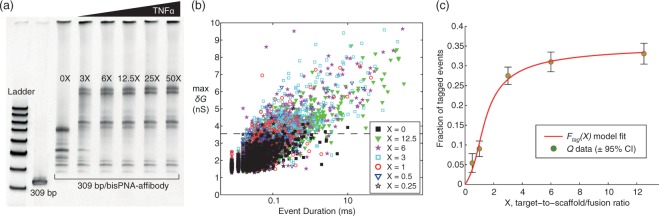
Measuring and modeling the fraction of tagged events as a function of TNF*α*-to-scaffold/fusion ratio, *X*. (**a**) EMSA assay shows increasing fraction of target-bound scaffold/fusion molecules as TNF*α* protein concentration increases, using a 309 bp/PNA-affibody detection reagent (Methods, Supplementary Fig. S27). (**b**) All-event scatter plot of max *δ*G versus duration for reagents with varying *X*. Each reagent was measured sequentially on the same 25–30 nm diameter pore (P29, Supplementary Table S52), with buffer only flushing and recording between reagent pairs. The number of events and recording periods for each reagent are: (*X* = 0) *N* = 518 in 10 min; (*X* = 12.5) *N* = 1222 in 10 min; (*X* = 6) *N* = 1336 in 10 min; (*X* = 3) *N* = 1565 in 10 min; (*X* = 1) *N* = 819 in 10 min; (*X* = 0.5) *N* = 350 in 4 min; and (*X* = 0.25) *N* = 690 in 10 min. Using the *X* = 0 reagent to establish the tagging criteria max *δG* > 3.56 nS (dashed line), all *X* > 0 values were positive with 99% confidence above a false positive of 2%, with the exception of *X* = 0.25 which was not positive. The time to positive (TTP) values: *X* = 12.5 in 6 sec; *X* = 6 in 5 sec; *X* = 3 in 10 sec; *X* = 1 in 41 sec; and *X* = 0.5 in 2.3 min. (**c**) The positively detected data in (**b**) were subsequently modeled using the three-parameter model *F*_tag_(*X*), resulting in parameter values (*α*, *q*_1_, *q*_2_) = (0.35, 0.17, 0.11) and NRMS fitting error of 3.8% (Supplementary Table S53).

The modularity of our method is showcased by applying it to two other targets, TNF*α* and tetanus toxin (TT). First, a 309 bp/bisPNA-affibody construct was used as a detection reagent for TNF*α* (Methods). A sandwich ELISA tested the binding affinity between the affibody and TNF*α* both without and with the 1 M LiCl nanopore recording buffer present, resulting in a conserved *K*_*d*_ ≈ 0.2 nM. EMSA verified full DNA/PNA-affibody/TNF*α* complex formation (Fig. 4a). We followed this with triplicate nanopore experiments (P29 in Fig. 4b,c; P30 and P31 in Supplementary Table S52, Supplementary Fig. S28).

Results with HGD65 antibody target show that saturation approaches 100% bound detection reagent (Fig. 3), but both EMSA and nanopore results with TNF*α*TNF*α* in Fig. 4 suggest that a conserved fraction of detection reagent are not target bound. Further optimization in detection reagent construction will address this. Across three nanopore experiments, five TNF*α* concentrations within the 1.5–37.5 nM range (78–1950 *μ*g/liter) resulted in 99% positive detection with TTP values from 4 seconds to 5 minutes, while 0.75 nM was not positively detected. The model fit produced *K*_*d*_ ≈ 0.2 nM consistent with the ELISA results. Combined target estimates of (1.7 ± 0.02, 2.9 ± 0.1, 9.4 ± 0.9) for known concentrations (1.5, 3, 9) nM resulted in a mean coefficient of variation of 9% and a mean percent error of 7% (Supplementary Tables S53–S55, Supplementary Fig. S29). TNF*α*, a cell signaling protein involved in systemic inflammation, is considered elevated above 2 ng/liter^37^, 4-log lower than demonstrated here. A post incubation scaffold/fusion/target concentration step upstream of nanopore sensing is under development to reach the needed detection sensitivity.

For the final protein target, the detection reagent was engineered to incorporate a full-length antibody as the binding domain for tetanus toxin (TT) (Methods). The scaffold-to-antibody coupling method can be applied to any available antibody or antibody fragment since it utilizes lysines endogenous to the binding domain. A biotin moiety was also added to the scaffold end opposite the binding domain, permitting coupling the detection reagent to streptavidin coated magnetic beads.

We demonstrate: (1) spiking TT into plasma, (2) capturing TT with the detection reagent bound to streptavidin beads, (3) washing out plasma background, and (4) cleaving off the bead with a simple restriction enzyme digest of the DNA scaffold. Post cleavage, the TT bound complexes were verified by EMSA (Supplementary Fig. S30). In replicate experiments, TT was positively detected at 4.5 and 12 nM above the pore in buffer only (P32–P34, Supplementary Table S56, Supplementary Fig. S31) and at 360 nM TT following incubation in 10% and 20% plasma (P35 and P36, Supplementary Table S57, Supplementary Fig. S32). As an alternative to restriction enzyme digestion, we tested release of detection reagent from the bead using UV light (15 sec). The UV flash cleaved a photocleavable linker incorporated between the scaffold and biotin for this purpose (Supplementary Fig. S30).

A key design element for future ASSURED technologies will be exploiting microfluidics for efficient integrated preparation on varying sample input types. For example, a microfluidic “purification” chip demonstrated capture-wash-release of cancer biomarkers from physiological solutions upstream of integrated sensors^30^. The power consumption using the UV light at 15 sec was 21mWh. Future test strip designs could integrate an inexpensive and fast UV-based capture-wash-release protocol upstream of the nanopore sensors.

### Combining estimates to test quantification enhancement

Future test strips will incorporate multiple nanopores, and we anticipate that aggregate quantitative estimates from a multi-pore strip will have superior performance to that of single nanopore estimates. To test this, we averaged the HGD65 antibody target estimates from separate single nanopore experiments at common concentrations, and examined the precision by computing the coefficient of variation (CV) and trueness by computing the percent error (PE). Such combined estimates capture our experimental reproducibility due to the following conditions:

1. Five different people performed experiments, each with a unique device.
2. Each experiment used a new test strip and nanopore chip.
3. The nanopore size range varied across all 15 quantitative experiments.
4. Each experiment was run on a unique date and with a unique set of prepared reagents, with the exception of P14 and P15 (common date and reagents).

**Figure 5:**
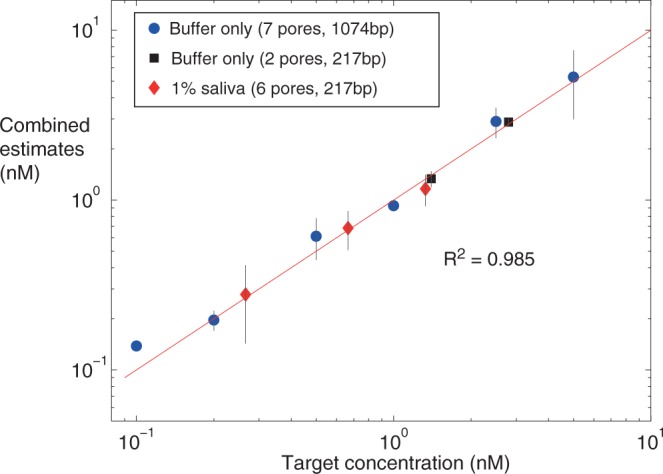
Combining single-nanopore estimates of HGD65 target concentration to gauge accuracy and precision. The combined estimate data plotted at each known target concentration is the average and standard deviation of at least two estimates generated from separate single-nanopore experiments (data in Table 1). The concentrations are above the nanopore after dilution. Single-pore estimates were combined where a common scaffold/fusion reagent was used (7 pores P7-P13, and 2 pores P14-P15) or by the presence of 1% saliva background above the pore (6 pores P21-P26). The *R*^2^ value assesses how closely the average estimates are to the zero error line, with the three-parameter model (*R*^2^ = 0.985, Supplementary Table S45) outperforming the two-parameter model (*R*^2^ = 0.953, Supplementary Table S46 and Fig. S24).

Combined single-pore estimates were generated at eleven different known target concentrations, with the estimates vs. known values reported (Table 1, Fig. 5). At each known target concentration, a combined estimate was generated by taking the average and standard deviation of values from at least two separate single-nanopore experiments. Across all eleven target concentrations, the combined estimates produce a mean CV = 21% and mean |PE| = 11%.

**Table 1:** Combining HGD65 concentration estimates from separate experiments.

Nanopores^*^	Target^†^ Conc. (nM)	Combined^‡^ Estim. (nM)	CV^ь^	PE^ь^
P7, P8	0.1	0.138 ± 0.002	1.09%	38.2%
P7, P8	0.2	0.197 ± 0.027	13.6%	−1.71%
P9, P10, P12, P13	0.5	0.612 ± 0.17	27.6%	22.4%
P9-P13	1	0.927 ± 0.07	7.69%	−7.33%
P10-P13	2.5	2.9 ± 0.59	20.3%	16.1%
P10, P12, P13	5	5.3 ± 2.32	43.7%	5.91%
P14, P15	1.4	1.34 ± 0.143	10.7%	−4.58%
P14, P15	2.8	2.88 ± 0.145	5.02%	2.79%
P21-P23, P25, P26	0.266	0.278 ± 0.135	48.6%	4.6%
P22-P26	0.665	0.684 ± 0.176	25.8%	2.8%
P21, P23, P25, P26	1.33	1.16 ± 0.245	21%	−12.5%
Summary			20.5%	|10.8|%

^*^Pores were grouped together by a common detection reagent (pores P7-P13 with 1074 bp, pores P14-P15 with 217 bp), or by the presence of saliva background (pores P21-P26, 10% saliva at incubation, 1% saliva above the pore after 10X dilution).

^†^Target concentrations are above the nanopore after dilution. The pre-dilution incubation concentrations are reported in Table 2.

^‡^Combined estimates are the mean and standard deviation of the single-pore estimates at each target concentration. The quantitative estimates for the individual pores listed are reported in the Supplementary Tables and plotted in Supplementary Fig. S22.

^ь^Coefficient of variation (CV) is the standard deviation divided by the mean. Percent error (PE) is the ratio of the error (estimate minus known value) to the known value, converted to a percentage. Summary reports the mean CV and the mean of the absolute PE values across all estimates.

We note that the methods and materials presented here support laboratory-based development methods, but not sample-to-answer quantification since a set of controls are required prior to measuring unknowns (this is discussed further below). FDA guidance recommends 15% PE and CV calculated using a minimum of 5 replicate estimates per known concentration^38^. Our approach will likely meet these performance targets, since a multi-pore strip will eliminate conditions 1 and 4 and therefore should perform better than the combined estimates reported here. The one experiment pair that eliminated condition 4 (pores P14-P15) produced mean CV = 8% and mean |PE| = 4% (Supplementary Table S45), well below the 15% requirement.

Both qualitative and quantitative ranges with HGD65 as a model antibody are reported as the target concentrations at incubation to reflect the ranges that would be possible for sample-to-answer protocols without an upstream concentration step during sample preparation (Table 2). The reported detection range is across all single-pore results, while the quantitative range only includes target concentrations for which there were replicate estimates for averaging.

**Table 2:** Summary of HGD65 detection and quantification performance.

Performance Criteria	Buffer Only	Spiked Saliva (10%)	Spiked Serum (10%)
Detection Range^†^	0.39–75 mg/liter	0.4–20 mg/liter	5.25–111 mg/liter
quantification Range^*^	0.75–30 mg/liter	0.4–2 mg/liter	10–21 mg/liter
Precision^*^	mean CV = 16%	mean CV = 32%	n/a
Trueness^*^	mean |PE| = 12%	mean |PE| = 7%	n/a

^†^The qualitative (detection) ranges listed are concentrations at incubation. Nanopore groupings: buffer only, P7-P15 and P27-P28; saliva, P21-P26; serum, P18-P20. 1 nM = 0.15 mg/liter for 150 kDa Ab.

^*^Quantitative ranges listed are concentrations at incubation and correspond to the combined estimates reported in Table 1 and plotted in Fig. 5. Nanopore groupings: buffer only, P7-P15; saliva, P21-P26; serum, P18 alone. Mean coefficient of variation (CV) and mean absolute value of percent error (|PE|) are across the corresponding subgroups (P18: CV, PE not available (n/a) without replicates).

The qualitative range overlaps with the Ab range found in HIV-positive specimens. Specifically, three single-pore experiments (P23, P25, P26) spanned 2.66–133 nM (0.4–20 mg/liter) HGD65 at incubation in 10% saliva, while the equivalent 4–200 mg/liter for neat saliva substantially overlaps with the 0.5–100+ mg/liter IgG range present in HIV positive salivary specimens^39,40^. An ELISA-based approach achieved 10 mg/liter detection from whole blood, in contrast to the 4 mg/liter lower limit for saliva demonstrated here^14^. Additionally, the lowest concentration detected above the pore after dilution was 0.1 nM (15 *μ*g/liter) HGD65 in buffer only (P7, P8) and 0.266 nM (40 *μ*g/liter) in 1% saliva (P21-P23, P25, P26; Fig. 5, Supplementary Methods).

## Discussion

Here we demonstrate 2-log qualitative range for detection of target proteins on a single pore. A multi-pore strip using parallelized chambers would increase the range by using different scaffold/fusion molecules at different concentrations in each chamber. The scaffold lengths of 3250 bp (P6), 1074 bp (P13), 217 bp (P15) and 108 bp (P17) in our study produced capture rate constants of 390, 250, 10 and 0.8 (minnM)^−1^, respectively (Supplementary Tables). Thus a multi-chamber strip could span 3-log in detection reagent concentration, e.g., from 0.1 nM 3250 bp/fusion to 100 nM 108 bp/fusion, while generating approximately equivalent event rates across the pores. Combined with the 2-log range per pore, this would result in a qualitative range of at least 5-log. The qualitative range can also be expanded by varying the voltage and buffer composition.

The predictive range per nanopore can be addressed in part by adding parallel chambers and nanopores and employing a larger detection reagent concentration range, as proposed above. The primary limitation is the need for at least three controls to fit the model parameters prior to measuring the unknown concentration. An automated approach would thus require multiple flushing and measurement stages, adding time and device complexity. Potentially, if the target and detection reagent are sufficiently well characterized, a table-lookup of one or more of the model parameters would expedite this process.

We also developed a novel two-stage method that streamlines the quantitative protocol (Supplementary Methods). Nanopore data is recorded in two sequential stages: (I) known detection reagent with the unknown target amount, followed by (II) the contents of (I) with a known target-to-scaffold/fusion ratio spiked into the chamber. The method is based on the two-parameter model but eliminates the need for model fitting and requires only the single control (II) run after the unknown. As before, the stage (I) data must be below saturation (*X* < 5). As representative examples, the known target concentrations (0.665, 1.33) nM from the saliva sample group (P21-P26) had combined two-stage estimates with mean CV = 27% and mean |PE| = 8% (Supplementary Table S49). This is comparable in performance to the three-parameter model combined-estimates (spiked saliva in Table 2; red diamonds, Fig. 5), which is a promising result considering only a single control is required for quantification using the two-stage method.

The more complicated a laboratory procedure and instrumentation, the more difficult and costlier it is to develop an easy-to-use and inexpensive version for a resource-limited site^2^. Future development will focus on engineering a sample-to-answer nanopore-based solution that can be successfully deployed in low-resource settings^13,41^.

## Methods

### HIV Ab assays

The 1074 bp and other length dsDNA fragments were generated by PCR amplification from linearized plasmid template and purified using the Viogene gel/PCR Isolation System (Viogene cat. #GP1002). All scaffold lengths for the HIV antibody target experiments contain a centrally located sequence (AGGGGAGGAAAA) complementary to a base-pairing strand within bisPNA. The bisPNA molecule consists of two halves separated by a flexible PEG linker, the first half binds to the cognate site using Watson-Crick base pairing and the second half binds via Hoogsteen face pairing^42^. Two terminal lysines are included on each end to enhance complex stability with DNA^33^. A cysteine residue was incorporated into either the flexor region (PNA^0^) or at the Hoogsteen face pairing end (PNA^1^) to provide a chemical handle for covalent coupling of the maleimide-containing peptide to the PNA prior to hybridizing with dsDNA. The two bisPNA designs examined had the following sequence and structure (N-term left, to C-term right):

$$\begin{array}{c}{{\rm{PNA}}}^{0}:\mathrm{KK} \mbox{-} O \mbox{-} \mathrm{TCCCCTCCTTTT} \mbox{-} O \mbox{-} \mathrm{Cys} \mbox{-} O \mbox{-} \mathrm{TTTTJJTJJJJT} \mbox{-} O \mbox{-} \mathrm{KK}\\ {{\rm{PNA}}}^{1}:\mathrm{KK} \mbox{-} O \mbox{-} \mathrm{TCCCCTCCTTTT} \mbox{-} \mathrm{OOO} \mbox{-} \mathrm{TTTTJJTJJJJT} \mbox{-} O \mbox{-} \mathrm{KK} \mbox{-} \mathrm{OO} \mbox{-} \mathrm{Cys}\end{array}$$

where K is the amino acid lysine, O represents a 2-aminoethoxy-2-ethoxy acetic acid linker, and Cys is a cysteine. To form bisPNA-peptide, the PNA is first incubated with TCEP to reduce any intermolecular disulfide bonds, and then reacted with a 10 to 50-fold excess of peptide. The end terminated peptide sequence KSIHIGPGRAFYTT (JPT, Germany) is the highly immunogenic V3 loop of the HIV envelope glycoprotein. The PNA^0^-peptide used a PEG terminated peptide sequence, PEG_5_-peptide, creating the two PNA strands flanking the peptide strand. The PNA^1^-peptide used Ttds-peptide (PNABio), with the peptide at the C-terminal end of the PNA. The PNA-peptide conjugate was then purified by reverse phase HPLC and confirmed by electrospray ionization mass spectrometry (PNABio). The purified bisPNA-peptide conjugate was allowed to incubate with dsDNA fragments of stated lengths at a 60-fold molar excess for 2 hours at 60 C to form a dsDNA/PNA-peptide complex (10 mM sodium phosphate, pH 7.4). Excess bisPNA-peptide was cleaned up by centrifugation in a 100 kDa filter (EMD Millipore, UFC570024). Successful invasion of the bisPNA-peptide onto dsDNA of different lengths was verified via electrophoretic mobility shift assays (EMSA) on a 6% TBE gel (ThermoFisher, EC62652) at 100 V for 20 minutes then 150 V for 80 minutes (Supplementary Figs S8, S12 and S13). The anti-V3 antibodies 447D (Los Alamos National Labs, USA) and HGD65 (cat. # Ab00270, Absolute Antibody Limited, UK) were used as model antibodies. ELISA were performed to test affinity between the antibodies and the PNA-peptide and peptide alone (Supplementary Secs. 10.1.2 and 12.3).

In nanopore experiments, antibody and detection reagents were mixed at molar ratios ranging from 0.1:1 to 20:1, and then diluted into recording buffer (1 M LiCl, 10 mM Tris-HCL, pH 8.8, 1 mM EDTA) to the reported concentrations. Tabulated reagents and detection results are reported as: 447D buffer only (Supplementary Table S3); 447D serum background (Supplementary Table S4); HGD65 buffer only (Supplementary Tables S5, S12, S19 and S50); HGD65 serum background (Supplementary Tables S26); HGD65 saliva background (Supplementary Tables S30, S37). Incubation periods varied from 10 seconds to 15 minutes with buffer only. In experiments with serum, detection reagent was incubated in 20% sample without Ab for 5 min, and spiked with Ab for 15 min or 5 min (where stated), prior to dilution into nanopore buffer. Saliva samples were not tested with 447-52D. In experiments with saliva background, HGD65 and detection reagent (217 bp DNA/PNA-peptide, 13.3 nM) were mixed at the stated molar ratios in 100 *μ*L of 1 M LiCl, 10 mM Tris-HCL (pH 8.8), 1 mM EDTA, and 10% donor saliva. Samples were incubated on the bench top at room temperature for 5 minutes or 30 seconds, then 100 *μ*L samples were diluted 10X to 1 mL with 1 M LiCl, 10 mM Tris-HCL, and 1 mM EDTA, maintaining antibody:reagent molar ratios indicated above with final concentrations of detection reagent and saliva in all samples being 1.33 nM and 1%, respectively. Saliva was collected from an HIV-negative donor individual using a 1 mL plastic transfer pipette (Kinglake #10751) and stored in a 1.5 mL Eppendorf tube on ice. Saliva was warmed to room temperature and vortexed to resuspend settled particles before use.

### TNF*α* assay

The affibody against human TNF*α* (Affibody AB, Catalog No. 10.084101.0050) contains a C-terminal cysteine with a sulfhydryl group used to conjugate to the N-terminus of a bisPNA via an intermediate succinimidyl 4-(N-maleimidomethyl)cyclohexane-1-carboxylate (SMCC) linker. The bisPNA (PNABio) has sequence detailed in Supplementary Information Sec. 13.1.1. To form a dsDNA-PNA-affibody complex, the purified affibody-bisPNA conjugate was allowed to incubate with a 309 bp dsDNA fragment from the aforementioned template DNA, containing the complementary sequence to the Watson-Crick strand of the bisPNA at a 60-fold molar excess for 2 hours at 60 C (10 mM sodium phosphate, pH 7.4). Excess bisPNA-affibody was cleaned up by centrifugation in a 100 kDa filter (EMD Millipore, UFC570024). Successful invasion of the bisPNA-affibody onto dsDNA was verified via EMSA on a 6% TBE gel (ThermoFisher, EC62652) at 200 V for 40 minutes (Supplementary Fig. S27). Following purification of the binding reagent, it was allowed to incubate with a titration series of human TNF*α* (ThermoFisher, PHC3016) for a period of 1 hour at room temperature (10 mM sodium phosphate, pH 7.4). Confirmation of TNF*α* binding was conducted on a similarly run TBE gel. Following the incubation period between 50 nM scaffold/fusion and each TNF*α* concentration, the reagents were diluted 16.7-fold into nanopore buffer for experimentation. To compare *K*_*d*_ quantification values and salt tolerance between the affibody and TNF*α*, sandwich ELISA was performed and compared to nanopore results (Supplementary Information Sec. 13.1.2). Tabulated reagents and nanopore detection results are reported in Supplementary Table S52.

### Tetanus toxin assay

A 712 bp DNA (biotin-712-thiol) was made in house using PCR from the aforementioned template DNA. The forward primer was modified to incorporate biotin on one end of the DNA and the reverse primer was modified to incorporate a thiol on the other. The thiol modified end of the DNA was utilized to attach it to an anti-Tetanus toxin IgG1 antibody (*α*-TTAb) utilizing SMCC/NHS-ester chemistry. The biotin modified end of the DNA was utilized to attach the DNA-antibody conjugate to Streptavidin coated magnetic beads. A restriction enzyme digest site was engineered at the biotin conjugated end of the DNA to allow for cleavage of the DNA-antibody conjugate from the streptavidin beads after Tetanus toxin (TT) (List Biological Laboratories, Inc. Cat #190B) binding in plasma. The DNA-Ab conjugate was prepared by first reacting the *α*-TTAb with sulfo-SMCC (sulfosuccinimidyl 4-(N-maleimidomethy)cyclohexane-1-carboxylate; Thermo Fisher, Cat #22322) in order to install a maleimide. Briefly, the *α*-TTAb was diluted to 100 *μ*M in 30 *μ*l of 10 mM sodium phosphate pH 7.4 (NaPi) followed by the addition of 0.5 *μ*l of 70 mM sulfo-SMCC (in DMSO) to achieve a 10:1 molar ratio of SMCC to *α*-TTAb. The reaction was incubated at room temperature for 1 h. The *α*-TTAb-maleimide adduct was then purified using cation exchange liquid chromatography and concentrated to 50–100 *μ*M using a 30 Kd molecular weight cutoff spin column. The purified *α*-TTAb-maleimide was then reacted with biotin-712-thiol. Biotin-712-thiol was diluted in 10 mM NaPi, followed by the addition of *α*-TTAb-maleimide at a final concentration of 5–10 *μ*M (200:1 *α*-TTAb-maleimide to DNA). The reaction was incubated at room temperature for 1–2 h. Streptavidin coated magnetic beads (Invitrogen Cat # 65001) were washed 3X with magnetic bead wash buffer following the protocol provided with the beads. 5 *μ*g of the biotinylated DNA-antibody conjugate were incubated with 1 mg of the washed beads overnight at 4 °C to ensure complete conjugation to the beads. The beads were washed 3X with 100 *μ*l NaPi, and divided up into the appropriate volume to yield aliquots containing 280 ng of DNA-antibody conjugate bound to beads. The 280 ng aliquots were then used for all TT nanopore experiments without and with spiked plasma. TT studies with plasma were carried out using 20 nM 712 bp DNA or DNA-antibody conjugate (280 ng) on bead with a final concentration of 600 nM TT in NaPi and pH 7.4. Absent plasma, TT studies were carried out using 50 nM DNA-antibody conjugate with a final concentration of 75 and 200 nM TT. Control sample sets included 712 bp DNA plus TT, DNA-antibody conjugate without TT and DNA-antibody conjugate with TT. Where stated, the control samples were spiked into 10 or 20% pooled human plasma. All samples were incubated at room temperature for 45 minutes. After incubation the samples were washed 1X with 100 *μ*L NaPi, removing plasma background. Following the wash, samples were cleaved off the bead by restriction enzyme digest (New England BioLabs Cat #R3193L) yielding DNA with a length of 594 bp. Post cleavage, reagents were quality checked using EMSA (Supplementary Fig. S30) to confirm full complex formation (10% polyacrylamide, 120 mV in 1x TBE for 45 min). The cleaved samples were diluted into lithium chloride buffer for nanopore analysis at the final concentrations reported in Supplementary Tables S56 and S57.

### Nanopore experiments

Nanopore chips and pore diameter were fabricated and characterized at the Stanford Nanofabrication Facility and Nano Shared Facilities, following the process flow and methods described in the Supplementary Information. Experiments were conducted at 23 °C in 10 mM Tris with 1 mM EDTA, at pH 8.8, and 1 M LiCl, with either a custom flow cell or the test strip. Using commercially available equipment, a voltage-clamp amplifier (MultiClamp 700B, Molecular Devices, Sunnyvale, CA) was used to apply transmembrane voltage and measure ionic current, with the 4-pole Bessel filter set at 30 kHz. A digitizer (Digidata 1440A, Molecular Devices) stored data sampled at 250 kHz. The hardware comprised within the handheld Molecule Occlusion Meter (MOM) (30 kHz bandwidth, 100 kHz sample rate, Supplementary Methods). Each reagent was added at the reported concentration into the voltage-negative chamber during nanopore experiments. The 3D printed test strips (Formlabs Form 2 printer, Clear resin, FLGPCL02, Supplementary Methods) comprised 50 *μ*L channels, with the reagent-loaded channel flushed with 200 *μ*L buffer in between reagents. Channel overflow was removed via Kimwipe (KIMTECH). Buffer only is recorded for 5 minutes. If the buffer only event rate exceeds 2 min ^−1^, which was uncommon, the channels are re-flushed and the buffer-only recording restarted to ensure sufficiently clear channels between reagents.

### Data processing

All numerical analysis and data processing was done offline using custom code written in Matlab (2017, The MathWorks). Recording with the 700B/Digidata setup used the AxoScope software (Molecular Devices). Real-time event detection was done on the MOM. Plotting and recoding of data was done with custom software termed the Pore Operating Program (POP, Python). Events were flagged and extracted if any sample fell below 6 times the standard deviation (*σ*) of the open channel signal, with *σ* computed using 500 event-free samples and computed once for every 500,000 sample window. Each extracted event contains: (i) all samples adjacent to the sample(s) below 6*σ* up to the first samples below 1*σ*, and (ii) adding 200 samples prior to and after each event. Extracted events were rejected from subsequent analysis if they do not return to within 1*σ* (e.g., by truncation during data recording), or if the noise exceeds 3*σ* in the adjacent (open channel) samples. The open channel conductance values were used to track the evolution of the estimated nanopore size^36^. The reported event duration is the time-width at half maximum depth. Each open channel duration is the time between every pair of kept extracted events, and these durations were used to compute the capture rate by fitting an exponential distribution to the data. The same fitting method was applied for the subset of tagged events to quantify tagged capture rate, where reported and modeled. The mathematical methods for assigning a positive result for target detection, the corresponding time-to-positive, and for quantification of target concentration are detailed in the Supplementary Information.

## Electronic supplementary material

Supplementary Information

## Data Availability

All relevant data are within the paper and its Supplementary Information files. Data for the main figures and for Supplementary Information tables and figure are available at 10.6084/m9.figshare.7131695.
