# Supplementary material for: A handheld platform for target protein detection and quantification using disposable nanopore strips
Source: Sci Rep. 2018 Oct 4;8:14834. doi: 10.1038/s41598-018-33086-7 (PMC6172217; doi:10.1038/s41598-018-33086-7)
Supplement: Supplementary file 1 — Supplementary Information [file 41598_2018_33086_MOESM1_ESM.pdf]

Supplementary Information for "A handheld platform for target protein detection and quantitation using disposable nanopore strips"

Revised: February 22, 2018

Authors: Trevor J. Morin, William L. McKenna, Tyler Shropshire, Dustin A. Wride, Joshua D. Deschamps, Xu Liu, Reto Stamm, Hongyun Wang and William B. Dunbar.

Contents

1 The test strip **3**

2 The nanopore chip **4**

3 The Molecule Occlusion Meter (MOM) **5**

4 Low-pass refiltering **7**

5 Device performance comparisons **8**

6 Test (+/−) for the presence of target with statistical confidence, and time-to-positive (TTP) **12**

7 Estimating target concentration from tagged fraction **13**
7.1 Modeling the tagged fraction as a function of target to scaffold/fusion ratio X 13
7.2 Target concentration estimation 15

8 Estimating target concentration from tagged capture rate **16**
8.1 Modeling the tagged capture rate as a function of X 16
8.2 Target concentration estimation 17

9 Estimating target concentration with reduced (two-stage) workflow **17**

10 Detailed results with HIV Ab 447-52D **19**
10.1 Methods 19
10.1.1 Construction of HIV Ab detection reagents 19
10.1.2 HIV Ab capture ELISA 19
10.1.3 Nanopore experiment incubation conditions 20
10.2 Pores P1-P3: Standard holders and bench top amplifiers 21
10.3 Pores P4-P6: Test strips, MOM and serum background 25

11 Detailed results with HIV Ab HGD65 28
11.1 Methods 28
11.2 Pores P7-P10: Standard holders and bench top amplifiers 28
11.3 Pores P11-P13: Standard holders and MOM amplifiers 38
11.4 Pores P14-P17: Holders vs. test strips, and shorter scaffolds 43
11.5 Pores P18-P20: Detection above serum background 50
11.6 Pores P21-P24: Detection and quantitation above saliva background 52
11.7 Pores P25-P26: Detection and quantitation above saliva background with 30 second incubation 58

12 Summary of HIV Ab HGD65 results 64
12.1 Single pore quantitation performance 64
12.2 Combining estimates to improve quantitation performance 67
12.3 Exploring detection limits (P27, P28) and binding affinity 74
12.4 Detection and quantitation ranges 76
12.5 Time-to-positive and sample-to-result times 78

13 Detailed results with TNFα 80
13.1 Methods 80
13.1.1 Construction and verification of TNFα detection reagent 80
13.1.2 TNFα sandwich ELISA 81
13.2 Pores P29-P31: Detection and quantitation 82
13.3 Range and detection limits without optimization 86

14 Detailed results with Tetanus Toxin (TT) 87
14.1 Methods 87
14.2 Pores P32-P34: Detection 89
14.3 Pores P35-P36: Detection following on-bead incubation in plasma 91

1 The test strip

The test strip is shown in Figure S1 in assembled and exploded views. The fluid path for each of the two channels (0.9 mm diameter) is colored for visualization. Each of the two channel-containing parts are 3D printed on a Formlabs Form 2 printer with Clear resin (FLGPCL02) at 50 µm resolution. Post printing, the two channel-containing parts are cleaned with 99.5% IPA using a Waterpik water flosser (WP-672), flushed out with deionized (DI) water, blown dry with N_2, then cured at 65C for one hour in a Mechanical Convection Oven (DKN 600 by Yamato) that has been instrumented with two 24 W 395nm-405nm LED strips (AMARS). The two electrode seal compressions are also 3D printed. Each compression creates positive pressure between AgCl electrode pieces, an intermediate Viton gasket cut for fluid path access control, and a u-turn portion of the fluid channel path. This creates a leak-free seal, and allows the patch clamp circuitry to access the fluidics and nanopore through the AgCl electrodes. The gaskets are center cut with a 50W CO2 CNC Laser cutter (Redsail M500), then Piranha cleaned and rinsed with DI water. The electrodes are made by taking a silver strip (.999 Fine Silver 3/16" Bezel Strip, 28-Ga., Dead Soft by Rio Grande), cleaning it with IPA on both sides to remove oily residues, covering one side with electrical tape (Scotch #35 Electrical Tape), bleaching it overnight, then washing it with DI, removing the tape, and cutting them to size with scissors. The nanopore chip is sealed with two Viton gaskets that are center laser cut (Redsail M500) for fluid path access control. Four screws and fitted nuts are used to create the positive pressure around the gasket/chip/gasket sandwich, to ensure leak-free sealing such that electrical conductivity between the channels can occur only through the nanopore. Test strips can be re-cleaned and reused any number of times, with new gaskets, electrodes and/or nanopore chip; alternatively, the strip can be disposed of after a single experiment. The cost of goods for the 3D printed strip without the nanopore chip is $3.80. Regarding projected cost reductions, we have subsequently switched to a lamination process with printed electrodes resulting in $0.30/strip in material cost. High volume manufacturing methods (e.g., injection molding) can reduce costs further still.

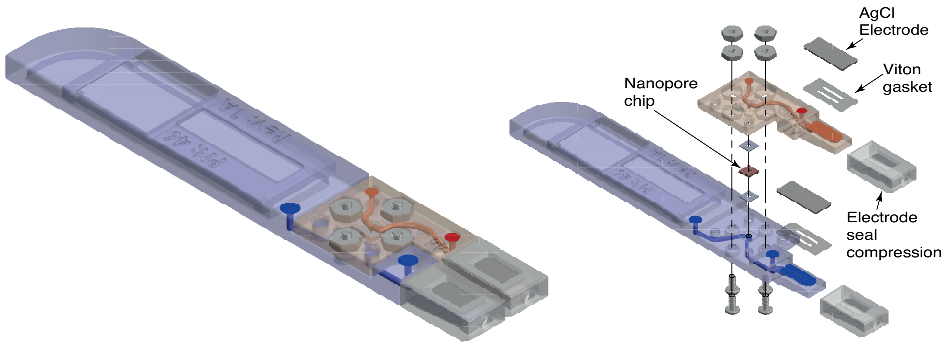

Figure S1: Schematics of the assembled (left) and exploded (right) test strip CAD drawings. The test strip is 7.5 cm in length, 1.5 cm wide and 0.4 cm thick.

2 The nanopore chip

The fabrication methodology of the low-noise nanopore chips is depicted in Figure S2a. Briefly, 30nm of low-stress low-pressure CVD (LPCVD) SiN thin film (<200MPa, tensile) was first deposited on 350 µm Si substrates. To reduce noise, an insulation layer, consisting of a 2 µm SiO2 layer was deposited on the front side of the wafer using a plasma-enhanced CVD (PECVD) process. An additional 400 nm SiN etch mask layer was deposited via LPCVD on the substrate following the SiO2 deposition. The etch pit was open from the backside by photolithography followed by reactive ion etching of the SiN etch mask layer and KOH wet etching of the Si substrate. A second photolithography step was performed on the front side of the wafer to define the SiO2 micro-well pattern. Subsequently, reactive ion etching was used to partially open the SiO2 layer with target etch depth of 1.8 µm. The SiN membrane was then fully released by removing the remaining oxide material in the micro-well with 3 minutes of HF wet etch. Finally, nanopores are formed in the SiN membrane inside the micro-well using focused ion beam (FIB) (FEI, Helios Nanolab 600i). A representative nanopore is shown in Figure S2b. The chip (3 mm x 3 mm) without a nanopore currently costs $3 at low volumes (4” wafers). The serial FIB pore forming process used in this study costs $30 per nanopore. A more recently developed e-beam lithography process produces pores with equal performance to FIB pores at negligible additional cost. Projected costs of nanopore chips will be significantly lower by using high volume manufacturing methods.

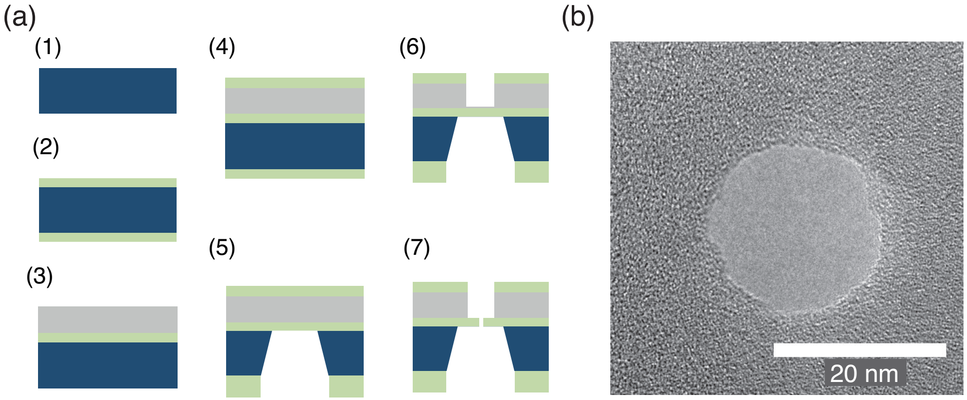

Figure S2: (a) Nanopore fabrication process flow schematics: (1) starting silicon substrate; (2) 30nm low stress LPCVD SiN deposition; (3) 2 µm PECVD SiO2 deposition; (4) 400nm LPCVD SiN deposition; (5) backside lithography followed by RIE and KOH wet etch; (6) front side lithography followed up RIE and HF wet etch; (7) FIB drill. (b) Representative TEM image of a 20 nm diameter nanopore.

3 The Molecule Occlusion Meter (MOM)

The handheld reader device is referred to as the Molecule Occlusion Meter, or the "MOM." The MOM has a two-sided board mounted to a shielding box (Fig. S3) that the test strip is inserted into electrode-side up. The reader shown in the main text Figure 1 has an additional clear housing around the MOM, which optionally adds protection (e.g., from spillage).

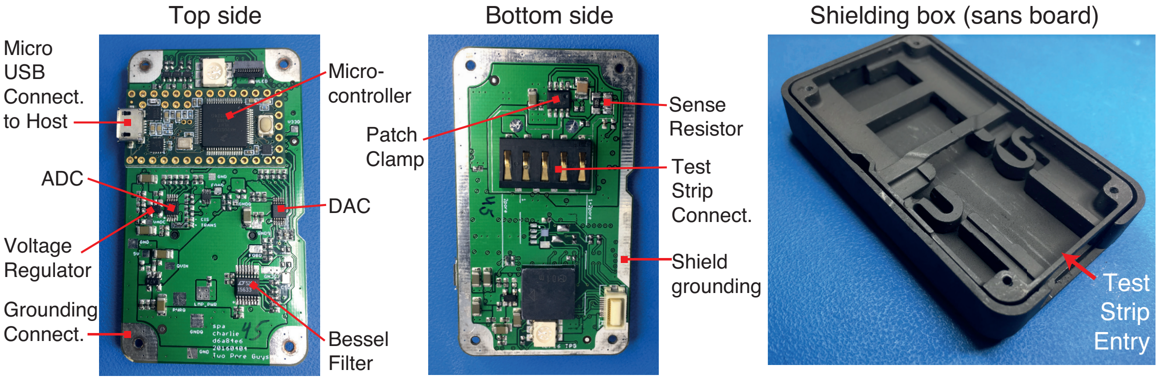

Figure S3: Breakout view of the two-sided board (7 cm x 4 cm) and the shielding box that make up the MOM. When assembled, the board is fastened onto the shielding box, top-side up. During nanopore sensing, two of the connection tabs (2nd and 4th) on the bottom side of the board make contact with the two test strip electrodes.

As with the test strips, the shielding box is 3D printed on a Formlabs Form 2 printer with Clear resin (FLGPCL02) at 50 µm resolution. The shielding box is sprayed with conductive acrylic carbon (838AR Total Ground Carbon Conductive Coating, M.G. Chemicals). The carbon coating helps to shield the fluidic test strip and circuitry from electrical noise sources. The amplifier is on a four layer board that measures 40mm x 70mm. The board has a grounding rim (shown in Fig. S3) that contacts with the shielding box to aid in noise reduction.

A schematic of the amplifier circuitry is depicted in Figure S4. The amplifier has a surface mounted 72 MHz Cortex-M4 based Teensy 3.2 microcontroller (PJRC) that receives control commands through USB from the PC based software. The firmware on the Teensy processes commands that are sent from the host computer, and sends acquired data back to the host for storage and display. When the teensy receives a set voltage command from the PC over USB, the firmware sets the DAC output (DAC8562FSZ by Analog Devices). The patch clamp amplifier (LMP7721MA by Texas Instruments, with a 10 MΩ sense resistor) sets the voltage across the pore. The drive voltage of the patch clamp is filtered by a 30kHz low-pass Bessel filter (LTC1563 by Linear Technology Corporation), then read by the differential ADC (AD7688BRMZ-ND by Analog Devices). The firmware on the Teensy pulses the Conversion (CNV) signal at 100kHz. An interrupt triggered by the same timer reads the sample from the ADC over a second SPI bus and stores it in a ring buffer. In the background the Teensy sends 64-byte data over the USB bus to the host computer where they are displayed by software (Python) and written to disk. The assembled MOM unit cost of $74.50 is primarily driven by the parts (Table S1).

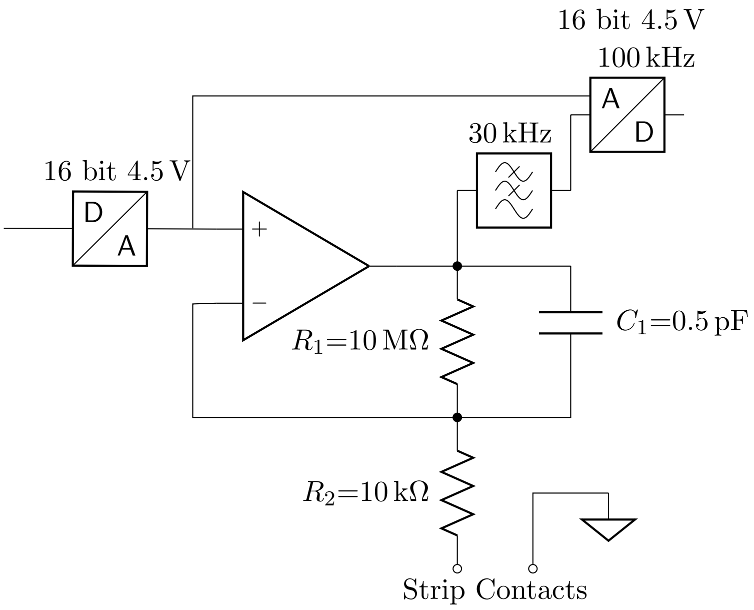

Figure S4: Summary schematic of the amplifier circuitry within the MOM. The OpAmp output voltage changes with pore impedance change, as measured through the test strip electrodes (i.e., the strip contacts). It is then filtered by a four-pole low pass Bessel filter at 30 kHz bandwidth and read with the ADC.

Table S1: **Partial bill of materials for the MOM board**

Purpose	Part	Manufacturer	Cost/u
Microcontroller	Teensy 3.2	PJRC.com	$15.84
DAC	DAC8562FSZ	Analog Devices	$12.41
Clamp OpAmp	LMP7721MA	Texas Instruments	$2.49
Bessel Filter	LTC1563	Linear Technology Corporation	$2.30
ADC	AD7688BRMZ-ND	Analog Devices	$20.71

4 Low-pass refiltering

To further reduce the standard deviation of the recorded signal, digital low-pass filtering is used. The signal is recorded at 30 kHz bandwidth with a 4-pole low pass Bessel filter, and sampled at 100 kHz with the MOM or 250 kHz with the 700B amplifier. A desirable feature of the analog low-pass Bessel filter used prior to digitization is that it will distort a pulse-like signal underlying the noise much less than other conventional low-pass filters (e.g., Butterworth, Chebyshev). To preserve this feature, we prefer to refilter the digitized signal using a discretized low pass Bessel filter. A naive discretization of the analog Bessel filter (e.g., bilinear transform) would eliminate the minimal pulse-distortion feature of the filter. To avoid this, the analog filter is represented in linear continuous state-space form, then solved analytically using the matrix exponential [1] at each sample time, resulting in a discretized refiltering equation that emulates the analog filter. The refiltering equations require only the sample period and bandwidth values to be solved. The digitized signal fed into the refiltering equation is treated as piece-wise constant, though a piece-wise linear approximation generated the same solution to within machine precision (i.e., a piece-wise constant assumption is reasonable). The standard refiltering bandwidth value used in the paper is 10 kHz for all data recorded with the MOM.

5 Device performance comparisons

This section compares the electrical and nanopore sensing performance of the MOM with the Molecular Devices 700B amplifier, using a common test strip. The chip in the test strip had a 22 nm diameter pore. As in [2], experiments with the 700B were performed in a standard Faraday cage setup, and connecting the head-stage wires directly to the electrode contacts on the test strip. As a first comparative measure of noise, the power spectral density (PSD) estimates were computed via Welch's method (Hamming window, 50% overlap) applied to the baseline current at 100 mV after refiltering at 10 kHz bandwidth and subtracting the mean of the signals. The refiltering method is detailed in Section 4. Comparison of the two PSD estimates using the same test strip is shown in Figure S5a. The sharp upward peaks in the MOM PSD between 2 and 20 kHz is a result of picking up noise from environment sources, including the microprocessor on the board, as well as light sources affecting the reader through it's translucent casing (main text Figure 1). Subsequent improvements have eliminated these peaks. In any case, it is not discrete peaks in the PSD but the aggregate (integrated) noise power that determines the sensing resolution limit of the setup, as discussed next.

The sensing resolution limit is defined by the standard deviation of the signal at the chosen bandwidth. As discussed in [2] (SI), the bandwidth determines the temporal resolution limit. Specifically, detection requires event durations of at least the rise time of the total bandwidth, and amplitude quantitation requires event durations of at least twice that rise time. The rise times are 12 μs at 30 kHz (recording) and 36 μs at 10 kHz (refiltered) bandwidths. The detection threshold is set at 6 times the standard deviation of the open channel signal, computed using 400 samples to ensure convergence [2]. Thus, detectable events must be long enough in duration (relative to the bandwidth rise time) and deep enough in attenuation (relative to detection threshold). The standard deviation is a cumulative measure of noise. The elevated PSD of the MOM compared to the 700B over the range of frequencies (10^2 to 10^4 Hz in particular) suggests that the MOM will have a higher standard deviation, and thus lower sensing resolution, than the 700B. This was observed to be the case in experiments, with the MOM having standard deviations 13-16 pA at 10 kHz and 25-35 pA at 30 kHz bandwidths, and the 700B having having standard deviations 6-9 pA at 10 kHz and 15-20 pA at 30 kHz bandwidths (Fig. S5b). Nevertheless, the MOM and test strip performance are still sufficient for the detection and analysis work described in this paper.

The MOM and test strip performs comparably to a recently published noise optimized nanopore chip [3] that used an Axopatch 200B, an amplifier equivalent in manufacturer (Molecular Devices) and performance to the 700B. A caveat to making the comparison with that work is that they examined a smaller nanopore diameter and in thinner membranes (up to 20 nm vs. our 30 nm membrane). First, we can compare estimates for the nanopore chip capacitance. The capacitance estimation method we employ is comparable to that in [4]. In brief, the system transfer function has two known dominant poles (sense resistor, stabilizing RC filter) and the unknown zero at C_pR_p, where R_p is the nanopore resistance found from fitting slope to voltage vs. current (linear) data, and C_p the nanopore capacitance to be estimated. Next, the integral of the step-response is numerically computed following $\pm$10 mV steps, after shifting each post-transient steady-state baseline to zero mean. The integral values, known parameters and integral equation are used to solve for C_p for each step response, and the set of C_p values are averaged to estimate capacitance for the nanopore chip. The test strip and MOM in Fig. S5 generated a capacitance estimate of 20.5 pF, which is lower than most of the noise-optimized chips in [3] (4 out of 5 were above 40 pF, SI Fig. 2). The lower capacitance is likely due, in part, to our chips having thicker membranes, though no change

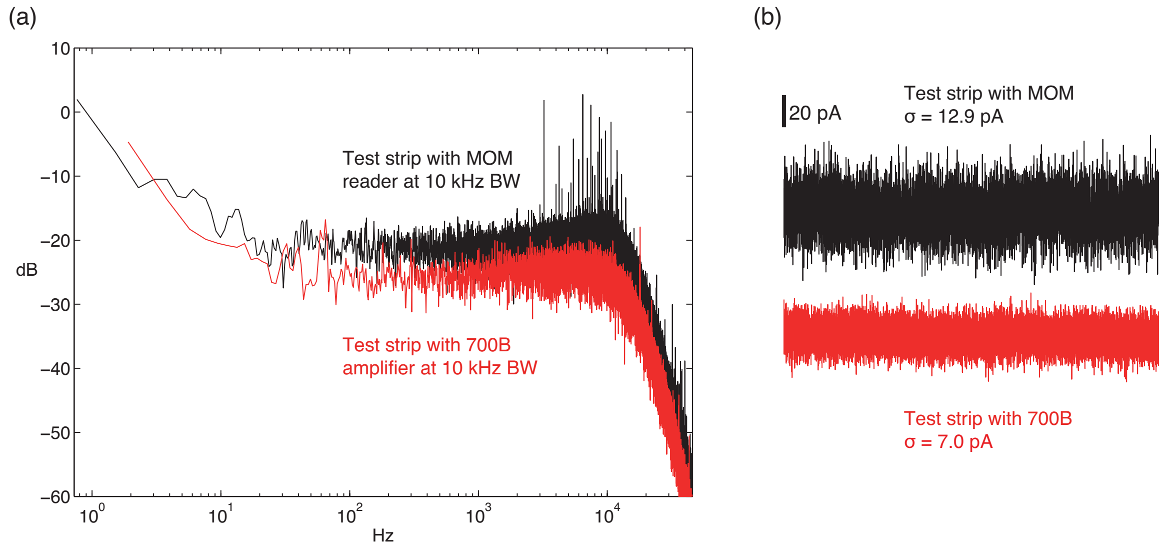

Figure S5: Comparison of (a) the power spectral density (PSD) estimates (500,000 samples) and (b) open channel current (1 second) for a test strip using the MOM and the Molecular Devices 700B. Recording conditions are in Table S2. (a) The dB units are 10*$\log_{10}$ (pA^2/Hz), so -20 dB is 10^{-2} pA^2/Hz. (b) σ reports the standard deviation at 10 kHz bandwidth.

in capacitance was observed with varying membrane thickness up to 20 nm in [3]. Additionally, with an applied voltage to generate 4.5 nA of current at 10 kHz bandwidth, the RMS noise is 12.6 pA in [3] for the 20 nm membrane, whereas the test strip and MOM achieve the comparable value of 12.9 pA RMS at 10 kHz bandwidth and 8.5 nA, twice as much current due to the larger pore size (Fig. S5b). The authors noted that their observed noise level is "... comparable to (other) modified low-noise solid-state devices[22-24,27,32]," suggesting that the MOM and test strip perform comparably to the state of the art in low-noise solid-state nanopore devices.

Other recent studies present amplifier designs that have significantly lower noise at high frequencies [5, 6, 7, 8]. This is particularly valuable, provided noise at lower frequencies does not increase. The design in [5] was compared to the Axopatch 200B, for example, and although it showed lower noise at high frequencies, the noise at lower frequencies was comparatively increased. To utilize larger nanopores in applications, smaller event signals must be resolvable above the noise, and this requires the lowest possible RMS at acceptable cost in temporal resolution. Moreover, as discussed in the introduction, the value of using larger pores is that they can be made at high volumes inexpensively by utilizing widely available fabrication equipment. With the exception of controlled dielectric breakdown in sufficiently thin membranes [9, 10], smaller pores (<10 nm) cannot be made at high volumes using widely available fabrication equipment. Thus, breakthroughs in circuitry for nanopores should push the noise floor down unilaterally to the extent possible. The circuit and nanopore setup in [6] showed 23 pA RMS at 200 kHz bandwidth as the lowest RMS displayed, which is slightly better than the MOM at 30 kHz and at 7X improved temporal resolution. This suggests that the design elements in this and other custom amplifiers could dramatically improve the MOM, provided costs could be kept at low levels through the use of integration [11, 8]. A commercial amplifier inspired by [5] is the Chimera Instruments VC100, which has a retail price of $16,500, identical in price to the Molecular Devices 700B (a two channel amplifier). Molecular

Devices software and high-channel digitizer are an additional \$10k. These prices are acceptable for research, but not for widespread commercialization of nanopore technology.

We also compare the sensing resolution of the MOM and the 700B with DNA detection and quantitation experiments. Using the same test strip from Figure S5 data, 1 nM 1074 bp DNA was measured at 100 mV for 5 minutes, first with the MOM and then the 700B. The DNA was removed by flushing, followed by 1nM 5.6 kbp DNA measured at 100mV for 5 minutes, first with the MOM and then the 700B. The maximum δG vs. duration event plots for 1074 bp and 5.6 kbp dsDNA experiments are shown in Figure S6. The corresponding event population statistics and standard deviations are reported in Table S2. The maximum δG population is bimodal for 5.6 kbp DNA since it is long enough to pass through the pore (22 nm diameter) in a folded state more often than the 1074 bp DNA. In the summary statistics, the aggregate (mean) is computed for the mean δG values. The purpose of the statistics reported in Table S2 is for comparison between the amplifiers, not as an attempt to model the distributions.

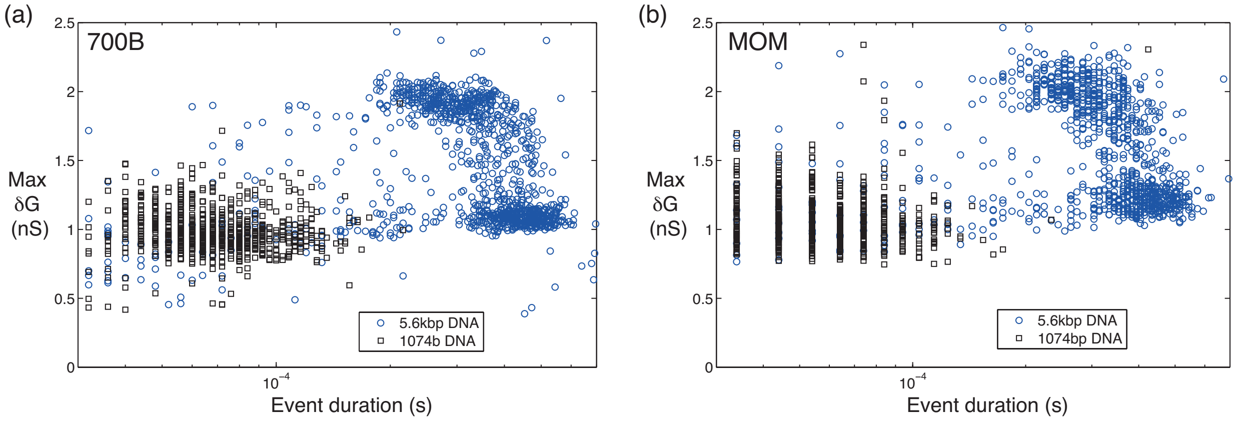

Figure S6: An all-event scatter plot of maximum δG versus duration, measuring 1074bp and 5.6 kbp DNA using (a) the 700B amplifier and (b) the MOM. The longer sample period for the MOM (10μs) results in events that are more quantized in duration than for the 700B (4μs sample period), an effect that is more pronounced as event durations come closer to the sample period.

The summary statistics in Table S2 on the 5.6 kb DNA event populations show essentially equivalent performance between the MOM and the 700B, since the population is far from the resolution limit of both setups. The temporal sensing resolution limit for the MOM and 700B are the same by using the same 10 kHz bandwidth. Specifically, with a rise time of 36μs, event durations must be at least 36μs to hit 75% full depth and at least 72μs to hit 100% full depth. Duration information is more quantized for the MOM, however, due to the lower sample rate (100 kHz). Most of the 5.6 kb events are above the full-depth duration limit, making the two populations indistinguishable. More than half of the 1074 bp DNA events, on the other hand, do not hit the full depth limit; as a result, more events will not meet the detection threshold for the MOM than for the 700B, due to the higher standard deviation. Specifically, the amplitude detection threshold is 6 times the standard deviation: $\sim$42 pA (0.42 nS) for the 700B and $\sim$78 pA (0.78 nS) for the MOM. The lower event rate for the MOM shows that a modestly larger fraction of 1074 bp events are passing undetected compared to the 700B. Although 1074 bp DNA and shorter DNA are used in this work, and such molecules generate event populations that are trimmed due to the resolution

Table S2: **Comparison of 700B and MOM amplifiers for DNA detection and quantitation using a common test strip.**

Reagent	1074 bp	1074 bp	5.6 kbp	5.6 kbp
Amplifier	700B	MOM	700B	MOM
No. Events†	774	677	1077	1049
Capture Rate‡	154.3 min^{-1}	134.1 min^{-1}	213.2 min^{-1}	217.2 min^{-1}
Mean{mean δG}	0.81 ± 0.19 nS	0.93 ± 0.21 nS	1.05 ± 0.26 nS	1.11 ± 0.26 nS
Median{duration}♮	68 ± 28 μs	54 ± 30 μs	348 ± 180 μs	334 ± 150 μs
Signal σ^a	6.8 ± 0.7 pA	13.0 ± 0.8 pA	7.0 ± 0.7 pA	12.9 ± 0.8 pA

† Experiment details: 1 nM DNA; 100 mV; 5 minutes of recording; buffer was 1M LiCl, 10mM Tris, 1mM EDTA, and pH=8.8; 22 nm diameter pore.

‡ Capture rate was found by fitting an exponential distribution to the time-to-capture [2] with R^2 fit performance (l-to-r): 0.998, 0.998, 0.997 and 0.999.

♮ Median is reported with the inter-quartile range.

^{a} The standard deviation (σ) of the open pore signal is computed for each event using the 200 samples before and after that event. Reported are the mean and standard deviation of the σ for all events.

limits of the MOM and the 700B, the relative trends (tagged percentage in particular) can still be analyzed and modeled, as detailed in this manuscript.

6 Test (+/−) for the presence of target with statistical confidence, and time-to-positive (TTP)

The mathematical framework upon which the presence/absence test is derived is presented in [2] (SI), with salient details recounted here for completeness. We compute the fraction of tagged events:

$$Q = \frac{\text{Number of tagged events}}{\text{Total number of events}} = \frac{1}{N}\sum_{j=1}^{N} X_j \tag{1}$$

where N is total number of all events (tagged or not), and X_j is a 0 or a 1 if event j is untagged or tagged, respectively. Generically, the tagging criteria can be based on any one or more event parameters. In this study, the criteria is based on exceeding a maximum δG threshold, since a distinct subpopulation of deeper events are observed for each of the targets considered when the full complex is present. In some instances with sample background present, a minimum duration criteria is also added to the (minimum) maximum δG threshold tagging criteria. The methods below are presented for the maximum δG threshold case, but apply to any combination of event criteria.

To identify the maximum δG threshold above which events are tagged, one of two methods is applied. The first method is nanopore specific, and the second method aggregates information across more than one nanopore. In the first method, one or more negative controls is run on each nanopore, and the threshold is defined as the maximum δG value such that the largest Q for the negative controls satisfies $Q \leq Q_{\text{f.p.}}$. The $Q_{\text{f.p.}}$ value is the false positive fraction. In the nomenclature of ROC analysis, $Q_{\text{f.p.}}$ represents a maximum false positive event rate, since it represents an upper bound on the number of incorrectly classified (i.e., tagged) negatives to the total number of negatives [12] (a deeper ROC analysis is outside the scope of this work). Nominally, $Q_{\text{f.p.}} = 0.02$ for a false positive of 2%. The second method picks a specific maximum δG threshold such that all negative controls across all pores being aggregated satisfy $Q \leq Q_{\text{f.p.}}$. The second method is more conservative, since pores with lower Q values in negative controls could use a lower threshold and thus achieve a faster positive result than when using an aggregate (higher) threshold. The tradeoff is that the second method would be simpler to implement, e.g., if a look-up table can provide the tagging criteria based on the nanopore size measured *in situ* using the method described in [2] (SI). Once the maximum δG threshold for tagging is established, we apply a positive/negative test for the presence of full complex (and thus the presence of target) to each subsequent reagent being measured on the pore.

The test is the following: using the computed Q value, a reagent is **positive for full complex with 99% confidence if the following is true**

$$Q - 2.5758\sqrt{\frac{Q(1-Q)}{N}} > Q_{\text{f.p.}}. \tag{2}$$

If (2) **is not true, the result is negative**. For 95% confidence the 2.5758 coefficient is replaced with 1.96, and the general function form for any desired confidence level is defined in [2] (SI). Importantly, the Q value in (1) can be computed in real-time as events are being detected and quantitated. When reporting $Q \pm Q_{\text{conf}}$ in the paper, the error margin is $Q_{\text{conf}} = q\sqrt{Q(1-Q)/N}$, where $q = 2.5758$ (or 1.96) for 99% (or 95%) confidence.

The **time-to-positive** (**TTP**) is the first time at which (2) is true and remains true. If (2) is never true or only transiently true (i.e., becoming false after true) for a reagent over the stated recording period, the result is negative and "np" (i.e., not positive) is stated.

7 Estimating target concentration from tagged fraction

7.1 Modeling the tagged fraction as a function of target to scaffold/fusion ratio X

We model the binding reaction between scaffold/fusion and target biomarker in the bulk solution:

$$A + B \longleftrightarrow AB$$

where A = scaffold/fusion and B = target biomarker. Let

$[A]_0$ = total concentration of A;

$[B]_0$ = total concentration of B;

$X = [B]_0/[A]_0$, target to scaffold/fusion concentration ratio;

K_d = binding affinity of A with B.

In experiments, concentration $[A]_0$ is fixed and known, and K_d is known or found through parameter fitting to data. The concentration $[AB]$ is governed by the equilibrium

$$([A]_0 - [AB])\,([B]_0 - [AB]) = [AB]K_d$$

The concentration $[AB]$ as a function of $[B]_0$ has the expression

$$[AB] = f([B]_0) = \frac{[A]_0}{2}\left\{1 + \frac{[B]_0}{[A]_0} + \frac{K_d}{[A]_0} - \sqrt{\left(1 + \frac{[B]_0}{[A]_0} + \frac{K_d}{[A]_0}\right)^2 - 4\frac{[B]_0}{[A]_0}}\right\}$$

The fraction of target-bound scaffold/fusion molecules in bulk

$$F_{\text{bound}} = \frac{f([B]_0)}{[A]_0} = \frac{1}{2}\left\{1 + \frac{[B]_0}{[A]_0} + \frac{K_d}{[A]_0} - \sqrt{\left(1 + \frac{[B]_0}{[A]_0} + \frac{K_d}{[A]_0}\right)^2 - 4\frac{[B]_0}{[A]_0}}\right\}$$

The fraction of target-bound scaffold/fusion molecules going through the nanopore is

$$F_{\text{bound}}^{(\text{pore})} = \frac{r_{\text{b}}F_{\text{bound}}}{r_{\text{ub}}(1 - F_{\text{bound}}) + r_{\text{b}}F_{\text{bound}}}$$

where

r_{b} = nanopore capture rate per concentration of target-bound scaffold/fusion molecules AB.

r_{ub} = nanopore capture rate per concentration of unbound scaffold/fusion molecules A.

We use a nanopore to capture molecules (A, B, or AB) from bulk and measure the amplitude profile as each molecule goes through the pore. The goal is to detect the presence of AB complexes and to estimate $[B]_0$, the total concentration of B. Based on event duration and amplitude signature, a capture event is classified either as tagged or untagged. We make 2 assumptions:

1. all capture events of A or B molecule are untagged with high probability (small false positive, e.g., 2%);

2. a capture event of AB molecule is tagged with probability $0 < \alpha < 1$; correspondingly, a capture event of AB molecule is untagged with probability $1 - \alpha > 0$ (rate of false negative > 0); the probability, α, may vary from pore to pore but it stays the same for the same pore.

At each X, the measured fraction of tagged events is Q while the modeled fraction of tagged events F_{tag} is

$$F_{\text{tag}} = \alpha F_{\text{bound}}^{(\text{pore})} = \frac{\alpha \frac{r_{\text{b}}}{r_{\text{ub}}} F_{\text{bound}}}{1 + \left(\frac{r_{\text{b}}}{r_{\text{ub}}} - 1\right) F_{\text{bound}}}$$

Observe that as $X \to \infty$, $F_{\text{bound}} \to 1$ and $F_{\text{tag}} \to \alpha$. We define the parameters

$$q_1 = \frac{r_{\text{b}}}{r_{\text{ub}}}, \quad q_2 = \frac{K_d}{[A]_0}.$$

The **three parameter model** $F_{\text{tag}}(\alpha, q_1, q_2)$ of the tagged fraction is a function of X, given by

$$F_{\text{tag}} = \frac{\alpha q_1 F_{\text{bound}}}{1 + (q_1 - 1) F_{\text{bound}}} = \frac{\alpha q_1 \left\{(q_2 + 1 + X) - \sqrt{(q_2 + 1 + X)^2 - 4X}\right\}}{2 + (q_1 - 1)\left\{(q_2 + 1 + X) - \sqrt{(q_2 + 1 + X)^2 - 4X}\right\}} \tag{3}$$

From a titration data set $\{Q_i, X_i\}$, $i = 1, ..., n$, with $n \geq 3$, the three parameters (α, q_1, q_2) in equation (3) are found by minimizing the scalar-valued function $||\bar{F}_{\text{tag}}(\alpha, q_1, q_2) - \bar{Q}||_2^2$. In the function, $||\cdot||_2$ is the Euclidean vector norm and $\bar{F}_{\text{tag}}$ and $\bar{Q}$ are n-dimensional vectors, with $\bar{F}_{\text{tag}} = [F_{\text{tag}}(\alpha, q_1, q_2)(X_1), ..., F_{\text{tag}}(\alpha, q_1, q_2)(X_n)]$ and $\bar{Q} = [Q_1, ..., Q_n]$. The minimization is done using a direct simplex search method. To generate a reasonable initial guess for the 3-parameter fitting method, we first set $\alpha = Q$ at the largest recorded X, and then fit (q_1, q_2) by using linearization approximations of $F_{\text{tag}}(X)/\alpha$ for small X and for large X (small X^{-1}). Specifically, for small X, $F_{\text{tag}}/\alpha \approx cX$ with $c = q_1/(q_2 + 1)$, and for small $(1/X)$, $F_{\text{tag}}/\alpha \approx 1 - d(1/X)$ with $d = q_2/q_1$. The small and large X approximation equations each use $\{Q_i, X_i\}$ values that correspond to Q_i below and above half-maximum, respectively, setting $F_{\text{tag}} = Q_i$. The linearizations are written in vector form and solved for (c, d) to generate (q_1, q_2) initial estimates.

A simplifying assumption is that the capture rate constants for scaffold/fusion without and with target bound are the same, i.e., $r_{\text{b}} = r_{\text{ub}}$ and $q_1 = 1$. Equation (3) then becomes a **two parameter model** $F_{\text{tag}}(\alpha, q_2)$, given by

$$F_{\text{tag}} = \alpha F_{\text{bound}} = \frac{\alpha}{2}\left\{(q_2 + 1 + X) - \sqrt{(q_2 + 1 + X)^2 - 4X}\right\} \tag{4}$$

From a titration data set $\{Q_i, X_i\}$, $i = 1, ..., n$, with $n \geq 2$, the two parameters (α, q_2) in equation (4) are found by minimizing $||\bar{F}_{\text{tag}}(\alpha, q_2) - \bar{Q}||_2^2$. The minimization is achieved using nonlinear least-squares due to its simplicity in implementation [1], with initial guess $(\alpha, q_2) = (1, 1/[A]_0)$.

To generate uncertainty estimates for the model fitted parameters, simulated Q_i data is drawn 500 times from the distributions $Q_i \pm Q_{i,\text{conf}}$ for each X_i, $i = 1, ..., n$, to generate 500 parameter estimates. The mean and standard error of the parameter estimates are then computed. For the two and three parameter models, cumulative computational runtimes are <5 seconds and <10 seconds, respectively. The computation time to generate parameter estimates without uncertainty estimates was at most 0.025 seconds.

For both models, an estimate for the dissociation constant K_d and its standard error can be computed from $K_d = q_2[A]_0$, using the estimate q_2 and its standard error and the known value

for $[A]_0$. The normalized root-mean-square (NRMS) error is $||\bar{F}_{\text{tag}}(\alpha, q_1, q_2) - \bar{Q}||_2/(\text{range}(\bar{Q})\sqrt{n})$, where $\text{range}(\bar{Q}) = \max_i Q_i - \min_i Q_i$, and is reported as a percentage measure of fitting performance and residual variance.

It is worth noting that while these models are derived using mass action equations that represent bulk phase binding kinetics, such models may or may not accurately reflect the single molecule dynamics that occurs at the nanopore. As such, the models are phenomenological, and the fitted K_d value may or may not reflect the true bulk phase dissociation constant.

7.2 Target concentration estimation

Generating an estimate $[B]_0^{\text{e}}$ for the total target concentration $[B]_0$ is achieved by generating an estimate X^{e} for the target to scaffold/fusion ratio X, and then computing $[B]_0^{\text{e}} = X^{\text{e}}[A]_0$ from the known value for $[A]_0$. The estimate X^{e} is generated by inverting the derived model equations and using the fitted parameters. For the three parameter model with parameters (α, q_1, q_2), the estimate for X_i in the pair (Q_i, X_i) is given by

$$X_i^{\text{e}} = F_{\text{bound}} + \frac{q_2 F_{\text{bound}}}{1 - F_{\text{bound}}}, \quad \text{with} \quad F_{\text{bound}} = \frac{Q_i}{q_1(\alpha - Q_i) + Q_i}. \tag{5}$$

For the two parameter model with parameters (α, q_2), the estimate for X_i in the pair (Q_i, X_i) is

$$X_i^{\text{e}} = F_{\text{bound}} + \frac{q_2 F_{\text{bound}}}{1 - F_{\text{bound}}}, \quad \text{with} \quad F_{\text{bound}} = \frac{Q_i}{\alpha}. \tag{6}$$

The upper bound of the predictive range is the X_i value at which the tagged fraction reaches saturation ($Q_i \geq \alpha$). Above this value, the inverted functions are undefined or ill-conditioned, and no estimate can be generated with either model. The lower bound of the predictive range is the lowest X_i such that Q_i is higher than the false-positive threshold (see equation (2)).

To generate uncertainty estimates for each X_i^{e}, 500 predictions are made using simulated data. The simulated data are drawn 500 times from the Q_i distribution and drawn 500 times from the parameter distributions. The mean and standard error of the 500 X^{e} estimates are computed and reported. Uncertainty estimates report "NaN" when any one of the simulated Q data exceed the simulated α value, since above saturation values the inversion of the model function is undefined. The simulated data for Q_i and the parameters are independently generated, i.e., the sampled parameter values are not a result of fitting to the sampled data, as a way to examine the aggregate effects of both measurement and fitted parameter uncertainties on X^{e} values. In this way, the size of the uncertainty, as gauged by the coefficient of variation in the reported results, is representative of the estimation precision when quantitating the concentration from blinded samples not used in the model fitting. To examine estimation accuracy, one would have to test blinded samples as described below, but this is beyond the scope of this study.

The X^{e} values are reported for each known X used in the model fitting. The error reported is the relative error $(X^{\text{e}} - X)/X$ converted to a percent. Such errors are a measure of how well the model fits each data point below saturation. Future work will explore the quantitation performance of estimating X^{e} for blinded samples not used in model fitting, but measured after model fitting to a control data set, toward developing and assessing a sample-to-answer workflow. To estimate unknown X^{e} from blinded samples, one would first have to run three or two controls to apply equation (5) or (6), respectively. An alternative work flow to apply equation (6) would be to

assume a previously identified value for K_d (e.g., found by ELISA using the same binding reagent). In that case, a single control could be run at a saturating value for X to approximate $\alpha = Q$, in addition to measuring Q_i for the sub-saturating X_i to be estimated.

Since prediction can be done with a model-fitted K_d value or a previously identified K_d value, a related issue is how sensitive the X^{e} estimate is to uncertainty in the value for K_d. Mathematically, it follows that $\partial X^{\mathrm{e}}/\partial q_2 = F_{\mathrm{bound}}/(1 - F_{\mathrm{bound}}) \leq 1$ for $F_{\mathrm{bound}} \leq 0.5$ for both models. Thus, uncertainty in a predicted value X^{e} will not be amplified by any uncertainty in the value for K_d when $Q \leq \alpha/2$. In other words, having an accurate value for K_d is not essential for accurate prediction of concentrations below saturation.

8 Estimating target concentration from tagged capture rate

8.1 Modeling the tagged capture rate as a function of X

The models here are nearly the same as those based on the tagged fraction. First, in addition to the two assumptions stated in section 7.1, we assume:

3. the capture rate of AB molecule is proportional to the concentration $[AB]$ with proportionality coefficient $\beta > 0$; the coefficient β may vary from pore to pore but it stays the same for the same pore.

We introduce the notation

$R_T =$ the observed tagged capture rate;

$R_{m,T} =$ the modeled tagged capture rate.

The tagged fraction modeling framework in section 7.1 compares the measured fraction Q to the model F_{tag}. Likewise, the framework in this section compares to the measured R_T with the model $R_{m,T}$. The observed capture rates of all events and for the subset of tagged events are found by fitting a single exponential function to the inter-event time distributions and reporting the R^2 value as a measure of fit performance, as described elsewhere [2]. The occurrence rate of tagged events is modeled here as

$$R_{m,T} = \alpha\beta[AB].$$

Since the parameter product $\alpha\beta$ occurs universally, the two parameters cannot be independently estimated, and the product is treated as a single parameter to be estimated. Observe that $\alpha\beta > 0$ can be larger than one, even though $\alpha \leq 1$. Following the derivations in section 7.1, we have a three parameter $(\alpha\beta, q_1, q_2)$ model:

$$R_{m,T} = \frac{\alpha\beta[A]_0 q_1 \left\{(q_2 + 1 + X) - \sqrt{(q_2 + 1 + X)^2 - 4X}\right\}}{2 + (q_1 - 1)\left\{(q_2 + 1 + X) - \sqrt{(q_2 + 1 + X)^2 - 4X}\right\}} \tag{7}$$

and the two parameter $(\alpha\beta, q_2)$ model:

$$R_{m,T} = \frac{\alpha\beta[A]_0}{2}\left\{(q_2 + 1 + X) - \sqrt{(q_2 + 1 + X)^2 - 4X}\right\} \tag{8}$$

For both models, $X \to \infty$ implies $R_{m,T} \to \alpha\beta[A]_0$. Similar to section 7.1, the parameters are found by minimizing $||\bar{R}_{m,T} - \bar{R}_T||_2^2$ from titration data sets $\{R_T^i, X_i\}$, and simulated data from the R_T distributions are used to generated uncertainty estimates for the fitted parameters. The NRMS error $||\bar{R}_{m,T} - \bar{R}_T||_2/(\text{range}(R_T)\sqrt{n})$ is reported as a percentage measure of fitting performance and residual variance.

8.2 Target concentration estimation

As in section 7.2, an estimate X^{e} for the target to scaffold/fusion ratio X is generated to compute the estimate $[B]_0^{\text{e}} = X^{\text{e}}[A]_0$ from the known value for $[A]_0$. The X^{e} estimates are generated by inverting the derived model equations and using the fitted parameters. For the three parameter model, the estimate for X_i in the pair (R_T^i, X_i) is

$$X_i^{\text{e}} = F_{\text{bound}} + \frac{q_2 F_{\text{bound}}}{1 - F_{\text{bound}}}, \quad \text{with} \quad F_{\text{bound}} = \frac{R_T^i}{q_1(\alpha\beta[A]_0 - R_T^i) + R_T^i}. \tag{9}$$

For the two parameter model ($q_1 = 1$) the estimate is

$$X_i^{\text{e}} = F_{\text{bound}} + \frac{q_2 F_{\text{bound}}}{1 - F_{\text{bound}}}, \quad \text{with} \quad F_{\text{bound}} = \frac{R_T^i}{\alpha\beta[A]_0}. \tag{10}$$

The upper bound of the predictive range is the X_i value at which the tagged rate reaches saturation ($R_T^i \geq \alpha\beta[A]_0$). Above this value, the inverted functions are undefined or ill-conditioned, and no estimate can be generated with either model. The lower bound of the predictive range is the lowest X_i such that Q_i is higher than the false-positive threshold (see equation (2)).

Uncertainty estimates for X^{e} are generated as described in section 7.2. The error reported is the relative error $(X^{\text{e}} - X)/X$ converted to a percent. The reported deviations between X^{e} and X are a measure of how well the model fits the data. Future work will explore the performance of estimating previously unknown X^{e}. To estimate unknown X^{e} (e.g., from blinded samples), one would first have to run three or two controls to apply equation (9) or (10), respectively. An alternative work flow for equation (10) is to use a previously identified K_d and run a single control at a saturating value for X to approximate $\alpha\beta = R_T/[A]_0$, in addition to measuring R_T^i at the sub-saturating X_i to be estimated. In that case, since $\partial X^{\text{e}}/\partial q_2 = F_{\text{bound}}/(1 - F_{\text{bound}}) < 1$ for $F_{\text{bound}} < 0.5$, uncertainty in the predicted value X^{e} will not be amplified by uncertainty in the K_d value when $R_T \leq \alpha\beta[A]_0/2$ (i.e., below half-maximum).

9 Estimating target concentration with reduced (two-stage) workflow

The following is a method for target concentration prediction that is based on a two parameter model, but sidesteps the need for model fitting by assuming a previously identified K_d and eliminating the need for the other parameter. The workflow is reduced because nanopore data need only be recorded in two sequential stages: 1) known detection reagent with the unknown target amount, followed by 2) the contents of stage (1) plus a known target-to-detection reagent spiked into the chamber. The method works using either the tagged fraction Q or the tagged capture rate R. Denoting Z to represent Q or R, let $Z(X)$ be the value found after recording at unknown X, and $Z(X + Y)$ by the value at ratio $X + Y$, where Y is a known ratio spiked into the chamber. We

introduce the function

$$f(X) = \log\left[(q_2 + 1 + X) - \sqrt{(q_2 + 1 + X)^2 - 4X}\right]$$

Mathematically, it can be shown that on the domain $0 < X < \infty$, the function $f(X)$ satisfies:

$$0 < \exp(f(X)) < 2, \quad f'(X) > 0, \quad \text{and} \quad f''(X) < 0.$$

From these properties of f and the two parameter models (4) and (8), we can write

$$\log\left(\frac{Z(X+Y)}{Z(X)}\right) = f(X+Y) - f(X) \tag{11}$$

and it follows that the measurable quantity $\log[Z(X+Y)/Z(X)]$ is a decreasing function of X. As a result, each observed value for $\log[Z(X+Y)/Z(X)]$ determines a unique value for X (inverse function theorem). The inversion of (11) to produce the estimate X^{e} is accomplished numerically by gradient descent. The K_d value used to compute q_2 (needed in f and therefore in the inversion of (11)) could be generated by taking an average of values estimated from prior nanopore experiments using the same A and B molecule types. In testing the method here, the K_d used is the value estimated from the 2 parameter model fitting, and X^{e} values are generated for the X data also used in the fitting. Simulated data from the Z distributions (Q or R, at X and $X+Y$) are used to generated uncertainty estimates for X^{e}. A key advantage of removing the need for model fitting is that both X and $X+Y$ can be below saturation. Additionally, since the method uses the same two parameter models that are insensitive to K_d uncertainty when Z is at or below half-maximum, the estimates generated by inverting (11) are also insensitive to uncertainty in K_d provided X is below saturation.

10 Detailed results with HIV Ab 447-52D

10.1 Methods

10.1.1 Construction of HIV Ab detection reagents

We used homo pyrimidine bis-peptide nucleic acid (bisPNA) molecules because they bind to their cognate DNA sequence in dsDNA with high affinity and remain stably incorporated under a wide range of conditions (pH, salt concentrations, temperatures, etc., see [2] and references therein). The bisPNA molecule consist of two halves separated by a flexible PEG linker, the first half binds to the cognate site using Watson-Crick base pairing and the second half binds via Hoogsteen face pairing. Two terminal Lysines are included on each end to enhance complex stability with DNA.

A cysteine residue was incorporated into either the flexor region (PNA^0) or at the Hoogsteen face pairing end (PNA^1) to provide a chemical handle for covalent coupling of the maleimide-containing peptide to the PNA prior to hybridizing with dsDNA. The two bisPNA designs examined had the following sequence and structure (N-term left, to C-term right):

PNA^0: KK-O-TCCCCTCCTTTT-O-Cys-O-TTTTJJTJJJJT-O-KK

PNA^1: KK-O-TCCCCTCCTTTT-OOO-TTTTJJTJJJJT-O-KK-OO-Cys

where K is the amino acid lysine, O represents a 2-aminoethoxy-2-ethoxy acetic acid (AEEA) linker, and Cys is a cysteine. To form bisPNA-peptide, the PNA is first incubated with TCEP to reduce any intermolecular disulfide bonds, and then reacted with a 10 to 50-fold excess of peptide. The x-end terminated peptide sequence, x-KSIHIGPGRAFYTT, are amino acids 305-320 within the third variable loop of the HIV-1 envelope glycoprotein gp120 [13, ?]. The PNA^0-peptide used a PEG terminated peptide sequence, PEG_5-KSIHIGPGRAFYTT, creating the two PNA strands that flanked the peptide strand within the middle. The PNA^1-peptide used Ttds-KSIHIGPGRAFYTT (PNABio), with the peptide at the C-terminal end of the PNA. Nanopore and electrophoretic mobility shift assays (EMSAs) were performed to assess wether the middle-bound peptide within a DNA/PNA^0-peptide was more or less available for HIV Ab binding than the end-bound peptide within DNA/PNA^1-peptide, as described below.

Following the PNA and peptide reaction, the PNA-peptide conjugate is purified by reverse phase HPLC and confirmed by electrospray ionization mass spectrometry (PNABio). In order to form a dsDNA/PNA-peptide complex, the purified bisPNA-peptide conjugate was allowed to incubate with a dsDNA fragments of stated lengths containing only one copy of the complementary sequence to the Watson-Crick strand of the bisPNA, at a 60-fold molar excess for 2 hours at 60C. (10mM sodium phosphate, pH 7.4). Excess bisPNA-peptide was cleaned up by centrifugation in a 100kDa filter (EMD Millipore, UFC570024). Successful invasion of the bisPNA-peptide onto dsDNA was verified via EMSA, as detailed below.

10.1.2 HIV Ab capture ELISA

Rows of Nunc MaxiSorp 96-well plates (ThermoFisher, Cat. No. 442404) were coated with PNA^1-peptide or V3 peptide alone (100ng/well) or BSA (500ng/well), overnight at 4C. Subsequent to coating, the plates were blocked with 1% BSA in PBS for 2 hours at room temperature. The plates were then washed with PBS. Semi-log dilutions of primary antibodies 447-52D or HGD65 (100 uL/well) were incubated for 2 hours at room temperature in antibody diluent (PBS containing

0.2% BSA). Plates were washed 3x with PBS. Horseradish peroxidase-conjugated goat anti-human secondary antibodies (Jackson Immuno Research, 1:5000) were incubated for 30 minutes at room temperature in antibody diluent (PBS containing 0.2% BSA). Plates were washed 3x with PBS. For plate visualization, 100uL TMB chromagen/substrate was applied for a period of 30 minutes (ThermoFisher, Cat. No. SB01). The enzymatic reaction was stopped using 100uL of stop solution (ThermoFisher, Cat. No. SS03100), and the plates were read at 450nm in a Victor2 1420 microplate reader. The resulting data was analyzed using GraphPad Prism 7 Software.

10.1.3 Nanopore experiment incubation conditions

In experiments with buffer only, HIV antibody and detection reagent were mixed at molar ratios ranging from 1 to 20:1 (antibody:detection reagent), and then diluted into recording buffer (1M LiCl,10mM Tris-HCL, pH 8.8, 1mM EDTA) to the reported concentrations for nanopore experiments. During incubation, the concentrations were 20 nM for 1074 bp and 3250 bp DNA scaffolds. The incubation period between detection reagent and Ab was nominally set to 15 minutes, but was reduced in some experiments down to a few seconds (<10 s). In experiments with serum, negative controls were incubated in 20% sample without Ab for 5 min, while for positive controls scaffold/fusion was incubated in 20% sample spiked with Ab for 15 min or 5 min (where stated), prior to dilution into 1M LiCl to the stated concentrations. Saliva samples were not tested with 447-52D.

10.2 Pores P1-P3: Standard holders and bench top amplifiers

Initial experiments tested scaffold and scaffold/fusion without and with HIV Ab 447-52D using standard nanopore chip holders and the 700B amplifier. The results of experiments on three separate pores (P1-P3) are tabulated in Table S3 and plotted in Figure S7. Fresh reagents were prepared for each experiment. Reagents were also tested biochemically using EMSA assays, to ensure sufficiently high quality and yield, prior to measurement on the nanopore. Between every pair of reagents, the channels were flushed with at least 200μL buffer then measured for 5 minutes at the same voltage (100 mV), producing less than 2 events/minute to ensure a clear channel for the next reagent.

The data shows that a deeper and longer event profile is detected only in the presence of full complex (scaffold/fusion/target). Negative controls and positive controls were used to test for positive detection with 99% confidence above 2% false positive, using the procedure presented in Section 6. Specifically, for pore P1, the first three reagents sets (#1-3) collectively were used as the negative controls to identify a maximum δG tagging threshold of 3 nS, with less than 2% of events exceeding this threshold. Subsequently, the test in (2) was applied with $Q_{\text{f.p.}} = 0.02$ for reagents #4-7, with only the positive controls (#4 and #6) resulting in positive results with 99% confidence, and in less than 2 minutes in both cases (TTP, Table S3). Similarly, pores P2 and P3 used the initial three negative control reagents to identify a maximum δG tagging threshold of 5 nS, and only positive controls generated positive results with 99% confidence. Consistent with other nanopore studies [14], a larger diameter nanopore (P1 vs. P2,P3) produces shallower events, resulting in a lower max δG tagging threshold for the larger pore.

We also tested varying the location of the peptide on the PNA. As shown in Table S3 and Figure S7, the scaffold/fusion reagent with the peptide located in the middle of the bisPNA (PNA^0, Tab. S3) generated a modestly lower tagged percentage than for the scaffold/fusion reagent with the peptide located on the end of the bisPNA (PNA^1, Tab. S3). Band intensities from EMSA assays for these two complex types (full complex with PNA^0 and PNA^1) are similar (Fig. S8), though a slightly darker band for full complex with PNA^1 and 10X Ab (#5, P3) is observed compared to full complex with PNA^0 and 10X Ab (#6, P3). All subsequent experiments with HIV Ab exclusively used the scaffold/fusion reagent with the peptide located on the end of the bisPNA (PNA^1).

Table S3: **Traditional holder and amplifier data for HIV Ab 447-52D detection**

Pore ID, Diam. & Setup	Reagents & Concentrations*	Ratio[†] X	Tagged % [‡] (99% CI)	No. Events	Rec. Time	TTP 99%[♮]
P1	1. DNA^a 0.2 nM	–	0.6 ± 1.1%	332	16 min	np
27-28 nm	2. DNA^a/PNA^0-pep 0.2 nM	0	0.2 ± 0.6%	434	13 min	np
Holder	3. DNA^a/PNA^0 0.2 nM + Ab 4 nM	–	1.1 ± 1.5%	353	18 min	np
700B	4. DNA^a/PNA^0-pep 0.2 nM + Ab 3.6 nM	18	23.1 ± 5.2%	441	19 min	1.35 min
	5. DNA^a/PNA^0-pep 0.2 nM	0	2.5 ± 2.4%	285	17 min	np
	6. DNA^a/PNA^0-pep 0.2 nM + Ab 4 nM	20	27.2 ± 5.4%	452	22 min	1.83 min
	7. DNA^a/PNA^0 0.2 nM + Ab 4 nM	–	1.2 ± 1.2%	486	20 min	np
P2	1. DNA^a 0.2 nM	–	0.26 ± 0.7 %	384	24 min	np
18-21 nm	2. DNA^a/PNA^0-pep 0.2 nM	0	0.8 ± 1.9 %	132	21 min	np
Holder	3. DNA^a/PNA^1-pep 0.2 nM	0	0.5 ± 1.1 %	220	23 min	np
700B	4. DNA^a/PNA^1 0.2 nM	–	0 ± 0 %	329	14 min	np
	5. DNA^a/PNA^0-pep 0.2 nM + Ab 2 nM	10	25.6 ± 9.0 %	156	22 min	1.52 min
	6. DNA^a/PNA^1-pep 0.2 nM + Ab 2 nM	10	32.1 ± 7.2 %	277	37 min	6.3 min
	7. DNA^a/PNA^0-pep 0.2 nM + Ab 0.2 nM	1	6.6 ± 4.2 %	228	20 min	10.2 min
P3	1. DNA^a 0.2 nM	–	0 ± 0 %	532	20 min	np
18-21 nm	2. DNA^a/PNA^0-pep 0.2 nM	0	0 ± 0 %	428	22 min	np
Holder	3. DNA^a/PNA^1-pep 0.2 nM	0	1.3 ± 1.7 %	311	32 min	np
700B	4. DNA^a/PNA^1 0.2 nM	–	0.2 ± 0.4 %	581	23 min	np
	5. DNA^a/PNA^1-pep 0.2 nM + Ab 2 nM	10	43.6 ± 7.3 %	310	43 min	3.57 min
	6. DNA^a/PNA^0-pep 0.2 nM + Ab 2 nM	10	13.0 ± 4.5 %	363	17 min	3.7 min
	7. DNA^a/PNA^1-pep 0.2 nM + Ab 0.2 nM	1	10.6 ± 3.0 %	720	24 min	2.22 min

[*] Experiments were conducted at buffer pH 8.8, 1M LiCl, 10 mM Tris, 1 mM EDTA, and 100 mV.

[†] X is the dimensionless target to scaffold/fusion concentration ratio, defined only when the complete detection reagent (scaffold/fusion) competent to bind target is present.

[‡] Events are tagged positive if: max $\delta G > 3$ nS for P1; max $\delta G > 5$ nS for P2, P3. The reported % are $Q \pm Q_{\text{conf}}$ as defined in Section 6.

[♮] TTP (time-to-positive) is the first time at which the tagged percentage minus the 99% CI is greater than, and remains greater than, a false-positive threshold of 2%, as detailed in Section 6. The "np" entry for each reagent set reflects when this criteria is not satisfied.

[a-b] DNA^a = 1074 bp, DNA^b = 3250 bp.

[0-2] PNA^0-pep = bisPNA with middle peptide, PNA^1-pep = bisPNA with end peptide.

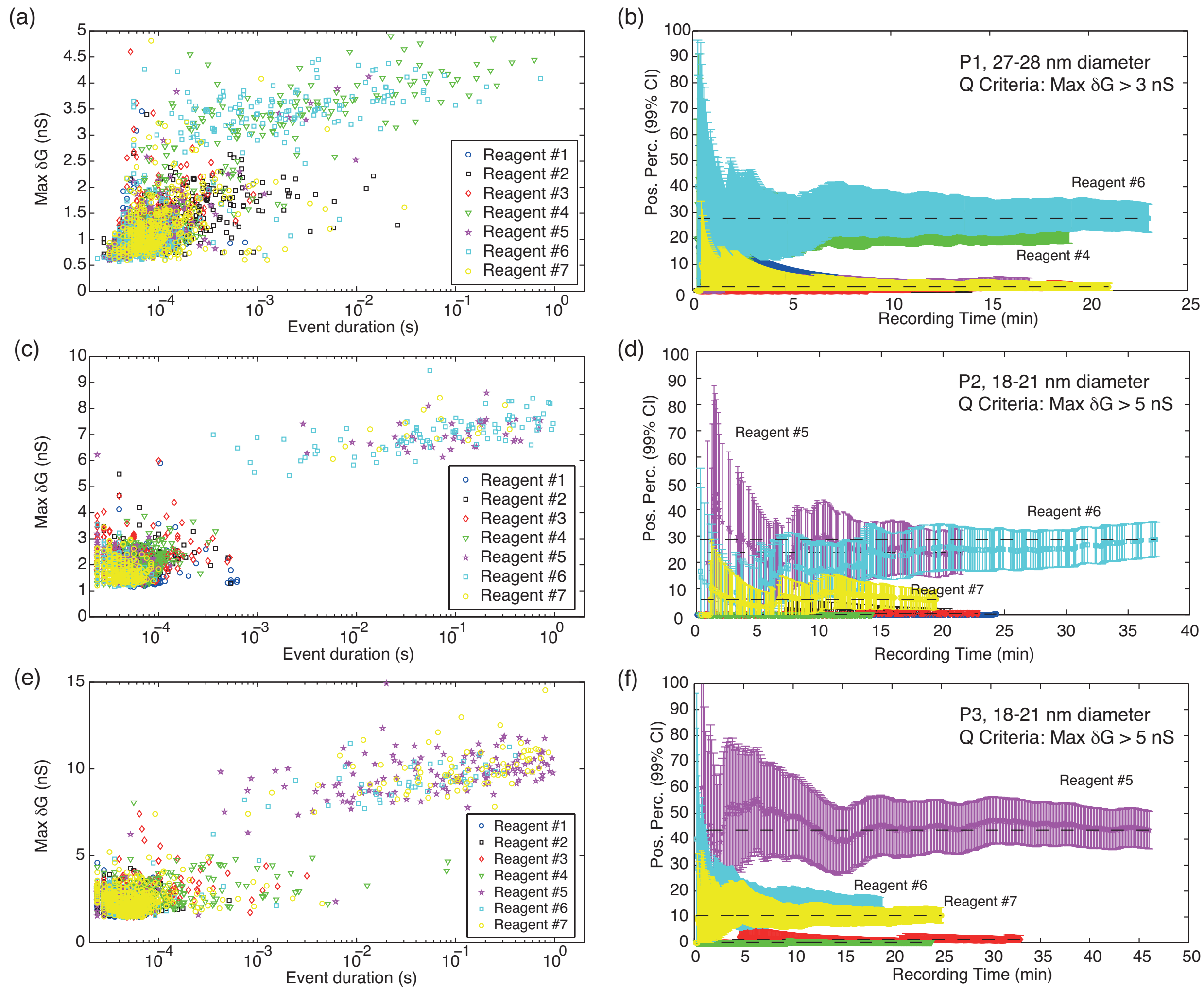

Figure S7: All-event scatter plots of max δG versus duration are shown for three separate experiments using pores (a) P1, (c) P2 and (e) P3, with enumerated reagents identified in Table S3. Applying the tagging criteria identified in Table S3, the resulting tagged (positive) percentages and 99% confidence interval error bars are plotted versus time for each reagent tested on pores (b) P1, (d) P2 and (f) P3. The final tagged percentages and 99% confidence intervals are listed in Table S3.

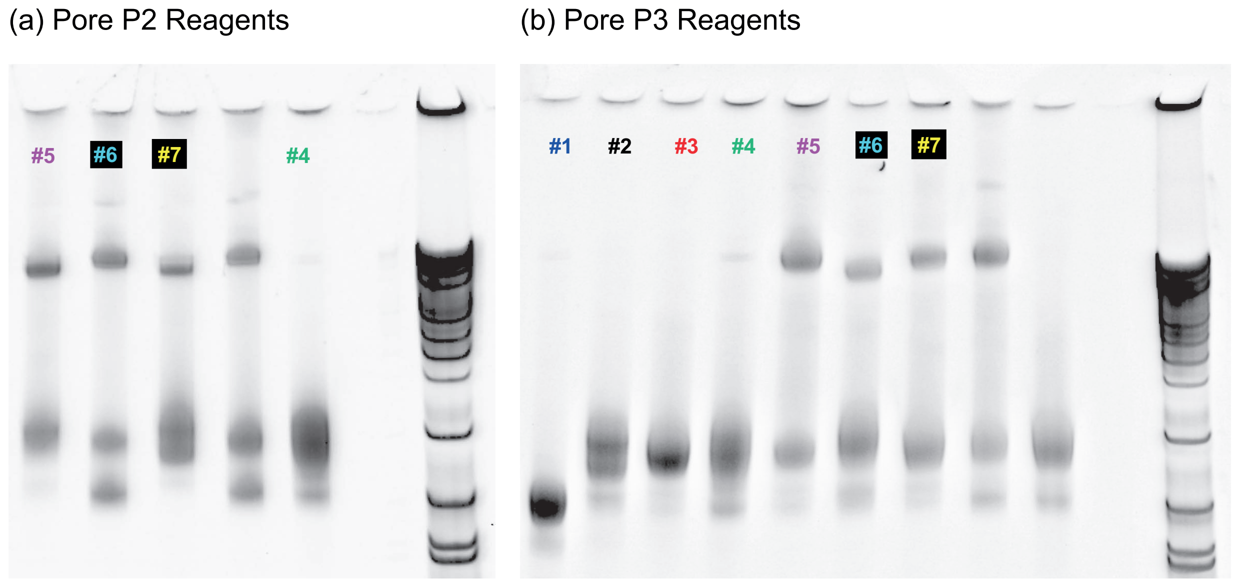

Figure S8: EMSA assays for (a) reagents #4-7 for pore P2 and (b) reagents #1-7 for pore P3, with reagents defined in Table S3.

10.3 Pores P4-P6: Test strips, MOM and serum background

The results of experiments using three different pores (P4-P6) in three separate test strips are tabulated in Table S4 and plotted in Figures S9 and S10. Two of the pores (P5, P6) were tested with an early MOM prototype, and all three pores were tested with positive and negative controls in the presence of varying amounts of serum background. A common tagging threshold combination (max $\delta G > 5$ nS, duration > 0.5 ms) was applied across all three pores, as a display of uniformity. The duration criteria was added to the maximum δG criteria, because serum background events can create events that are as deep as those observed for full complex, but with much shorter duration than is typical for full complex (see Fig. S9 for example).

For nanopores P4 and P5, 1074 bp DNA scaffold was used in the detection reagent as done with pores P1-P3, while pore P6 used 3250 bp scaffold. In the absence of serum background, only the positive controls generated a positive result with 99% confidence above 2% false positive (P4, #2; P6, #3,5). Additionally, this remained true in the presence of varying amounts of diluted serum background. In experiments with serum, the negative controls (P5, #2; P6 #4) and the positive controls (P4, #2-4; P5, #3) were incubated in serum for 5 min before dilution into 1M LiCl to the stated concentrations. Observe that pore P5 was grown to be larger in diameter than P4, resulting in a lower event detection rate for all molecule types. Regardless of the pore size range considered, the tagged fraction is positive only in the presence of the target Ab even in the presence of 1:1000 and 1:333 dilutions of serum background. The sample background, without or with detection reagent, resulted in a deeper tagging threshold to maintain for a 2% false positive, than would be required for detection reagent alone (absent sample). Regardless, only positive controls generated the positively tagged population above that threshold.

At the lowest dilution of serum background considered (1:250) the event rate exceeded 1500 min^{-1}. The increase in event rate due to serum background events becomes more pronounced at lower dilutions, as expected. To increase the relative capture rate of scaffold/fusion, we explored using a longer scaffold [15]. Pore P6 used 3250 bp scaffold instead of the 1074 bp scaffold used for pores P1-P5. The result was an increase in scaffold/fusion capture rate. In the absence of serum background, this had the additional effect of reducing the time-to-99%-positive. Specifically, the time to positive was 7 seconds with 3250 bp scaffold (P6, #5) compared to the fastest time of 22 seconds observed with 1074 bp scaffold (P4, #2). Additionally, the detection reagent was incubated in a tube with the target HIV Ab 447-52D for less than 10 seconds prior to pipetting into the test strip and measuring with the handheld device (Fig. S10).

Table S4: **Test strip data for HIV Ab 447-52D detection in presence of serum background, using bench top (700B) and handheld (MOM V0) amplifiers.**

Pore ID, Diam. & Setup	Reagents & Concentrations	Ratio[†] X	Tagged % [‡] (99% CI)	No. Events	Rec. Time	TTP 99% [♮]
P4	1. DNA^a 0.2 nM	–	0 ± 0 %	159	15 min	np
27-28 nm	2. DNA^a/PNA-pep 0.2 nM + Ab 2 nM	10	61.4 ± 6.2 %	404	15 min	0.3 min
Test strip	3. DNA^a/PNA-pep 0.2 nM + Ab 2 nM					
700B	+ 1:1000 serum dilution	10	8.6 ± 1.2 %	3542	17 min	0.65 min
	4. DNA^a/PNA-pep 0.2 nM + Ab 2 nM					
	+ 1:333 serum dilution	10	3.0 ± 0.49 %	8229	15 min	3.0 min
P5	1. DNA^a 0.3 nM	–	0 ± 0 %	245	9.4 min	np
28-38 nm	2. DNA^a/PNA-pep 0.3 nM					
Test strip	+ 1:1000 serum dilution	0	0.9 ± 1.6 %	221	12.5 min	np
MOM V0	3. DNA^a/PNA-pep 0.3 nM + Ab3 nM					
	+ 1:1000 serum dilution	10	36.9 ± 13.6 %	84	12.7 min	2.9 min
P6	1. DNA^b 0.2 nM	–	0 ± 0 %	747	9.9 min	np
27-29 nm	2. DNA^b/PNA-pep 0.2 nM	0	0.3 ± 0.8 %	309	9.9 min	np
Test strip	3. DNA^b/PNA-pep 0.2 nM + Ab 2 nM	10	11.8 ± 4.0 %	425	9.8 min	1.9 min
MOM V0	4. DNA^b/PNA-pep 0.2 nM					
	+ 1:250 serum dilution	0	0.3 ± 0.2 %	8025	4.8 min	np
	5. DNA^b/PNA-pep 0.18 nM					
	+ Ab 2.75 nM (<10 s incubation)	15	23.6 ± 14.3%	637	9.9 min	0.11 min

[†] X is the dimensionless target to scaffold/fusion concentration ratio, defined only when the complete detection reagent (scaffold/fusion) competent to bind target is present.
[‡] Events are tagged positive if max $\delta G > 5$ nS and duration > 0.5 ms.
[♮] Time-to-positive is the first time at which the tagged percentage minus the 99% CI is greater than, and remains greater than, a false-positive threshold of 2%; the "np" entry for each reagent set reflects when this criteria is not satisfied.
[a-b] DNA^a = 1074 bp, DNA^b = 3250 bp. PNA-pep = bisPNA with end peptide.

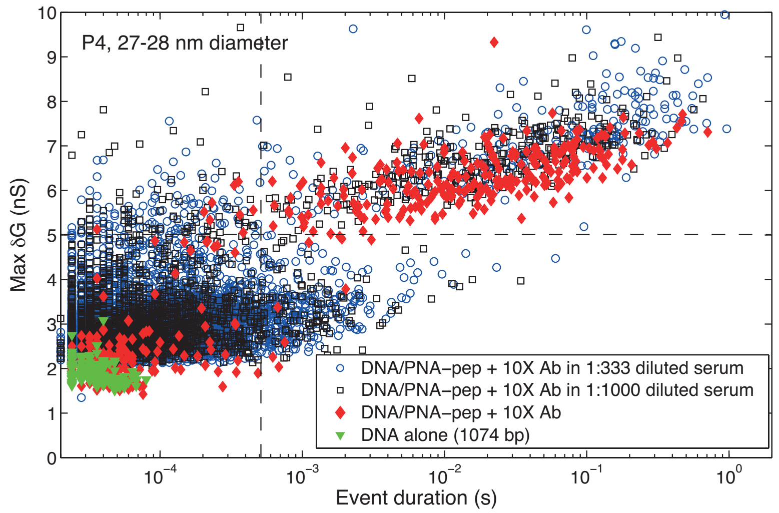

Figure S9: All-event scatter plots of max δG versus duration are shown for pore P4, with numerical values reported in Table S4. The upper right quadrant of the intersecting dashed lines is the tagging domain on the scatter plot, namely (max δG, duration) $>$ (5 nS, 0.5 ms).

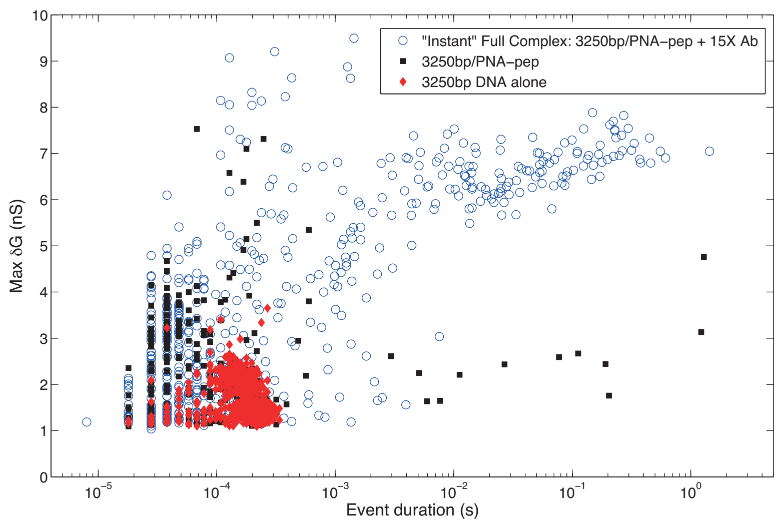

Figure S10: Pore P6 all-event scatter plots of max δG versus duration are shown for three of the reagents (buffer only), with numerical values reported in Table S4. The "instant" full complex corresponds to the final reagent #5, for which incubation between Ab and scaffold/fusion was less than 10 s in a tube, prior to addition to the test strip channel and prompt measurement with the handheld reader.

11 Detailed results with HIV Ab HGD65

11.1 Methods

Detection reagent preparation was performed as described in section 10.1.1 utilizing bisPNA[1]-peptide exclusively as the bi-functional fusion molecule. In experiments with buffer only, HIV antibody and detection reagent were mixed at molar ratios ranging from 0.1:1 to 20:1 (antibody:detection reagent), and then diluted into recording buffer (1M LiCl,10mM Tris-HCL, pH 8.8, 1mM EDTA) to the reported concentrations for nanopore experiments. During incubation, the concentrations were 20 nM for 1074 bp scaffold and 50 nM for 217 bp and 108 bp DNA scaffolds. The incubation period between detection reagent and Ab was nominally set to 15 minutes, but was reduced in some experiments down to 30 seconds. In experiments with serum background, negative controls were incubated in 10% sample without Ab for 5 min, while for positive controls scaffold/fusion was incubated in 10% sample spiked with Ab for 15 min (or less where stated), prior to dilution into 1M LiCl to the stated concentrations.

In experiments with saliva background, HIV antibody and detection reagent were mixed at molar ratios ranging from 0.2, 0.5, 1, 2, 5, and 10:1 (antibody:detection reagent) in 100μL of 1M LiCl, 10mM Tris-HCL (pH 8.8), 1mM EDTA, and 10% donor saliva. Detection reagent (217 bp DNA/PNA-peptide) was held constant at 13.3 nM. Samples were incubated on the bench top at room temperature. Incubation periods were either 5 minutes or 30 seconds. Immediately before data collection, 100μL samples were diluted 10X to 1mL with 1M LiCl, 10mM Tris-HCL, and 1mM EDTA, maintaining antibody:reagent molar ratios indicated above with final concentrations of detection reagent and saliva in all samples being 1.33 nM and 1%, respectively. Saliva was collected from an HIV-negative donor individual using a 1mL plastic transfer pipette (Kinglake #10751) and stored in a 1.5mL Eppendorf tube on ice. Before use, saliva was warmed to room temperature and vortexed to resuspend settled particles.

11.2 Pores P7-P10: Standard holders and bench top amplifiers

Initial experiments tested scaffold and scaffold/fusion without and with HIV Ab HGD65 using standard nanopore chip holders and the 700B amplifier. The results of experiments on four separate pores (P7-P10) are tabulated in Table S5 and plotted in Figures S11-S14. Fresh reagents were prepared for each experiment. Reagents were also tested biochemically using EMSA assays, to ensure sufficiently high quality and yield, prior to measurement on the nanopore (Figs. S12,S13). In nanopore recordings, between every pair of reagents, the channels were flushed with at least 200μL buffer then measured for 5 minutes at the same voltage (100 mV), producing less than 2 events/minute to ensure a clear channel for the next reagent.

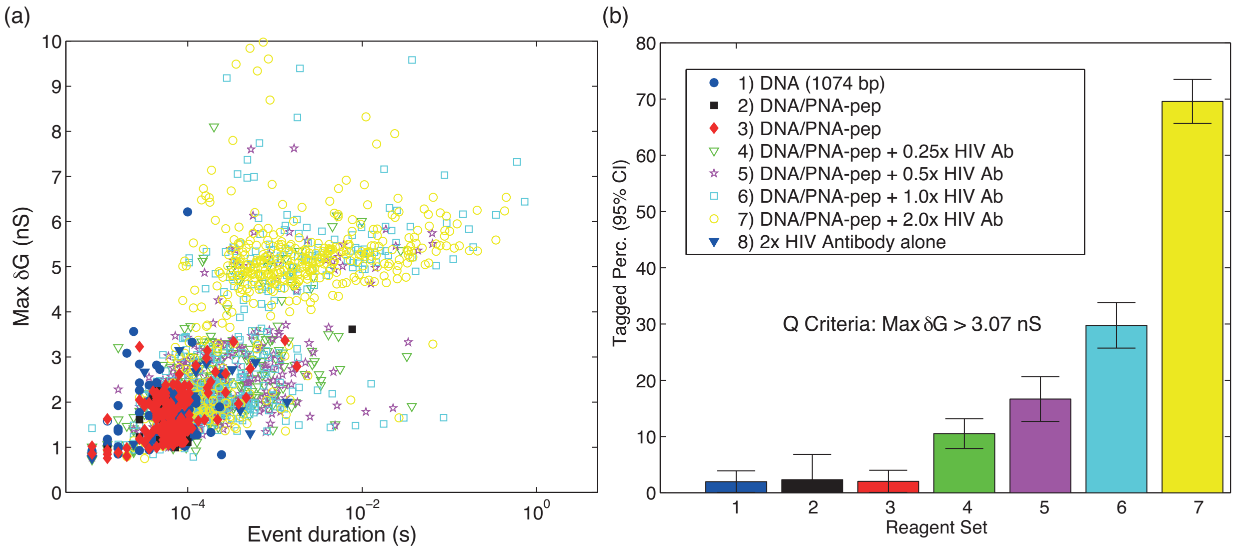

Figure S11: Pore P7 experiment with titration of HIV Ab HGD65. (a) All-event scatter plots of max δG versus duration are shown, with numerical values reported in Table S5. (b) The corresponding tagged percentage ($Q \pm Q_{\text{conf}}$, 95% CI) for each reagent set.

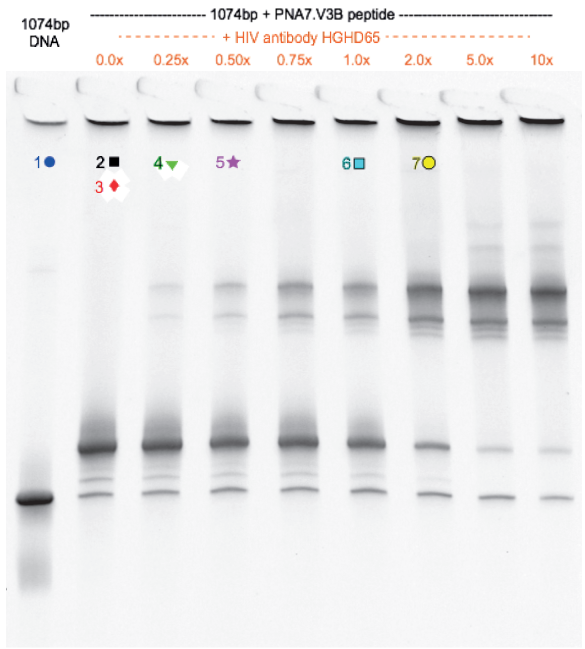

Figure S12: Pore P7 EMSA assays for reagent sets #1-7 defined in Table S5.

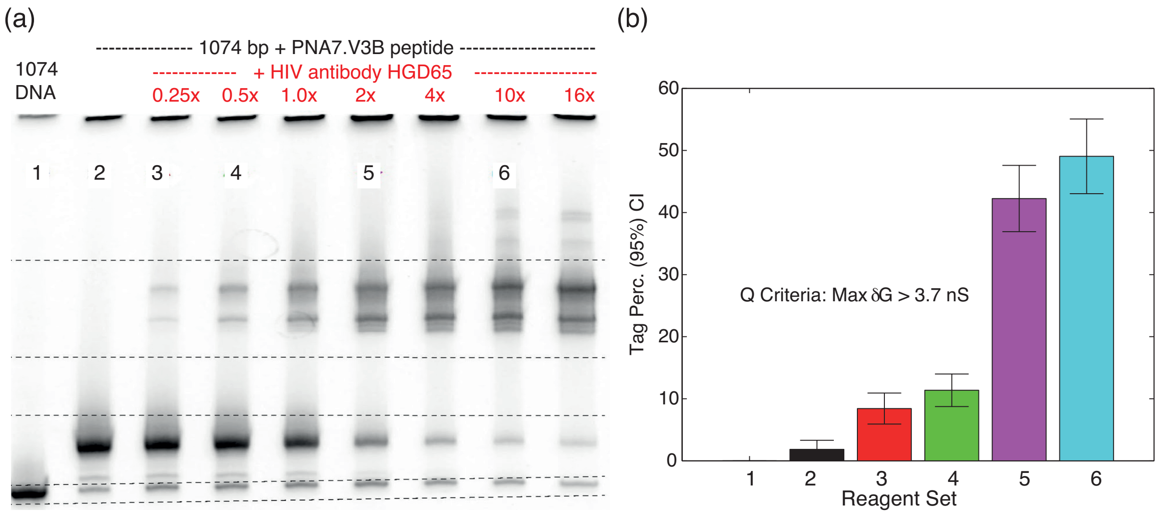

Figure S13: Pore P8 experiment. (a) EMSA assays for reagent sets #1-6 defined in Table S5, and (b) the corresponding tagged percentage ($Q \pm Q_{\mathrm{conf}}$, 95% CI) for each reagent set.

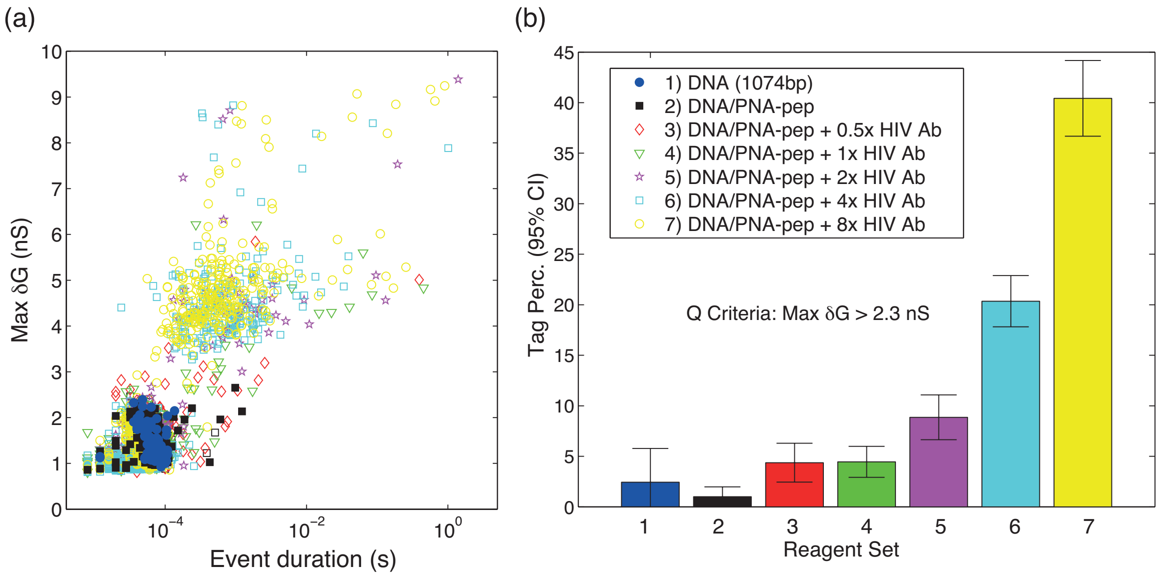

Figure S14: Pore P9 experiment. (a) All-event scatter plots of max δG versus duration with numerical values reported in Table S5, and (b) the corresponding tagged percentage ($Q \pm Q_{\mathrm{conf}}$, 95% CI) for each reagent set.

Table S5: **Traditional holder and amplifier data for HIV Ab HGD65 detection**

Pore ID, Diam. & Setup	Reagents & Concentrations*	Ratio[†] X	Tagged % [‡] (99% CI)	No. Events	Rec. Time	TTP 99%[♮]
P7	1. DNA^a 0.4 nM	–	1.98 ± 2.5%	202	9 min	np
22-24 nm	2. DNA^a/PNA-pep 0.2 nM	0	2.3 ± 5.9%	43	7 min	np
Holder	3. DNA^a/PNA-pep 0.4 nM	0	2.0 ± 2.6%	197	14 min	np
700B	4. DNA^a/PNA-pep 0.4 nM + Ab 0.1 nM	0.25	10.5 ± 3.5%	513	30 min	3.35 min
	5. DNA^a/PNA-pep 0.4 nM + Ab 0.2 nM	0.5	16.7 ± 5.2%	336	14 min	2.38 min
	6. DNA^a/PNA-pep 0.4 nM + Ab 0.4 nM	1	29.8 ± 5.3%	494	18 min	0.74 min
	7. DNA^a/PNA-pep 0.4 nM + Ab 0.8 nM	2	69.6 ± 5.2%	529	21 min	0.51 min
	8. Ab 0.4 nM	–	5.6 ± 13.9%	18	5 min	np
P8	1. DNA^a 0.3 nM	–	0 ± 0 %	554	11 min	np
28-38 nm	2. DNA^a/PNA-pep 0.4 nM	0	1.8 ± 1.9 %	325	9 min	np
Holder	3. DNA^a/PNA-pep 0.4 nM + Ab 0.1 nM	0.25	8.4 ± 3.3 %	475	9 min	2.04 min
700B	4. DNA^a/PNA 0.4 nM + Ab 0.2 nM	0.5	11.4 ± 3.5 %	563	11 min	1.26 min
	5. DNA^a/PNA-pep 0.4 nM + Ab 0.8 nM	2	42.3 ± 7.0 %	329	12 min	0.125 min
	6. DNA^a/PNA-pep 0.4 nM + Ab 4 nM	10	49.1 ± 7.9 %	265	10 min	0.288 min
P9	1. DNA^a 1 nM	–	2.4 ± 4.4 %	82	7 min	np
26-29 nm	2. DNA^a/PNA-pep 1 nM	0	1.0 ± 1.3 %	399	10 min	np
Holder	3. DNA^a/PNA-pep 1 nM + Ab 0.5 nM	0.5	4.4 ± 2.5 %	435	10 min	8.95 min
700B	4. DNA^a/PNA 1 nM + Ab 1 nM	1	4.5 ± 2.0 %	696	11 min	4.63 min
	5. DNA^a/PNA-pep 1 nM + Ab 2 nM	2	8.9 ± 2.9 %	632	10 min	0.88 min
	6. DNA^a/PNA-pep 1 nM + Ab 4 nM	4	20.4 ± 3.3 %	968	10 min	0.146 min
	7. DNA^a/PNA-pep 1 nM + Ab 8 nM	8	40.4 ± 4.9 %	658	11 min	2.4 sec
P10	1. DNA^a/PNA-pep 0.5 nM + Ab 0.5 nM	1	11.5 ± 2.2 %	1428	18 min	0.826 min
27-30 nm	2. DNA^a/PNA-pep 0.5 nM + Ab 1.0 nM	2	20.6 ± 6.3 %	272	9 min	4.43 min**
Holder	3. DNA^a/PNA-pep 0.5 nM + Ab 2.5 nM	5	49.9 ± 5.2 %	623	14 min	0.159 min
700B	4. DNA^a/PNA 0.5 nM + Ab 5 nM	10	70.1 ± 4.3 %	748	9 min	0.223 min
	5. DNA^a/PNA-pep 0.5 nM + Ab 7.5 nM	15	63.6 ± 4.5 %	747	9 min	0.0606 sec
	6. DNA^a/PNA-pep 0.5 nM	0	2.1 ± 1.3 %	847	6 min	np

[*] Experiments were conducted at buffer pH 8.8, 1M LiCl, 10 mM Tris, 1 mM EDTA, and 100 mV.

[†] X is the dimensionless target to scaffold/fusion concentration ratio, defined only when the complete detection reagent (scaffold/fusion) competent to bind target is present.

[‡] Events are tagged positive if: max $\delta G > 3.1$ nS for P7; max $\delta G > 3.7$ nS for P8; max $\delta G > 2.3$ nS for P9; max $\delta G > 2.5$ nS for P10. The reported % are $Q \pm Q_{\text{conf}}$ as defined in Section 6.

[♮] TTP (time-to-positive) is the first time at which the tagged percentage minus the 99% CI is greater than, and remains greater than, a false-positive threshold of 2%, as detailed in Section 6. The "np" entry for each reagent set reflects when this criteria is not satisfied.

[a-c] DNA^a = 1074 bp, DNA^b = 217 bp, DNA^c = 108 bp.

[**] Significant clogging during first 4 minutes of recording requiring auto-zapping and auto-trimming, thus delaying TTP. Subsequent reagents required no zapping as the pore resumed normal function.

The tagged fraction model fitting performance for P7-P10 are reported in Table S6. P7 and P9 data do not saturate in Q value, which is unexpected in the case of P9 given there are X values well above 1. In these sub-saturating cases, the model fitting produces parameters with high coefficient of variation (CV, standard deviation divided by mean), since without both transition to saturation and saturation data points the models are effectively over parameterized. On the other hand, when sub-saturation and saturation data are recorded as with P8 and P10, the parameter estimates have smaller CVs and thus their values are more meaningful. The model-based target concentration predictions are reported in Table S7.

Table S6: **Fitted parameter values from modeling tagged fraction**

Pore	3 parameter model equation (3)				2 parameter model equation (4)		
ID	α	K_d (nM)	q_1	NRMS	α	K_d (nM)	NRMS
P7	19 ± 4.8	0.62 ± 1.1	0.04 ± 0.11	3.5 %	34 ± 43	39 ± 51	3.8 %
P8	0.51 ± 0.037	0.046 ± 0.048	0.47 ± 0.12	3.8 %	0.52 ± 0.04	0.13 ± 0.071	8 %
P9	40 ± 11	270 ± 620	0.35 ± 0.76	2.9 %	8.4 ± 8.7	160 ± 170	3.7 %
P10	0.83 ± 0.03	0.11 ± 0.033	0.076 ± 0.02	7.3 %	0.75 ± 0.038	0.93 ± 0.25	15 %

* Parameters and normalized root-mean-square error (NRMS) are defined in section 7.1.

Table S7: **Target concentration estimates using tagged fraction models**

Pore	Ratio	$[B]_0$	3 parameter model equation (5)			2 parameter model equation (6)		
ID	X	(nM)	$[B]_0^e$ (nM)	CV	PE	$[B]_0^e$ (nM)	CV	PE
P7	0.25	0.1	0.14 ± 0.021	15 %	37 %	0.12 ± 0.013	11 %	21 %
	0.5	0.2	0.22 ± 0.028	13 %	7.7 %	0.19 ± 0.029	15 %	-2.6 %
	1	0.4	0.37 ± 0.028	7.6 %	-7.5 %	0.35 ± 0.025	7.3 %	-13 %
	2	0.8	0.81 ± 0.03	3.7 %	0.85 %	0.82 ± 0.032	3.9 %	2.8 %
P8	0.25	0.1	0.14 ± 0.025	18 %	39 %	0.094 ± 0.017	18 %	-6.4 %
	0.5	0.2	0.18 ± 0.026	14 %	-11 %	0.13 ± 0.02	16 %	-37 %
	2	0.8	0.82 ± 3.2	4e+02 %	2.3 %	0.92 ± NaN♯	–	15 %
	10	4	3.7 ± NaN♯	–	-7.9 %	3.6 ± NaN♯	–	-10 %
P9	0.5	0.5	0.86 ± 0.22	26 %	72 %	0.84 ± 0.17	21 %	68 %
	1	1	0.88 ± 0.2	22 %	-12 %	0.86 ± 0.15	18 %	-14 %
	2	2	1.8 ± 0.25	14 %	-12 %	1.7 ± 0.24	14 %	-14 %
	4	4	4 ± 0.3	7.4 %	0.8 %	4 ± 0.31	7.8 %	-0.17 %
	8	8	8 ± 0.58	7.3 %	0.62 %	8.1 ± 0.62	7.6 %	1.6 %
P10	1	0.5	0.57 ± 0.047	8.3 %	14 %	0.24 ± 0.038	16 %	-52 %
	2	1	0.86 ± 0.095	11 %	-14 %	0.47 ± 0.095	20 %	-53 %
	5	2.5	2.6 ± 0.24	9.5 %	2.6 %	2.1 ± 0.31	15 %	-16 %
	10	5	7.9 ± 1.9	24 %	59 %	12 ± NaN♯	–	1.4e+02 %
	15	7.5	5.1 ± 0.71	14 %	-31 %	5.5 ± 2.2	40 %	-27 %

* CV (coefficient of variation) is the standard deviation divided by the mean for $[B]_0^e$ converted to a percentage, and PE (percentage error) = $100 \times ([B]_0^e - [B]_0)/[B]_0$.

♯ "NaN" is reported as the uncertainty estimate when the simulated Q data exceeds the simulated α value, in which case inversion of the model function is undefined.

In Table S7, uncertainty estimates for $[B]_0^e$ report "NaN" when the simulated Q data exceed the simulated α value, since above saturation values the inversion of the model function is undefined. Table S7 shows that the predictive performance of target concentration is good for X values below saturation. At or above saturation, the predictions are unreliable as expected (P8, $X = 2, 10$; P10, $X = 10, 15$). The three and two parameter models performed comparably for data sets entirely below saturation (P7, P9), while the three parameter model outperformed the two parameter model for data sets with sub-saturating and saturating data (P8, P10), in which cases only sub-saturating $[B]_0^e$ values are meaningful. The model fitting is plotted with data in Figure S15. In the remaining experimental results for all pores, the model predicted $[B]_0^e$ value is reported only if a finite uncertainty estimate could be computed, and if the corresponding CV is less than 100%.

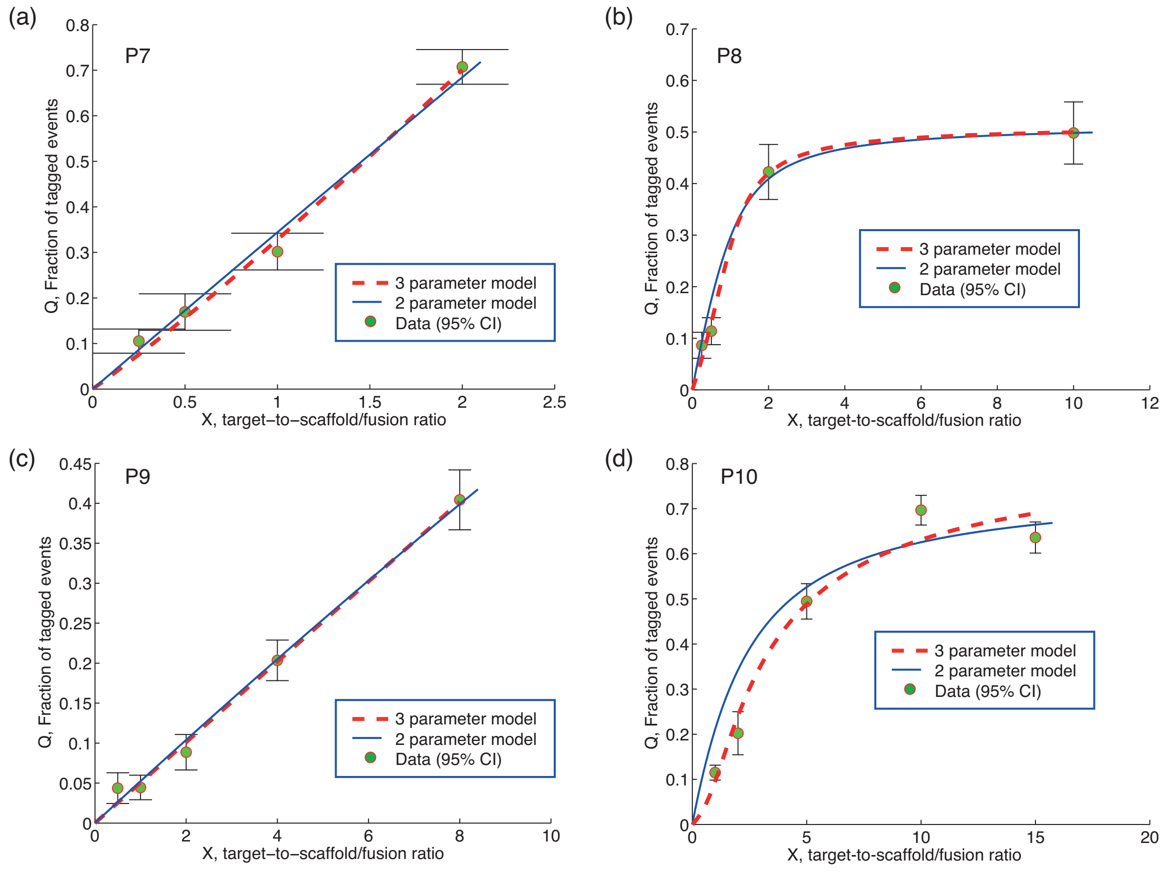

Figure S15: Modeling of tagged fraction Q vs. target to scaffold/fusion concentration ratio X for pore experiments (a) P7 (b) P8 (c) P9 and (d) P10. The data is the tagged fraction ($Q \pm Q_{\mathrm{conf}}$, 95% CI) for each reagent set. The three and two-parameter models are equations (3) and (4), respectively.

The tagged capture rates for P7-P10 are reported in Table S8 and the model fitting results are reported in Table S9. The R_T trends over the course of each experiment appear less consistent than the Q trends, in terms of increasing with X in a predictable way. As in the tagged fraction data, P7 and P9 data do not saturate in R_T value. In these sub-saturating cases, the fitted parameters again show a higher CV, compared to data sets with sub-saturation and saturation data (P8, P10). Modeling fitting to the Q and R-based data for P8 and P10 suggests a K_d value between 0.1 and 1 nM.

Table S8: **Tagged capture rate data**

Pore ID	Ratio X	Tagged capture rate R_T	fit R^2
P7	0.25	2.07 ± 0.335 min^{-1}	0.991
	0.5	4.08 ± 0.643 min^{-1}	0.962
	1	8.57 ± 0.833 min^{-1}	0.994
	2	21.3 ± 1.29 min^{-1}	0.997
P8	0.25	3.8 ± 0.706 min^{-1}	0.928
	0.5	5.99 ± 0.891 min^{-1}	0.94
	2	13.4 ± 1.35 min^{-1}	0.991
	10	15.4 ± 1.6 min^{-1}	0.989
P9	0.5	2.17 ± 0.593 min^{-1}	0.901
	1	4.52 ± 0.967 min^{-1}	0.98
	2	5.35 ± 0.851 min^{-1}	0.985
	4	19.1 ± 1.62 min^{-1}	0.987
	8	26.7 ± 1.95 min^{-1}	0.994
P10	1	9.81 ± 0.912 min^{-1}	0.995
	2	10.9 ± 1.75 min^{-1}	0.995
	5	28.9 ± 1.96 min^{-1}	0.988
	10	65.6 ± 3.42 min^{-1}	0.997
	15	65.9 ± 3.6 min^{-1}	0.998

* R_T and R^2 calculations are defined in section 8.1.

Table S9: **Fitted parameter values from modeling tagged capture rate**

Pore	3 parameter model equation (7)				2 parameter model equation (8)		
ID	$\alpha\beta$ (nM sec)$^{-1}$	K_d (nM)	q_1	NRMS	$\alpha\beta$ (nM sec)$^{-1}$	K_d (nM)	NRMS
P7	16 ± 8.8	0.25 ± 0.24	0.027 ± 0.06	1.3 %	39 ± 25	41 ± 29	2.9 %
P8	0.66 ± 0.079	0.12 ± 6.1	1.2 ± 16	1.9 %	0.68 ± 0.069	0.11 ± 0.079	6.2 %
P9	0.85 ± 1.6	0.32 ± 12	0.052 ± 0.048	7.3 %	1.2 ± 0.96	13 ± 15	10 %
P10	4.2 ± 0.59	0.35 ± 0.21	0.062 ± 0.028	9.8 %	3.8 ± 0.85	4.8 ± 2.3	12 %

* Parameters and normalized root-mean-square error (NRMS) are defined in section 8.1.

The model-based target concentration predictions are reported in Table S10. The model predicted $[B]_0^e$ value is reported only if a finite uncertainty estimate could be computed, which is possible when the simulated R_T data are below the simulated $\alpha\beta[A]_0$ saturation parameter value, and if the corresponding CV is less than 100%; otherwise, "na" is reported. The table shows that the predictive performance of target concentration is good for X values below saturation. The model fitting is plotted with data in Figure S16.

Table S10: **Target concentration estimates from modeling tagged capture rate**

Pore ID	Ratio X	$[B]_0$ (nM)	3 parameter model equation (5) $[B]_0^e$ (nM)	CV	PE	2 parameter model equation (6) $[B]_0^e$ (nM)	CV	PE
P7	0.25	0.1	0.12 ± 0.024	21 %	15 %	0.083 ± 0.031	37 %	-17 %
	0.5	0.2	0.21 ± 0.033	16 %	5 %	0.16 ± 0.035	22 %	-18 %
	1	0.4	0.39 ± 0.036	9.3 %	-3 %	0.35 ± 0.058	17 %	-14 %
	2	0.8	0.8 ± 0.082	10 %	0.34 %	0.86 ± 0.048	5.6 %	7.7 %
P8	0.25	0.1	0.11 ± 0.03	27 %	11 %	0.13 ± 0.029	23 %	26 %
	0.5	0.2	0.19 ± 0.043	23 %	-5.7 %	0.21 ± 0.049	23 %	4.9 %
P9	0.5	0.5	0.73 ± 0.19	27 %	47 %	0.42 ± 0.12	28 %	-16 %
	1	1	1.2 ± 0.23	18 %	25 %	0.91 ± 0.24	26 %	-9.5 %
	2	2	1.4 ± 0.21	15 %	-29 %	1.1 ± 0.23	21 %	-46 %
	4	4	4.6 ± 0.67	14 %	15 %	4.7 ± 0.7	15 %	19 %
	8	8	7.7 ± 2	26 %	-3.7 %	na$^\flat$	–	–
P10	1	0.5	0.76 ± 0.079	10 %	52 %	0.49 ± 0.099	20 %	-2.4 %
	2	1	0.83 ± 0.13	15 %	-17 %	0.55 ± 0.13	25 %	-45 %
	5	2.5	2.1 ± 0.19	9.1 %	-17 %	1.7 ± 0.29	17 %	-31 %
	10	5	6.5 ± 0.92	14 %	30 %	6.6 ± 1.4	21 %	32 %
	15	7.5	6.6 ± 0.89	14 %	-13 %	6.7 ± 2.1	31 %	-11 %

* CV (coefficient of variation) is the standard deviation divided by the mean for $[B]_0^e$ converted to a percentage, and PE (percentage error) $= 100 \times ([B]_0^e - [B]_0)/[B]_0$.

$^\flat$ "na" is reported when the estimate has a CV of 100% or greater.

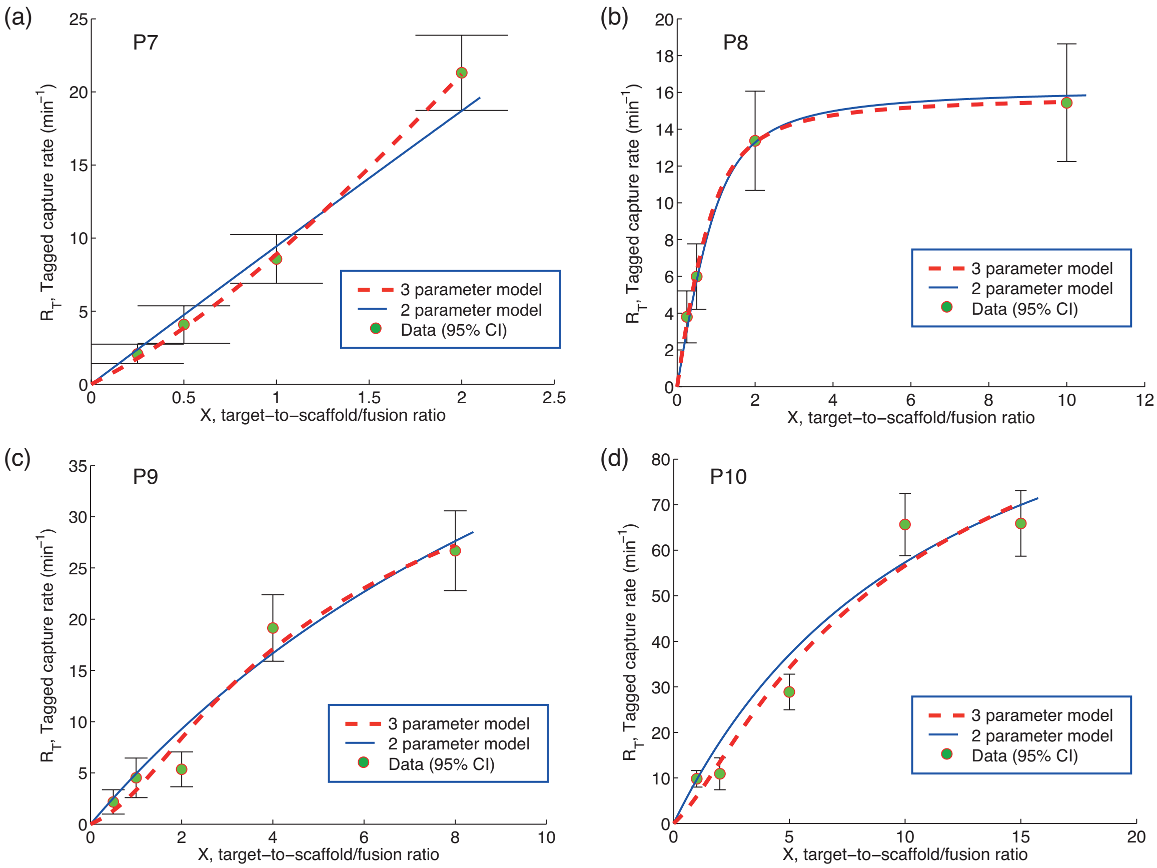

Figure S16: Modeling of tagged capture rate R_T vs. target to scaffold/fusion concentration ratio X for pore experiments (a) P7 (b) P8 (c) P9 and (d) P10. The data is the tagged capture rate (95% CI) for each reagent set. The three and two-parameter models are equations (7) and (8), respectively.

The two-stage workflow results for pores P7-P10 are provided in Table S11. As stated, predicted $[B]_0^e$ values are reported only if a finite uncertainty estimate could be computed and the corresponding CV is less than 100%; otherwise, "na" is reported. In one case (P10) the measured $Q = 70\%$ is higher at $X = 10$ than at $X + Y = 15$ ($Q = 64\%$), and so the inversion can't generate an estimate at all, a consequence of both values being too close to saturation. Generally, the two stage approach performs best when X is sufficiently below saturation, and when using the largest Y available. In some cases, averaging the estimates generated from using Q and R_T data improves the accuracy.

Table S11: **Target concentration estimates from two-stage workflow**

Pore	Ratio	$[B]_0$	Tagged fraction Q			Tagged capture rate R_T		
ID	$X \to X+Y$	(nM)	$[B]_0^e$ (nM)	CV	PE	$[B]_0^e$ (nM)	CV	PE
P7	0.25 → 1	0.1	0.16 ± 0.034	22 %	59 %	0.094 ± 0.025	27 %	-6.2 %
	0.25 → 2	0.1	0.12 ± 0.018	15 %	21 %	0.073 ± 0.014	20 %	-27 %
	0.5 → 1	0.2	0.25 ± 0.11	42 %	26 %	0.18 ± 0.074	41 %	-11 %
	0.5 → 2	0.2	0.19 ± 0.03	16 %	-7.1 %	0.14 ± 0.029	21 %	-31 %
	1 → 2	0.4	0.29 ± 0.037	13 %	-27 %	0.26 ± 0.056	21 %	-35 %
P8	0.25 → 2	0.1	0.089 ± 0.016	18 %	-11 %	0.13 ± 0.031	24 %	27 %
	0.25 → 10	0.1	0.093 ± 0.015	16 %	-6.6 %	0.13 ± 0.033	25 %	31 %
	0.5 → 2	0.2	0.12 ± 0.018	15 %	-42 %	0.21 ± 0.057	27 %	5.1 %
	0.5 → 10	0.2	0.13 ± 0.017	14 %	-37 %	0.22 ± 0.053	24 %	9.8 %
P9	0.5 → 4	0.5	0.93 ± 0.27	29 %	86 %	0.35 ± 0.11	31 %	-29 %
	0.5 → 8	0.5	0.86 ± 0.2	24 %	72 %	0.43 ± 0.13	30 %	-15 %
	1 → 4	1	0.82 ± 0.2	24 %	-18 %	0.74 ± 0.23	31 %	-26 %
	1 → 8	1	0.82 ± 0.17	21 %	-18 %	0.92 ± 0.25	27 %	-8.3 %
	2 → 4	2	1.5 ± 0.41	27 %	-25 %	0.66 ± 0.16	24 %	-67 %
	2 → 8	2	1.6 ± 0.29	18 %	-20 %	1 ± 0.22	22 %	-50 %
	4 → 8	4	3.9 ± 0.65	17 %	-3.7 %	na	–	–
P10	1 → 2	0.5	0.43 ± 0.15	35 %	-15 %	na	–	–
	1 → 5	0.5	0.25 ± 0.024	9.5 %	-50 %	0.69 ± 0.11	17 %	39 %
	1 → 10	0.5	0.21 ± 0.018	8.4 %	-57 %	0.42 ± 0.049	12 %	-16 %
	1 → 15	0.5	0.26 ± 0.022	8.5 %	-49 %	0.52 ± 0.061	12 %	3.2 %
	2 → 5	1	0.46 ± 0.076	17 %	-54 %	0.66 ± 0.18	28 %	-34 %
	2 → 10	1	0.4 ± 0.056	14 %	-60 %	0.44 ± 0.082	19 %	-56 %
	2 → 15	1	0.5 ± 0.074	15 %	-50 %	0.56 ± 0.11	19 %	-44 %
	5 → 10	2.5	1.5 ± 0.17	12 %	-41 %	1.2 ± 0.17	14 %	-53 %
	5 → 15	2.5	2.4 ± 0.41	17 %	-5.1 %	1.7 ± 0.23	13 %	-31 %

* Based on K_d value from 2-parameter model fittings, the method is defined in section 9 with X the unknown and Y the known ratio. CV (coefficient of variation) is the standard deviation divided by the mean for $[B]_0^e$ converted to a percentage; PE (percentage error) = $100 \times ([B]_0^e - [B]_0)/[B]_0$. The "na" is reported when the estimate has a CV of 100% or greater.

11.3 Pores P11-P13: Standard holders and MOM amplifiers

Experiments tested scaffold and scaffold/fusion without and with HIV Ab HGD65 using standard nanopore chip holders and the MOM amplifier. The results of experiments on three separate pores (P11-P13) are tabulated in Table S12. As before, fresh reagents were prepared for each experiment and tested biochemically using EMSA assays.

Table S12: **Traditional holder and MOM data for HIV Ab HGD65 detection**

Pore ID, Diam. & Setup	Reagents & Concentrations*	Ratio† X	Tagged % ‡ (99% CI)	No. Events	Rec. Time	TTP 99%♮
P11	1. DNA^a/PNA-pep 0.5 nM	0	1.72 ± 2.2%	233	4 min	np
26-30 nm	2. DNA^a/PNA-pep 0.5 nM + Ab 1 nM	2	16.3 ± 5.0%	363	11 min	2.08 min
Holder	3. DNA^a/PNA-pep 0.5 nM + Ab 2.5 nM	5	26.5 ± 5.2%	484	11 min	0.309 min
MOM	4. DNA^a/PNA-pep 0.5 nM + Ab 5 nM	10	34.6 ± 3.8%	1068	24 min	4.9 sec
P12	1. DNA^a/PNA-pep 0.5 nM + Ab 0.5 nM	1	14.4 ± 3.7%	591	8 min	0.261 min
31-33 nm	2. DNA^a/PNA-pep 0.5 nM + Ab 1 nM	2	42.6 ± 6.1%	441	8 min	0.523 min
Holder	3. DNA^a/PNA-pep 0.5 nM + Ab 2.5 nM	5	93.9 ± 3.4%	280	10 min	0.253 min
MOM	4. DNA^a/PNA-pep 0.5 nM + Ab 5 nM	10	93.3 ± 2.6%	639	11 min	5.1 sec
	5. DNA^a/PNA-pep 0.5 nM	0	1.9 ± 1.8 %	378	4 min	np
P13	1. DNA^a/PNA-pep 0.5 nM	0	1.9 ± 2.0%	311	3 min	np
24-26 nm	2. DNA^a/PNA-pep 0.5 nM + Ab 0.5 nM	1	6.4 ± 3.1%	420	7 min	2.28 min
Holder	3. DNA^a/PNA-pep 0.5 nM + Ab 1 nM	2	34.3 ± 7.4%	274	12 min	0.563 min
MOM	4. DNA^a/PNA-pep 0.5 nM + Ab 2.5 nM	5	74.6 ± 8.2%	189	10 min	0.16 min
	5. DNA^a/PNA-pep 0.5 nM + Ab 5 nM	10	81.7 ± 6.6 %	230	11 min	0.487 min
	6. DNA^a/PNA-pep 0.5 nM	0	0.8 ± 2.1%	125	4 min	np

* Experiments were conducted at buffer pH 8.8, 1M LiCl, 10 mM Tris, 1 mM EDTA, and 100 mV.

† X is the dimensionless target to scaffold/fusion concentration ratio, defined only when the complete detection reagent (scaffold/fusion) competent to bind target is present.

‡ Events are tagged positive if: max $\delta G > 4.6$ nS for P11; max $\delta G > 3.6$ nS for P12; max $\delta G > 3.5$ nS for P13. The reported % are $Q \pm Q_{\text{conf}}$ as defined in Section 6.

♮ TTP (time-to-positive) is the first time at which the tagged percentage minus the 99% CI is greater than, and remains greater than, a false-positive threshold of 2%, as detailed in Section 6. The "np" entry for each reagent set reflects when this criteria is not satisfied.

$^{a\text{-}c}$ DNA^a = 1074 bp, DNA^b = 217 bp, DNA^c = 108 bp.

** Significant clogging during first 4 minutes of recording requiring auto-zapping and auto-trimming, thus delaying TTP.

The tagged fraction model fitting performance for P11-P13 are reported in Table S13. P11 data does not saturate to the extent observed in the P12 and P13 data. The three parameter model fits the P12 and P13 data better than the two parameter model, particularly during the transition to saturation. For these data sets, α converging to a number closer to one could be due in part to the increased noise when measuring with the MOM compared to the 700B. That is, higher noise could mean that fewer unbound events are detected. Between the three and two parameter models, the K_d predicted appears to be 0.01 and 1 nM, a wider range than predicted for P8 and P10.

Table S13: **Fitted parameter values from modeling tagged fraction**

Pore	3 parameter model equation (3)				2 parameter model equation (4)		
ID	α	K_d (nM)	q_1	NRMS	α	K_d (nM)	NRMS
P11	0.48 ± 0.057	15 ± 11	8.1 ± 7.3	2.5 %	0.47 ± 0.062	1.7 ± 0.66	6.2 %
P12	1.2 ± 0.02	0.024 ± 0.0069	0.033 ± 0.0085	7.7 %	1.1 ± 0.024	0.54 ± 0.08	20 %
P13	1 ± 0.045	0.0079 ± 0.0043	0.009 ± 0.0042	3.6 %	1 ± 0.078	1 ± 0.32	16 %

* Parameters and normalized root-mean-square error (NRMS) are defined in section 7.1.

The model-based target concentration predictions using Q for P11-13 are reported in Table S14. The PE and CV are measures of prediction accuracy and precision, respectively. As aggregate measures of performance across pores, we can compute the average absolute value for PE and the average CV. Absolute value is needed for PE since it can be positive or negative, and large errors can average to zero. The three parameter model estimates for pores P11-13 had average $|\text{PE}| = 12\%$ and CV = 15%, significantly better than the averages $|\text{PE}| = 35\%$ and CV = 18% for the two parameter model. By contrast, the performance for both models was comparable for pores P7-10, with means $|\text{PE}| = 21\%$ and $|\text{PE}| = 22\%$ and means CV = 14% and CV = 16% for the three and two parameter models, respectively. The best performance is for P12-13 and sub-saturating concentrations ($X \leq 2$), producing averages $|\text{PE}| = 5\%$ and CV = 6% using the three parameter model.

Table S14: **Target concentration estimates using tagged fraction models**

Pore	Ratio	$[B]_0$	3 parameter model equation (5)			2 parameter model equation (6)		
ID	X	(nM)	$[B]_0^e$ (nM)	CV	PE	$[B]_0^e$ (nM)	CV	PE
P11	2	1	1 ± 0.25	24 %	4 %	1.1 ± 0.24	22 %	10 %
	5	2.5	2.4 ± 0.53	22 %	-5 %	2.4 ± 0.5	21 %	-3.7 %
P12	1	0.5	0.51 ± 0.027	5.2 %	2.7 %	0.15 ± 0.019	12 %	-69 %
	2	1	0.91 ± 0.048	5.3 %	-9.4 %	0.56 ± 0.055	9.8 %	-44 %
	5	2.5	3.7 ± 0.33	8.9 %	48 %	4.5 ± 0.7	15 %	81 %
	10	5	3.6 ± 0.22	6.1 %	-27 %	4.4 ± 0.49	11 %	-12 %
P13	1	0.5	0.51 ± 0.032	6.3 %	1 %	0.11 ± 0.025	24 %	-79 %
	2	1	0.95 ± 0.082	8.7 %	-5.3 %	0.7 ± 0.13	18 %	-30 %
	5	2.5	3 ± 0.51	17 %	18 %	3.2 ± 0.63	20 %	28 %
	10	5	4.3 ± 1.1	27 %	-14 %	na	–	–

* CV (coefficient of variation) is the standard deviation divided by the mean for $[B]_0^e$ converted to a percentage, and PE (percentage error) $= 100 \times ([B]_0^e - [B]_0)/[B]_0$. The "na" is reported when the estimate has a CV of 100% or greater.

The tagged capture rates for P11-P13 are reported in Table S15 and the model fitting results are reported in Table S16. The R_T trends appear to remain in sub-saturation for P11 and P13, though P13 was closer to saturation as measured by Q data. Only P12 would be expected to have meaningful parameter values, with K_d again between 0.01 and 1 nM.

Table S15: **Tagged capture rate data**

Pore ID	Ratio X	Tagged capture rate R_T	fit R^2
P11	2	5.56 ± 0.847 min^{-1}	0.811
	5	14.5 ± 1.52 min^{-1}	0.936
	10	28.2 ± 1.74 min^{-1}	0.964
P12	1	12.7 ± 1.63 min^{-1}	0.867
	2	48.6 ± 4.21 min^{-1}	0.995
	5	108 ± 7.93 min^{-1}	0.995
	10	118 ± 5.72 min^{-1}	0.994
P13	1	4.4 ± 0.989 min^{-1}	0.963
	2	11.8 ± 1.44 min^{-1}	0.925
	5	22.1 ± 2.22 min^{-1}	0.933
	10	39.1 ± 3.39 min^{-1}	0.986

* R_T and R^2 calculations are defined in section 8.1.

Table S16: **Fitted parameter values from modeling tagged capture rate**

Pore	3 parameter model equation (7)				2 parameter model equation (8)		
ID	$\alpha\beta$ (nM sec)$^{-1}$	K_d (nM)	q_1	NRMS	$\alpha\beta$ (nM sec)$^{-1}$	K_d (nM)	NRMS
P11	6.3 ± 27	1.1 ± 1.9e+02	0.044 ± 0.66	7e-06 %	13 ± 22	64 ± 1.1e+02	2.9 %
P12	4.9 ± 0.32	0.015 ± 0.0099	0.017 ± 0.007	4.5 %	4.9 ± 0.63	1.1 ± 0.56	16 %
P13	4.3 ± 19	14 ± 1.7e+02	1.2 ± 1.4	2.8 %	7 ± 19	21 ± 67	3.8 %

* Parameters and normalized root-mean-square error (NRMS) are defined in section 8.1.

The model-based target concentration predictions using R_T for P11-P13 are reported in Table S17. The two parameter models generate estimates with reasonable performance for the sub-saturating data P11 and P13. The best performance is observed for the sub-saturating P12 data, which results from a three parameter model fit to a complete data set that includes sub-saturating and saturating concentrations.

Lastly, the two-stage workflow results for pores P11-P13 are provided in Table S18. The two stage approach works well for data that is well fit by the two parameter model (P11, P13), since the method is based on the inversion of this model. In such cases, averaging the estimates generated from using Q and R_T data further improves the accuracy. Specifically, the averaged two stage estimates for P11 are 1.05 ± 0.27 nM (CV=26%, PE=4.8%) and 1.01 ± 0.15 nM (CV=14%, PE=1.2%) for $[B]_0 = 1$nM ($X = 2$) spiking in $Y = 3$ and $Y = 8$, respectively, and 2.4 ± 0.5 nM (CV=22%, PE=-3.6%) for $[B]_0 = 2.5$nM ($X = 5$) spiking in $Y = 5$. Only two of the averaged estimates for P13 were comparable in performance, namely 1.03 ± 0.31 nM (CV=29%, PE=3.2%) and 1.04 ± 0.16 nM (CV=15%, PE=3.7%) for $[B]_0 = 1$nM ($X = 2$) spiking in $Y = 3$ and $Y = 8$, respectively.

Table S17: **Target concentration estimates from modeling tagged capture rate**

Pore ID	Ratio X	$[B]_0$ (nM)	3 parameter model equation (5)			2 parameter model equation (6)		
			$[B]_0^e$ (nM)	CV	PE	$[B]_0^e$ (nM)	CV	PE
P11	2	1	1 ± 0.18	18 %	-2e-06 %	0.93 ± 0.15	16 %	-6.9 %
	5	2.5	2.5 ± 0.33	13 %	-1.4e-05 %	2.5 ± 0.32	13 %	-0.97 %
	10	5	5 ± 0.65	13 %	-7.1e-06 %	5 ± 0.82	16 %	0.22 %
P12	1	0.5	0.51 ± 0.03	5.8 %	1.9 %	0.15 ± 0.039	27 %	-71 %
	2	1	0.93 ± 0.086	9.2 %	-6.7 %	0.7 ± 0.19	27 %	-30 %
	5	2.5	3.1 ± 3	98 %	22 %	na	–	–
P13	1	0.5	0.41 ± 0.11	28 %	-18 %	0.45 ± 0.11	24 %	-10 %
	2	1	1.2 ± 0.19	17 %	17 %	1.2 ± 0.2	16 %	25 %
	5	2.5	2.4 ± 0.35	15 %	-4.1 %	2.5 ± 0.35	14 %	-1.3 %

* CV (coefficient of variation) is the standard deviation divided by the mean for $[B]_0^e$ converted to a percentage, and PE (percentage error) = $100 \times ([B]_0^e - [B]_0)/[B]_0$. The "na" is reported when the estimate has a CV of 100% or greater.

Table S18: **Target concentration estimates from two-stage workflow**

Pore ID	Ratio $X \to X + Y$	$[B]_0$ (nM)	Tagged fraction Q			Tagged capture rate R_T		
			$[B]_0^e$ (nM)	CV	PE	$[B]_0^e$ (nM)	CV	PE
P11	2 → 5	1	1.2 ± 0.43	36 %	19 %	0.9 ± 0.33	37 %	-9.6 %
	2 → 10	1	1.1 ± 0.23	20 %	11 %	0.91 ± 0.19	21 %	-8.7 %
	5 → 10	2.5	2.4 ± 0.86	36 %	-5.8 %	2.4 ± 0.62	26 %	-2.3 %
P12	1 → 2	0.5	0.18 ± 0.026	15 %	-65 %	0.13 ± 0.026	20 %	-73 %
	1 → 5	0.5	0.13 ± 0.014	11 %	-74 %	0.12 ± 0.02	16 %	-76 %
	1 → 10	0.5	0.15 ± 0.016	11 %	-69 %	0.14 ± 0.02	14 %	-71 %
	2 → 5	1	0.45 ± 0.036	8.1 %	-55 %	0.55 ± 0.11	19 %	-45 %
	2 → 10	1	0.57 ± 0.045	8 %	-43 %	0.7 ± 0.1	14 %	-30 %
P13	1 → 2	0.5	0.092 ± 0.022	24 %	-82 %	0.29 ± 0.18	63 %	-43 %
	1 → 5	0.5	0.09 ± 0.018	20 %	-82 %	0.45 ± 0.15	33 %	-11 %
	1 → 10	0.5	0.1 ± 0.021	20 %	-79 %	0.46 ± 0.12	26 %	-7.5 %
	2 → 5	1	0.57 ± 0.079	14 %	-43 %	1.5 ± 0.6	40 %	50 %
	2 → 10	1	0.7 ± 0.086	12 %	-30 %	1.4 ± 0.3	22 %	38 %
	5 → 10	2.5	na	–	–	2.6 ± 0.93	36 %	4.7 %

* Based on K_d value from 2-parameter model fittings, the method is defined in section 9 with X the unknown and Y the known ratio. CV (coefficient of variation) is the standard deviation divided by the mean for $[B]_0^e$ converted to a percentage; PE (percentage error) = $100 \times ([B]_0^e - [B]_0)/[B]_0$. The "na" is reported when the estimate has a CV of 100% or greater.

The model fit curves are plotted for pores P11-P13 and for tagged fraction Q and tagged capture rate R_T data in Figure S17. In half of the plots, the three parameter model fit is clearly superior, while in the other half the models fit the data equally well. A higher number of data points below and above saturation can help determine the superior model.

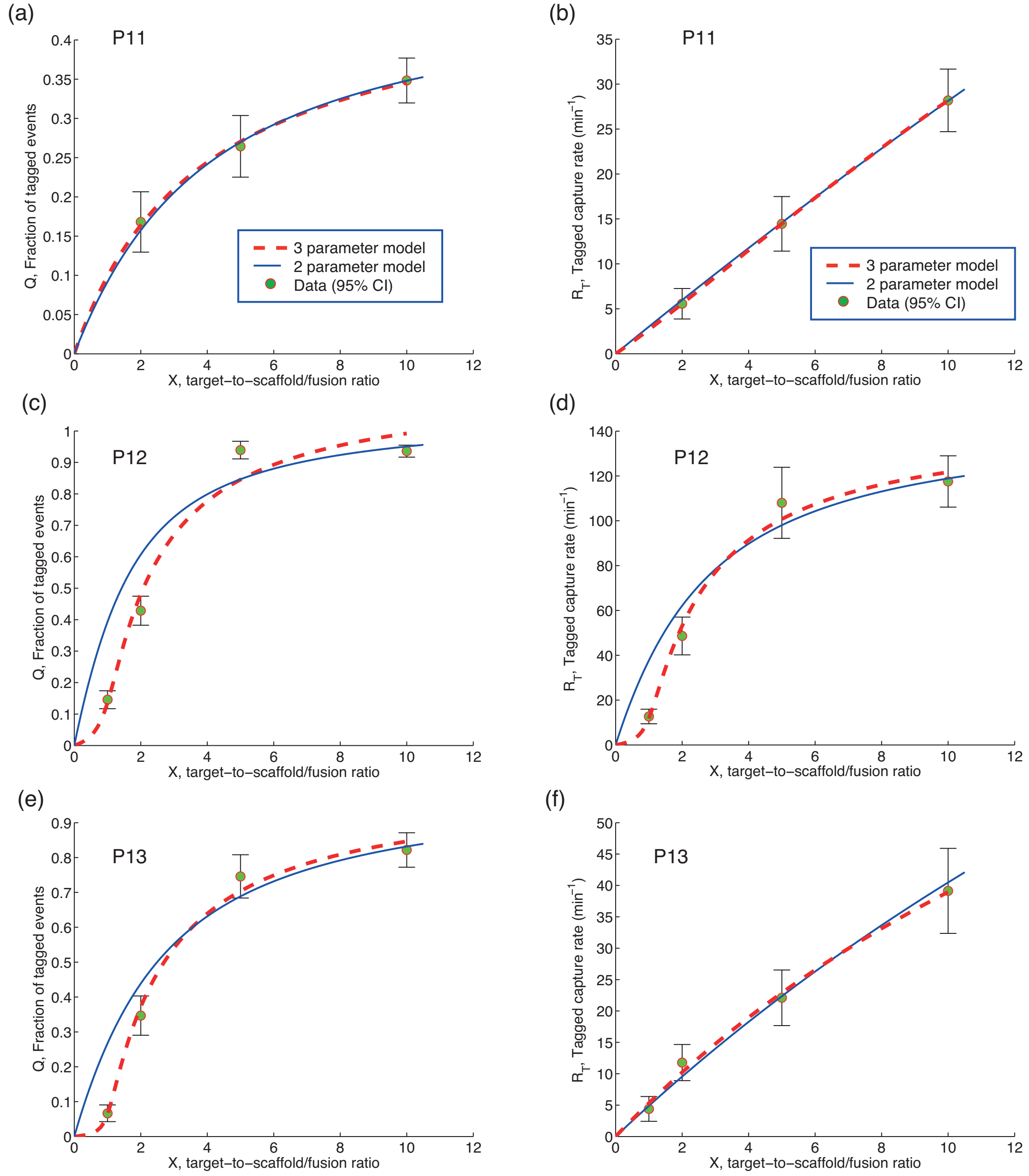

Figure S17: Data and model fit curves of tagged fraction Q and capture rate R_T vs. target to scaffold/fusion concentration ratio X for pore experiments (a,b) P11 (c,d) P12 and (e,f) P13. The Q and R_T data are not always consistent for a given pore.

11.4 Pores P14-P17: Holders vs. test strips, and shorter scaffolds

As a demonstration of reagent modularity that is intrinsic to our method, we tested DNA scaffolds (217 bp, 108 bp) shorter than those used previously (1074 bp, 3250 bp). All scaffolds comprised the same sequence for binding the PNA-peptide for HGD65 target detection. The first experiment using nanopore P14 tested the 217 bp scaffold with PNA-peptide fusion using the same holder and bench top amplifier (700B) previously validated. Two subsequent experiments with 217 bp (P15, P16) and an experiment with 108 bp scaffold (P17) used test strips and a MOM amplifier. The results are summarized in Table S19.

Table S19: **Shorter scaffolds for HGD65 detection (holder + 700B, test strip + MOM)**

Pore ID, Diam., Setup	Reagents & Concentrations*	Ratio† X	Tagged % ‡ (99% CI)	No. Events	Rec. Time	TTP 99%♮
P14	1. DNA^b/PNA-pep 4 nM	0	2 ± 1.1%	1002	8 min	np
27-29 nm	2. DNA^b/PNA-pep 4 nM + Ab 11.2 nM	2.8	91.1 ± 2.8%	694	13 min	0.59 sec
Holder	3. DNA^b/PNA-pep 4 nM + Ab 5.6 nM	1.4	86.8 ± 2.9%	873	11 min	0.1 sec
700B	4. DNA^b/PNA-pep 4 nM + Ab 2.8 nM	0.7	66.2 ± 4.5%	717	18 min	14 sec
	5. DNA^b/PNA-pep 4 nM + Ab 1.4 nM	0.35	27.4 ± 4.4%	691	12 min	10 sec
	6. DNA^b/PNA-pep 4 nM + Ab 14.8 nM	3.7	84.8 ± 3.2%	850	10 min	1.7 sec
	7. DNA^b/PNA-pep 4 nM + Ab 29.6 nM	7.4	91.3 ± 2.8%	693	10 min	0.18 sec
P15	1. DNA^b/PNA-pep 2 nM	0	1.89 ± 1.6%	475	7 min	np
33-38 nm	2. DNA^b/PNA-pep 2 nM + Ab 5.6 nM	2.8	86.1 ± 3.1%	819	17 min	4.3 sec
Test Strip	3. DNA^b/PNA-pep 2 nM + Ab 2.8 nM	1.4	82.6 ± 3.7%	685	14 min	0.47 sec
MOM	4. DNA^b/PNA-pep 2 nM + Ab 1.4 nM	0.7	34.1 ± 4.9%	615	13 min	13 sec
	5. DNA^b/PNA-pep 2 nM + Ab 0.7 nM	0.35	7.75 ± 3%	516	10 min	5.2 min
	6. DNA^b/PNA-pep 2 nM + Ab 7.4 nM	3.7	90.2 ± 3%	663	16 min	4.8 sec
	7. DNA^b/PNA-pep 2 nM + Ab 14.8 nM	7.4	89.3 ± 2.8%	783	14 min	12 sec
P16	1. DNA^b/PNA-pep 2 nM + Ab 2 nM	1	65.3 ± 2.7%	2089	15 min	2.2 sec
29-31 nm	2. DNA^b/PNA-pep 2 nM + Ab 5 nM	2.5	64.8 ± 2.9%	1802	15 min	5.2 sec
Test Strip	3. DNA^b/PNA-pep 2 nM + Ab 10 nM	5	69.2 ± 2.5%	2236	15 min	0.43 sec
MOM	4. DNA^b/PNA-pep 2 nM + Ab 20 nM	10	71.7 ± 2.5%	2121	15 min	0.028 sec
	5. 1:250 serum dilution	–	1.99 ± 0.81%	1960	10 min	np
P17	1. DNA^c/PNA-pep 10 nM	0	1.83 ± 2.7%	164	20 min	np
29-30 nm	2. DNA^c/PNA-pep 10 nM + Ab 5 nM	0.5	19.7 ± 4.2%	600	11 min	56 sec
Test Strip	3. DNA^c/PNA-pep 10 nM + Ab 10 nM	1	94.1 ± 1.9%	989	13 min	1.5 sec
MOM	4. DNA^c/PNA-pep 10 nM + Ab 20 nM	2	94.5 ± 1.7%	1245	10 min	0.11 sec

* Experiments were conducted at buffer pH 8.8, 1M LiCl, 10 mM Tris, 1 mM EDTA, and 100 mV.

† X is the dimensionless target to scaffold/fusion concentration ratio, defined only when the complete detection reagent (scaffold/fusion) competent to bind target is present.

‡ Events are tagged positive if: max $\delta G > 2.7$ nS for P14; max $\delta G > 2.5$ nS for P15, P17; max $\delta G > 2.9$ nS for P16. The reported % are $Q \pm Q_{\text{conf}}$ as defined in Section 6.

♮ TTP (time-to-positive) is the first time at which the tagged percentage minus the 99% CI is greater than, and remains greater than, a false-positive threshold of 2%, as detailed in Section 6. The "np" entry for each reagent set reflects when this criteria is not satisfied.

$^{a\text{-}c}$ DNA^a = 1074 bp, DNA^b = 217 bp, DNA^c = 108 bp.

Pores P14 and P15 tested precisely the same reagents, modulo a two-fold difference in concentration, by two different experimenters side-by-side using the two device configurations (holder+700B, and test strip + MOM). Pore P16 did not generate enough Q values below half-maximum to permit fitting with the 3 parameter, while 2 parameter model fitting results are reported. In a preliminary experiment, P16 used a 1:250 dilution of serum background without target or detection reagent as the negative control used to establish the tagging criteria for 2% false positives. Serum background experiments are explored further in pores P18-20. Pore P17 data did not include enough target concentrations, particularly below saturation, to permit modeling and quantitation. Nonetheless, P17 did show positive detection in all cases and with the shortest scaffold (108 bp) tested in this study.

The data trends and modeling results are consistent using the two different device interfaces, i.e., tradition holder and 700B for P14 and test strip with MOM for P15, suggesting equivalent performance for the purpose of target protein detection and quantitation. The tagged fraction model fitting performance for P14-P16 are reported in Table S20. Comparing the P14 and P15 results, as observed previously (P12, P13), the three parameter model fits the data better than the two parameter model, particularly during the transition to saturation. For these data sets, the predicted $\alpha \approx 0.9$ is conserved for both data and model sets. On the other hand, the three and two parameter models do not predict comparable K_d values. The values predicted by the two parameter model are more reliable, however. Specifically, since the transitions to saturation data has only two data sets (i.e., two Q values sufficiently below α), and the three parameter model uses two of three parameters to fit to the sub-saturation range, the data is only marginally sufficient for determining unique parameter values. More than three sub-saturating data values are needed to infer the two parameters more reliably. The $K_d \approx 0.2$ nM prediction using the 2 parameter model is thus treated as a more reliable estimate, and was achieved even with the sparser data set for P16.

Table S20: **Fitted parameter values from modeling tagged fraction**

Pore	3 parameter model equation (3)				2 parameter model equation (4)		
ID	α	K_d (nM)	q_1	NRMS	α	K_d (nM)	NRMS
P14	0.89 ± 0.0059	2.3e-16 ± 0.011	1 ± 0.059	5 %	0.91 ± 0.012	0.2 ± 0.079	7.7 %
P15	0.9 ± 0.0086	0.019 ± 0.0064	0.27 ± 0.022	2.2 %	0.91 ± 0.014	0.19 ± 0.06	14 %
P16	NaN	NaN	NaN	–	0.72 ± 0.011	0.23 ± 0.066	96 %

* Parameters and normalized root-mean-square error (NRMS) are defined in section 7.1. The "NaN" value is reported when the model fails to fit the data.

The model-based target concentration predictions using Q for P14 and P15 are reported in Table S21. Prediction was limited to at most $X = 1.4$ for which Q was sufficiently below saturation. Over this range, the three parameter model estimates outperformed the two parameter model estimates. Quantitation of target concentration was not performed for P16, due to the lack of sub-saturating ratios X which resulted poor model fitting performance. The tagged capture rates for P14-P17 are reported in Table S22. The model fitting results for P14-P16 are reported in Table S23.

Table S21: **Target concentration estimates using tagged fraction models**

Pore ID	Ratio X	$[B]_0$ (nM)	3 parameter model equation (5)			2 parameter model equation (6)		
			$[B]_0^e$ (nM)	CV	PE	$[B]_0^e$ (nM)	CV	PE
P14	0.35	1.4	1.2 ± 0.095	7.7 %	-12 %	1.3 ± 0.086	6.7 %	-8.3 %
	0.7	2.8	3 ± 0.091	3 %	6.5 %	3.4 ± 0.2	5.9 %	23 %
P15	0.35	0.7	0.53 ± 0.072	14 %	-24 %	0.19 ± 0.03	16 %	-73 %
	0.7	1.4	1.4 ± 0.053	3.7 %	2.7 %	0.86 ± 0.056	6.5 %	-38 %
	1.4	2.8	2.8 ± 0.31	11 %	-0.86 %	3.7 ± 0.55	15 %	32 %

* CV (coefficient of variation) is the standard deviation divided by the mean for $[B]_0^e$ converted to a percentage, and PE (percentage error) $= 100 \times ([B]_0^e - [B]_0)/[B]_0$.

Table S22: **Tagged capture rate data**

Pore ID	Ratio X	Tagged capture rate R_T	fit R^2
P14	2.8	57.4 ± 2.72 min^{-1}	0.998
	1.4	95.5 ± 4.13 min^{-1}	0.982
	0.7	28.9 ± 1.58 min^{-1}	0.986
	0.35	18.2 ± 1.57 min^{-1}	0.992
	3.7	99 ± 4.39 min^{-1}	0.995
	7.4	76.1 ± 3.6 min^{-1}	0.996
P15	2.8	75.8 ± 3.4 min^{-1}	0.998
	1.4	51.7 ± 2.58 min^{-1}	0.997
	0.7	16.8 ± 1.38 min^{-1}	0.992
	0.35	5.19 ± 0.977 min^{-1}	0.967
	3.7	40.2 ± 1.96 min^{-1}	0.993
	7.4	53.2 ± 2.39 min^{-1}	0.995
P16	1	114 ± 3.69 min^{-1}	0.999
	2.5	92.7 ± 3.23 min^{-1}	0.999
	5	112 ± 3.38 min^{-1}	0.999
	10	114 ± 3.47 min^{-1}	1
P17	0.5	12.5 ± 1.37 min^{-1}	0.996
	1	77.1 ± 3.01 min^{-1}	0.999
	2	127 ± 4.4 min^{-1}	0.998

* R_T and R^2 calculations are defined in section 8.1.

Table S23: **Fitted parameter values from modeling tagged capture rate**

Pore ID	3 parameter model equation (7)				2 parameter model equation (8)		
	$\alpha\beta$ (nM sec)$^{-1}$	K_d (nM)	q_1	NRMS	$\alpha\beta$ (nM sec)$^{-1}$	K_d (nM)	NRMS
P14	0.34 ± 0.0083	2.7e-15 ± 3.4e-15	0.28 ± 0.025	17 %	0.34 ± 0.01	0.1 ± 0.06	22 %
P15	0.47 ± 0.012	0.006 ± 0.0079	0.19 ± 0.02	15 %	0.47 ± 0.0081	0.089 ± 0.11	20 %
P16	NaN	NaN	NaN	–	0.96 ± 0.032	0.27 ± 0.13	78 %

* Parameters and normalized root-mean-square error (NRMS) are defined in section 8.1. The "NaN" value is reported when the model fails to fit the data.

The consensus from the two parameter model using Q and R_T data is that K_d is between 0.1 and 0.2 nM, when considering K_d estimates with a CV below 100%. The model fitting performance is not as good with R_T data as it is with Q data, a result of higher fluctuations in capture rate observed above saturation. We observe such capture rate fluctuations to occur occasionally with single solid-state nanopore data, even when measuring different aliquots of the same reagent on the same pore sequentially over time. In such cases, its seems the pore itself and the way in which it interacts with molecules of interest can shift the event population distributions. Averaging the R_T values for P14 and P15 creates a more stable rate above saturation. Specifically, the average rates at $X = 2.8, 1.4, 3.7, 7.4$ are 74, 67, 70, and 65 min^{-1}, with the standard deviation of these four mean rates at 4 min^{-1} down from 20 and 15 min^{-1} for P14 and P15, respectively. Modeling the average rates also improved the fitting performance (NRMS = 5% and 17% for the three and two parameter models respectively). This suggests that arrayed measurements aggregated to generate mean capture rates could produce more robust data for modeling and quantitation.

The model-based target concentration predictions using R_T for P14 and P15 are reported in Table S24. Only the three parameter model fit generates reasonable estimates for the sub-saturating concentrations, with P15 achieving the best performance.

Table S24: **Target concentration estimates from modeling tagged capture rate**

Pore ID	Ratio X	$[B]_0$ (nM)	3 parameter model equation (5)			2 parameter model equation (6)		
			$[B]_0^e$ (nM)	CV	PE	$[B]_0^e$ (nM)	CV	PE
P14	0.35	1.4	2 ± 0.13	6.5 %	44 %	0.92 ± 0.25	27 %	-34 %
	0.7	2.8	2.6 ± 0.1	3.8 %	-5.8 %	na	–	–
	1.4	5.6	4.2 ± 0.059	1.4 %	-26 %	4 ± 0.093	2.3 %	-29 %
P15	0.35	0.7	0.7 ± 0.11	15 %	0.63 %	na	–	–
	0.7	1.4	1.4 ± 0.069	4.9 %	-0.032 %	na	–	–

* CV (coefficient of variation) is the standard deviation divided by the mean for $[B]_0^e$ converted to a percentage, and PE (percentage error) = $100 \times ([B]_0^e - [B]_0)/[B]_0$. The "na" is reported when the estimate has a CV of 100% or greater.

The event plots and model fitting results for pores P14 and P15 are shown in Figures S18 and S19. Interestingly, the shorter scaffold reveals two deeper event populations, as revealed in the scatter plots (Figures S18a, S19a). The EMSA assay used to proof the reagents also showed a dominant band and a fainter band higher in the lane (main text, Figure 3a). We do not here attempt to resolve whether the presence of more than one molecular configurations is the source for the two subpopulations, though it is clear that the deepest subpopulation is sparser (see histogram Figures S18b). For simplicity, both are lumped together in the "tagged positive" category. The stability of the Q data compared to the R_T data above saturation is shown by comparing Figures S18c-d and Figure S19b-c. Note that the fluctuations in R_T are a reflection of variation in the total event capture rate, since Q fluctuations are small and the total capture rate is approximately R_T/Q. In most cases, the three parameter model fits the transition-to-saturation data, and thus generates the more accurate concentration estimates.

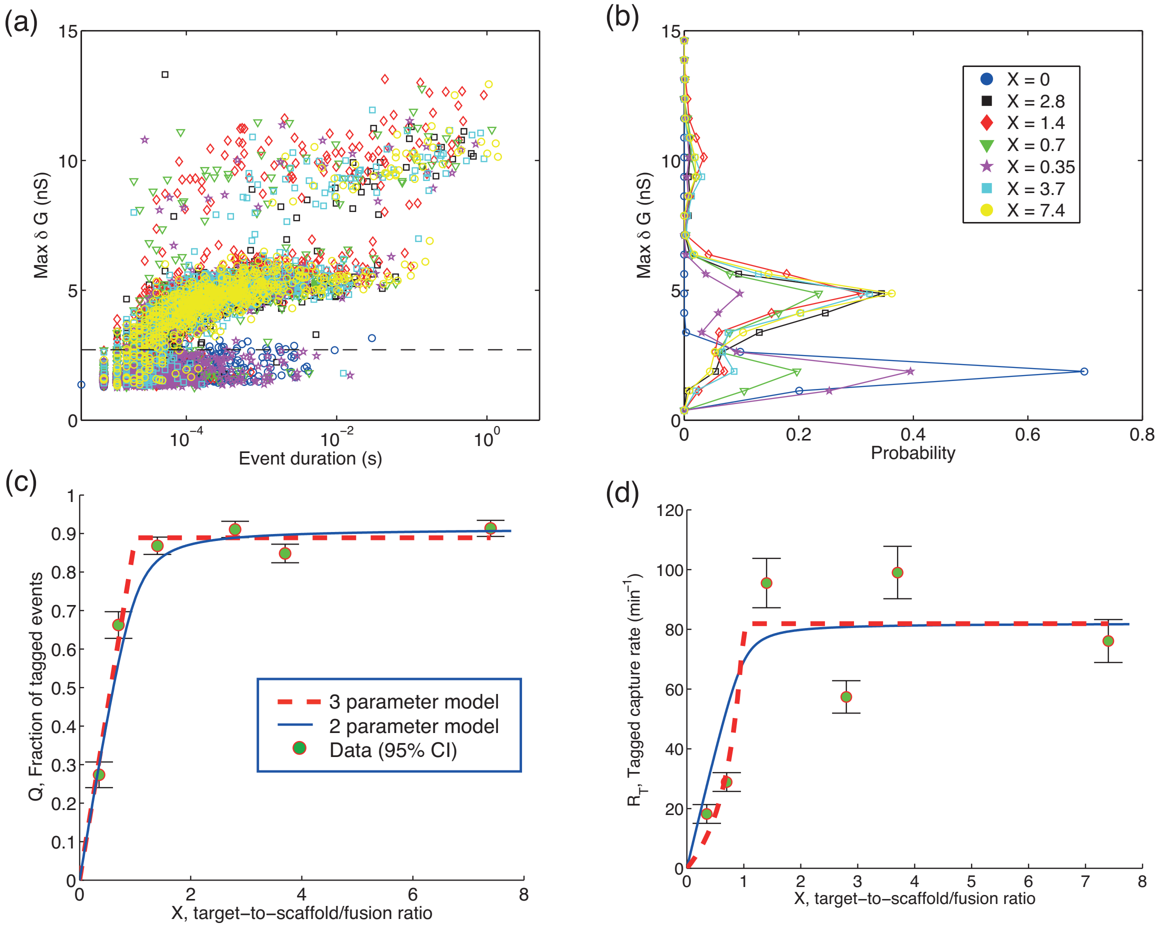

Figure S18: Pore P14 experiment. (a) All-event scatter plots of max δG vs. duration and (b) the corresponding histograms for max δG, varying the target to scaffold/fusion concentration ratio X. The horizontal line in (a) shows the max δG threshold above which events are tagged positive. (c,d) Data and model fit curves of tagged fraction Q and tagged capture rate R_T vs. X. The models fit the data well except for the R_T data above saturation, where fluctuations in capture rate are observed.

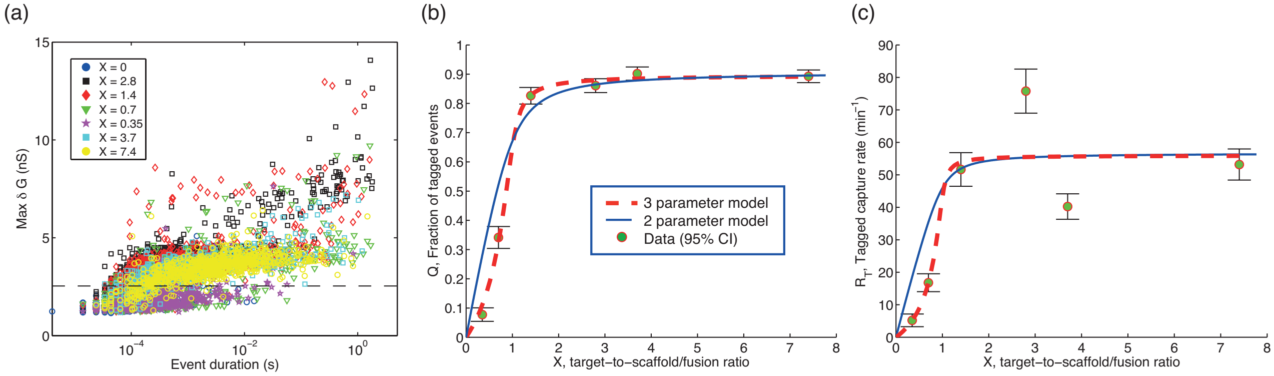

Figure S19: Pore P15 experiment. (a) All-event scatter plots of max δG vs. duration varying the target to scaffold/fusion concentration ratio X. The horizontal line shows the max δG threshold above which events are tagged positive. (b,c) Data and model fit curves of tagged fraction Q and tagged capture rate R_T vs. X. As with P14, the models fit the data well except for the R_T data above saturation, where fluctuations in capture rate are observed.

Lastly, the two-stage workflow results for pores P14 and P15 are provided in Table S25. The two stage approach only generates reasonable estimates for P14 and Q data, where the two parameter model matches the transition to saturation data well. In all other cases, the data falling below the two parameter model during the transition to saturation is the cause for the negative PE values.

Table S25: **Target concentration estimates from two-stage workflow**

Pore ID	Ratio $X \to X+Y$	$[B]_0$ (nM)	Tagged fraction Q $[B]_0^e$ (nM)	CV	PE	Tagged capture rate R_T $[B]_0^e$ (nM)	CV	PE
P14	0.35 → 0.7	1.4	0.92 ± 0.1	11 %	-34 %	2.1 ± 0.4	20 %	47 %
	0.35 → 1.4	1.4	1.2 ± 0.084	6.9 %	-13 %	0.73 ± 0.075	10 %	-48 %
	0.35 → 2.8	1.4	1.3 ± 0.081	6.5 %	-11 %	1.3 ± 0.13	10 %	-7.4 %
	0.35 → 3.7	1.4	1.4 ± 0.089	6.6 %	-2.9 %	0.75 ± 0.074	9.9 %	-46 %
	0.35 → 7.4	1.4	1.3 ± 0.082	6.5 %	-9.1 %	0.98 ± 0.097	9.8 %	-30 %
	0.7 → 1.4	2.8	3.3 ± 0.14	4.4 %	18 %	1 ± 0.094	9.1 %	-63 %
	0.7 → 2.8	2.8	3.3 ± 0.13	3.9 %	19 %	2.1 ± 0.16	7.6 %	-26 %
	0.7 → 3.7	2.8	3.7 ± 0.18	4.7 %	33 %	1.2 ± 0.085	7.1 %	-57 %
	0.7 → 7.4	2.8	3.4 ± 0.14	4 %	21 %	1.6 ± 0.12	7.5 %	-44 %
P15	0.35 → 0.7	0.7	0.19 ± 0.04	21 %	-72 %	0.3 ± 0.084	28 %	-57 %
	0.35 → 1.4	0.7	0.16 ± 0.026	16 %	-77 %	0.18 ± 0.037	20 %	-74 %
	0.35 → 2.8	0.7	0.19 ± 0.03	16 %	-73 %	0.14 ± 0.027	19 %	-80 %
	0.35 → 3.7	0.7	0.18 ± 0.029	16 %	-74 %	0.27 ± 0.054	20 %	-62 %
	0.35 → 7.4	0.7	0.19 ± 0.03	16 %	-73 %	0.2 ± 0.038	19 %	-71 %
	0.7 → 1.4	1.4	0.71 ± 0.055	7.6 %	-49 %	0.55 ± 0.068	12 %	-61 %
	0.7 → 2.8	1.4	0.86 ± 0.055	6.3 %	-38 %	0.45 ± 0.042	9.4 %	-68 %
	0.7 → 3.7	1.4	0.84 ± 0.053	6.3 %	-40 %	0.88 ± 0.089	10 %	-37 %
	0.7 → 7.4	1.4	0.87 ± 0.054	6.2 %	-38 %	0.67 ± 0.065	9.8 %	-52 %

* Based on K_d value from 2-parameter model fittings, the method is defined in section 9 with X the unknown and Y the known ratio. CV (coefficient of variation) is the standard deviation divided by the mean for $[B]_0^e$ converted to a percentage; PE (percentage error) $= 100 \times ([B]_0^e - [B]_0)/[B]_0$.

11.5 Pores P18-P20: Detection above serum background

The 217 bp DNA scaffold with PNA-peptide was tested with varying HGD65 target concentrations in the presence of serum background. As stated in the methods section 11.1, the detection reagent is incubated with 10% serum, then diluted 25X to the stated concentrations. The results from three experiments (pores P18-20) are summarized in Table S26. Rather then using a negative control on each pore to establish the tagging threshold, the criteria max $\delta G > 2.8$ nS was applied across all pores. Only P18 tested enough target-to-detection reagent ratios for modeling, although the event rate reduced considerably for reagents 4 and 5 due to periods of partial clogging. Trimming of the data applied uniformly to all reagents was required to remove events that occurred during partial clogging of the channel. The trimming criteria was to remove all events with an open channel RMS greater than 30 pA. The open channel RMS was computed in two ways: first, as the standard deviation of the 200 samples before and after each event; and second, as the standard deviation of all open channel samples from the end of the previous events until the start of the current event. An event was removed if either of these values exceeded 30 pA. When the channel was never partially clogged, the presence of the serum background significantly enhanced the capture rate (P19). Regardless of the presence of partial clogging and consequent trimming, the titration of the target in the presence of the serum showed positive detection in all cases and increasing Q with X. The saturating Q value was reduced when more serum background events were detected (P19), as expected.

Table S26: **HGD65 detection in serum background with common tagging threshold**

Pore ID, Diam., Setup	Reagents & Concentrations*	Ratio† X	Tagged % ‡ (99% CI)	No. Events	Rec. Time	TTP 99%♮
P18	1. DNA^b/PNA-pep 2 nM + Ab 14.8 nM	7.4	85.8 ± 3.9%	534	12 min	0.28 sec
30-32 nm	2. 1:250 serum background (SeB)	–	1.71 ± 1.7%	409	2 min	np
Test Strip	3. DNA^b/PNA-pep 2 nM + Ab 7.4 nM + SeB	3.7	90.8 ± 3%	618	15 min	13 sec
MOM	4. DNA^b/PNA-pep 2 nM + Ab 5.56 nM + SeB	2.78	71 ± 15%	62	9 min	10 sec
	5. DNA^b/PNA-pep 2 nM + Ab 2.78 nM + SeB	1.39	34.8 ± 13%	92	8 min	2.6 min
P19	1. 1:250 serum background (SeB)	–	0.635 ± 0.67%	945	4 min	np
33-34 nm	2. DNA^b/PNA-pep 4 nM + Ab 29.6 nM + SeB	7.4	12.9 ± 1.9%	2117	5 min	7.5 sec
Holder	3. DNA^b/PNA-pep 4 nM + Ab 29.6 nM + SeB	7.4	11.3 ± 1.3%	4068	8 min	44 sec
700B	4. DNA^b/PNA-pep 4 nM + Ab 14.8 nM + SeB	3.7	7.62 ± 1.1%	3975	4 min	6.8 sec
P20	1. 1:250 serum background (SeB)	–	1.27 ± 0.84%	1179	8 min	np
38-40 nm	2. DNA^b/PNA-pep 2 nM + Ab 1.4 nM + SeB	0.7	21.7 ± 4.5%	552	13 min	1.1 min
TS/MOM	3. DNA^b/PNA-pep 2 nM + Ab 2.8 nM + SeB	1.4	61.3 ± 5%	633	13 min	10 sec

* Experiments were conducted at buffer pH 8.8, 1M LiCl, 10 mM Tris, 1 mM EDTA, and 100 mV.
† X is the dimensionless target to scaffold/fusion concentration ratio, defined only when the complete detection reagent (scaffold/fusion) competent to bind target is present.
‡ Events are tagged positive if: max $\delta G > 2.8$ nS for P18-P20. The reported % are $Q \pm Q_{\text{conf}}$ as defined in Section 6.
♮ TTP (time-to-positive) is the first time at which the tagged percentage minus the 99% CI is greater than, and remains greater than, a false-positive threshold of 2%, as detailed in Section 6. The "np" entry for each reagent set reflects when this criteria is not satisfied.
$^{a\text{-}c}$ DNA^a = 1074 bp, DNA^b = 217 bp, DNA^c = 108 bp.

Despite the clogging problems with P18, applying the three and two parameter model fitting to

the data could provide a phenomenological curve with which we can assess concentration prediction. The tagged fraction model fitting performance for P18 is reported in Table S27. The model-based target concentration predictions for P18 are reported in Table S28. The prediction performance for this preliminary result is consistent with the results in buffer only. More thorough testing and modeling of Q data is required to assess the performance of the method in serum background. The tagged capture rates R_T are reported in Table S29, and in the presence of serum background did follow the $R_T \propto X$ trend predictably enough to permit modeling.

Table S27: **Fitted parameter values from modeling tagged fraction**

Pore	3 parameter model equation (3)				2 parameter model equation (4)		
ID	α	K_d (nM)	q_1	NRMS	α	K_d (nM)	NRMS
P18	1 ± 0.031	0.00071 ± 0.036	0.00053 ± 0.023	11 %	0.92 ± 0.033	0.83 ± 0.32	25 %

* Parameters and normalized root-mean-square error (NRMS) are defined in section 7.1.

Table S28: **Target concentration estimates using tagged fraction models**

Pore	Ratio	$[B]_0$	3 parameter model equation (5)			2 parameter model equation (6)		
ID	X	(nM)	$[B]_0^e$ (nM)	CV	PE	$[B]_0^e$ (nM)	CV	PE
P18	1.39	2.78	2.7 ± 0.19	7 %	-2.8 %	1.3 ± 0.26	21 %	-55 %
	2.78	5.56	5.2 ± 1	20 %	-7.2 %	4.3 ± 1.7	39 %	-23 %

* CV (coefficient of variation) is the standard deviation divided by the mean for $[B]_0^e$ converted to a percentage, and PE (percentage error) = $100 \times ([B]_0^e - [B]_0)/[B]_0$.

Table S29: **Tagged capture rate data**

Pore ID	Ratio X	Tagged capture rate R_T	fit R^2
P18	7.4	58.8 ± 3.27 min^{-1}	0.993
	3.7	49.5 ± 2.49 min^{-1}	0.997
	2.78	5.31 ± 0.952 min^{-1}	0.983
	1.39	4.3 ± 0.904 min^{-1}	0.978
P19	7.4	98.3 ± 7.08 min^{-1}	0.995
	7.4	79.2 ± 4.39 min^{-1}	0.999
	3.7	116 ± 7.93 min^{-1}	0.993
P20	0.7	11.3 ± 1.23 min^{-1}	0.993
	1.4	33.2 ± 2.01 min^{-1}	0.982

* R_T and R^2 calculations are defined in section 8.1.

11.6 Pores P21-P24: Detection and quantitation above saliva background

The 217 bp DNA scaffold with PNA-peptide was tested with varying HGD65 target concentrations in the presence of saliva background. As stated in the methods section 11.1, the detection reagent is incubated with 10% saliva and varying amounts of HGD65 Ab for 5 minutes, then diluted 10X to the stated concentrations at 1% saliva. The results from four experiments (pores P21-24) are summarized in Table S30. Rather then using the negative control on each pore to establish the tagging threshold, the criteria max $\delta G > 2.8$ nS was applied across all pores. Establishing a uniform criteria can reduce the number of controls needed, and thereby simplify the workflow. This also served to test if a "look-up" value could be used in future implementations.

Partial clogging of the channel that would require trimming did not occur during these experiments. It appears that diluted saliva (1:100) created fewer background events than the diluted serum (1:250) that was tested. The titration of the target in the presence of the saliva showed positive detection in all cases and increasing Q with X. The saturating Q value was reduced to $\sim$0.7 due to saliva background events, but appeared to be conserved across pores.

Table S30: **HGD65 detection & quantitation in saliva background with test strips/MOM**

Pore ID, Diam., Setup	Reagents & Concentrations*	Ratio† X	Tagged % ‡ (99% CI)	No. Events	Rec. Time	TTP 99%♮
P21	1. DNA^b/PNA-pep 1.33 nM + 1% SaB$^♭$	0	0.873 ± 1.6%	229	5 min	np
31-34 nm	2. DNA^b/PNA-pep 1.33 nM + Ab 0.266 nM + SaB	0.2	13 ± 6.2%	193	5 min	3.1 min
Test Strip	3. DNA^b/PNA-pep 1.33 nM + Ab 1.33 nM + SaB	1	56.6 ± 7.1%	320	5 min	0.86 sec
MOM	4. DNA^b/PNA-pep 1.33 nM + Ab 6.65 nM + SaB	5	69.7 ± 4.9%	594	5 min	2.1 sec
P22	1. DNA^b/PNA-pep 1.33 nM + 1% SaB$^♭$	0	1.79 ± 2%	279	5 min	np
33-35 nm	2. DNA^b/PNA-pep 1.33 nM + Ab 0.266 nM + SaB	0.2	6.91 ± 3.6%	333	5 min	3.1 min
Test Strip	3. DNA^b/PNA-pep 1.33 nM + Ab 0.665 nM + SaB	0.5	30.6 ± 6.1%	373	5 min	18 sec
MOM	4. DNA^b/PNA-pep 1.33 nM + Ab 1.33 nM + SaB	1	37.7 ± 7.2%	302	5 min	16 sec
P23	1. DNA^b/PNA-pep 1.33 nM + 1% SaB$^♭$	0	3.12 ± 7.9%	32	5 min	np
40-42 nm	2. DNA^b/PNA-pep 1.33 nM + Ab 0.266 nM + SaB	0.2	34 ± 12%	106	5 min	60 sec
Test Strip	3. DNA^b/PNA-pep 1.33 nM + Ab 1.33 nM + SaB	1	56.4 ± 7.2%	319	5 min	3.4 sec
MOM	4. DNA^b/PNA-pep 1.33 nM + Ab 6.65 nM + SaB	5	71.9 ± 5.2%	491	5 min	0.15 sec
	5. DNA^b/PNA-pep 1.33 nM + Ab 2.66 nM + SaB	2	64 ± 5.2%	575	5 min	6.1 sec
	6. DNA^b/PNA-pep 1.33 nM + Ab 0.665 nM + SaB	0.5	54.5 ± 8%	257	5 min	9.3 sec
	7. DNA^b/PNA-pep 1.33 nM + Ab 13.3 nM + SaB	10	62.9 ± 6%	429	5 min	2.5 sec
P24	1. DNA^b/PNA-pep 1.33 nM + 1% SaB$^♭$	0	0 ± 0%	57	4 min	np
31-35 nm	2. DNA^b/PNA-pep 1.33 nM + Ab 2.66 nM + SaB	2	67.7 ± 12%	99	5 min	24 sec
Test Strip	3. DNA^b/PNA-pep 1.33 nM + Ab 0.665 nM + SaB	0.5	33.6 ± 10%	143	5 min	39 sec
MOM	4. DNA^b/PNA-pep 1.33 nM + Ab 13.3 nM + SaB	10	66.7 ± 5.3%	534	5 min	0.17 sec

* Experiments were conducted at buffer pH 8.8, 1M LiCl, 10 mM Tris, 1 mM EDTA, and 100 mV.

† X is the dimensionless target to scaffold/fusion concentration ratio, defined only when the complete detection reagent (scaffold/fusion) competent to bind target is present.

‡ Events are tagged positive if: max $\delta G > 2.8$ nS for P21-P24. The reported % are $Q \pm Q_{\text{conf}}$ as defined in Section 6.

♮ TTP (time-to-positive) is the first time at which the tagged percentage minus the 99% CI is greater than, and remains greater than, a false-positive threshold of 2%, as detailed in Section 6. The "np" entry for each reagent set reflects when this criteria is not satisfied.

$^{a\text{-}c}$ DNA^a = 1074 bp, DNA^b = 217 bp, DNA^c = 108 bp.

♭ 1% SaB (or just, SaB) is 1:100 saliva sample background.

The tagged fraction model fitting performance is reported in Table S31. The model-based target concentration predictions are reported in Table S32. The prediction performance is consistent with the results in buffer only (P14-P17). The three parameter model fits and Q data for P21-P24 are superimposed in Figure S20a. The transitions to saturation and the saturation value appear to be consistent.

Table S31: **Fitted parameter values from modeling tagged fraction**

Pore	3 parameter model equation (3)				2 parameter model equation (4)		
ID	α	K_d (nM)	q_1	NRMS	α	K_d (nM)	NRMS
P21	0.71 ± 0.023	0.061 ± 0.07	0.96 ± 0.28	6.1e-06 %	0.71 ± 0.019	0.071 ± 0.036	1.9 %
P22	0.47 ± 0.043	0.083 ± 3.8e-07	1.5 ± 0.31	13 %	0.35 ± 0.51	0.16 ± 1.4	36 %
P23	0.68 ± 0.017	0.66 ± 11	7.5 ± 54	9 %	NaN	NaN	–
P24	0.67 ± 0.021	3.2e-16 ± 7.1	1 ± 12	1.2 %	0.68 ± 0.019	0.081 ± 0.075	6.2 %

* Parameters and normalized root-mean-square error (NRMS) are defined in section 7.1. The "NaN" value is reported when the model fails to fit the data.

Table S32: **Target concentration estimates using tagged fraction models**

Pore	Ratio	$[B]_0$	3 parameter model equation (5)			2 parameter model equation (6)		
ID	X	(nM)	$[B]_0^e$ (nM)	CV	PE	$[B]_0^e$ (nM)	CV	PE
P21	0.2	0.266	0.266 ± 0.07	26 %	-2.2e-05 %	0.26 ± 0.049	19 %	-2.3 %
	1	1.33	1.33 ± 0.18	14 %	6.9e-07 %	1.4 ± 0.19	14 %	1.6 %
P22	0.2	0.266	0.15 ± 0.039	26 %	-45 %	0.3 ± 0.097	33 %	12 %
	0.5	0.665	0.84 ± 0.15	18 %	27 %	na	–	–
P23	0.2	0.266	0.25 ± 0.078	31 %	-6.9 %	na	–	–
	0.5	0.665	0.84 ± 0.3	35 %	27 %	na	–	–
	1	1.33	0.98 ± 0.39	39 %	-26 %	na	–	–
P24	0.5	0.665	0.665 ± 0.12	18 %	1.7e-09 %	0.73 ± 0.1	14 %	10 %

* CV (coefficient of variation) is the standard deviation divided by the mean for $[B]_0^e$ converted to a percentage, and PE (percentage error) = $100 \times ([B]_0^e - [B]_0)/[B]_0$. The "na" is reported when the estimate has a CV of 100% or greater.

The tagged capture rates for P21-P24 are reported in Table S33, and the model fitting results for P21-P23 are reported in Table S34. The P24 data only had one sub-saturating X value and so the model was not used for prediction purposes. The model fitting performance is not as good with R_T data as it is with Q data, a result of higher fluctuations in capture rate observed above saturation. This is consistent with other data using the same scaffold even with buffer only (P14, P15), suggesting that the fluctuations are not increased by the presence of diluted sample. As suggested by earlier analysis, it is possible that averaging tagged capture rates across a set of pores that simultaneously measure a common reagent set could improve R_T modeling and concentration prediction. The three parameter model fits and R_T data for P21-P24 are superimposed in Figure S20b.

Table S33: **Tagged capture rate data**

Pore ID	Ratio X	Tagged capture rate R_T	fit R^2
P21	0.2	4.42 ± 1.05 min^{-1}	0.872
	1	41.5 ± 3.67 min^{-1}	0.988
	5	104 ± 6.1 min^{-1}	0.998
P22	0.2	7.21 ± 1.79 min^{-1}	0.936
	0.5	28.2 ± 3.14 min^{-1}	0.994
	1	30.7 ± 3.42 min^{-1}	0.997
P23	0.2	6.85 ± 1.36 min^{-1}	0.974
	1	35.6 ± 3.16 min^{-1}	0.997
	5	76.7 ± 4.86 min^{-1}	0.994
	2	76.2 ± 4.73 min^{-1}	0.995
	0.5	29.7 ± 2.99 min^{-1}	0.985
	10	53.1 ± 3.85 min^{-1}	0.998
P24	2	15.1 ± 2.19 min^{-1}	0.982
	0.5	8.09 ± 1.39 min^{-1}	0.96
	10	82.3 ± 5.19 min^{-1}	0.992

* R_T and R^2 calculations are defined in section 8.1.

Table S34: **Fitted parameter values from modeling tagged capture rate**

Pore ID	3 parameter model equation (7)				2 parameter model equation (8)		
	$\alpha\beta$ (nM sec)$^{-1}$	K_d (nM)	q_1	NRMS	$\alpha\beta$ (nM sec)$^{-1}$	K_d (nM)	NRMS
P21	1.4 ± 0.14	0.1 ± 0.11	0.18 ± 0.053	2.1e-06 %	2 ± 0.35	2.9 ± 1.3	3.1 %
P22	0.47 ± 0.075	0.083 ± 0.66	2 ± 0.61	17 %	0.38 ± 0.15	0.059 ± 0.3	37 %
P23	0.87 ± 0.037	0.093 ± 0.036	0.63 ± 0.13	15 %	NaN	NaN	–
P24	28 ± 11	17 ± 35	0.05 ± 0.33	3.3 %	12 ± 14	1.4e+02 ± 1.8e+02	3.6 %

* Parameters and normalized root-mean-square error (NRMS) are defined in section 8.1. The "NaN" value is reported when the model fails to fit the data.

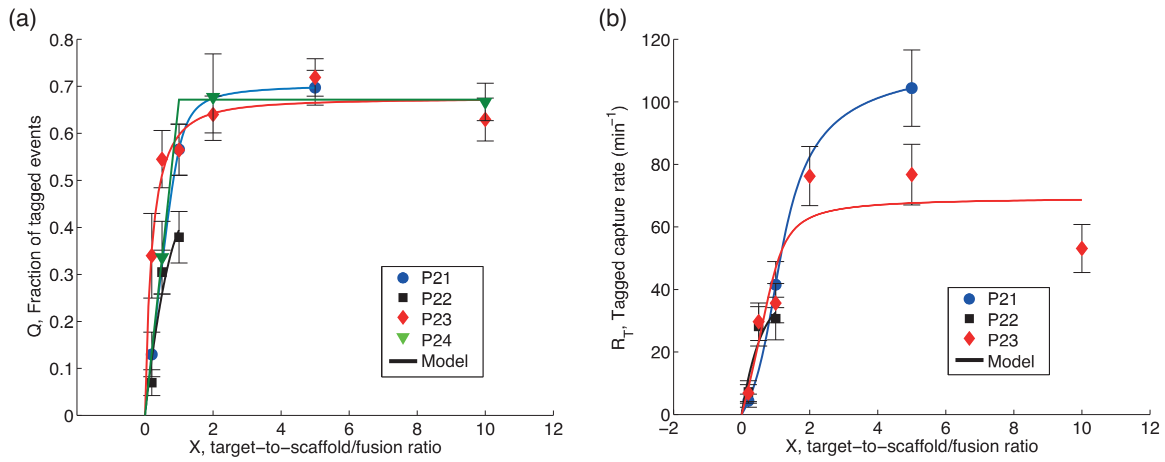

Figure S20: (a) Data (95% CI) and three parameter model fit curves of tagged fraction Q vs. X for pores P21-P24. (b) Data (95% CI) and three parameter model fit curves of tagged capture rate R_T vs. X for pores P21-P23.

The model-based target concentration predictions using R_T are reported in Table S35 for P21-P23. The two-stage workflow results using a common $K_d = 0.1$ nM value are provided in Table S36 for P21-P24. Pore P24 was included as a model-free data set. Similar to using a common tagging criteria value for the four pores P21-P24, a common K_d value was used in the two-stage method to assess the performance of a look-up value that could be used in future implementations. By using a previously determined value, the two-stage method is a "model free" method, in the sense that it is free of a model fitting step as discussed in section 9. The two-stage results show that the Q data outperformed the R_T data in most cases, as in the model-based prediction results.

Table S35: **Target concentration estimates from modeling tagged capture rate**

Pore	Ratio	$[B]_0$	3 parameter model equation (5)			2 parameter model equation (6)		
ID	X	(nM)	$[B]_0^e$ (nM)	CV	PE	$[B]_0^e$ (nM)	CV	PE
P21	0.2	0.266	0.266 ± 0.078	29 %	3.8e-05 %	0.12 ± 0.033	27 %	-54 %
	1	1.33	1.33 ± 0.12	8.8 %	-4.4e-06 %	1.4 ± 0.21	15 %	5 %
P22	0.2	0.266	0.15 ± 0.054	36 %	-43 %	0.33 ± 0.1	31 %	24 %
P23	0.2	0.266	0.21 ± 0.05	23 %	-19 %	na	–	–
	0.5	0.665	0.83 ± 0.097	12 %	25 %	na	–	–
	1	1.33	0.99 ± 0.11	11 %	-26 %	na	–	–

* CV (coefficient of variation) is the standard deviation divided by the mean for $[B]_0^e$ converted to a percentage, and PE (percentage error) $= 100 \times ([B]_0^e - [B]_0)/[B]_0$. The "na" is reported when the estimate has a CV of 100% or greater.

Table S36: **Target concentration estimates from two-stage workflow**

Pore ID	Ratio $X \rightarrow X+Y$	$[B]_0$ (nM)	Tagged fraction Q			Tagged capture rate R_T		
			$[B]_0^e$ (nM)	CV	PE	$[B]_0^e$ (nM)	CV	PE
P21	$0.2 \rightarrow 1$	0.266	0.251 ± 0.053	21 %	-5.6 %	0.11 ± 0.03	28 %	-60 %
	$0.2 \rightarrow 5$	0.266	0.265 ± 0.047	18 %	-0.39 %	0.06 ± 0.014	24 %	-78 %
	$1 \rightarrow 5$	1.33	1.45 ± 0.19	13 %	9.2 %	0.58 ± 0.068	12 %	-56 %
P22	$0.2 \rightarrow 0.5$	0.266	0.112 ± 0.031	27 %	-58 %	0.13 ± 0.049	37 %	-51 %
	$0.2 \rightarrow 1$	0.266	0.195 ± 0.046	23 %	-27 %	0.26 ± 0.08	31 %	-2.8 %
P23	$0.5 \rightarrow 5$	0.665	1.28 ± 0.15	12 %	93 %	0.57 ± 0.076	13 %	-15 %
	$1 \rightarrow 5$	1.33	1.36 ± 0.15	11 %	2.3 %	0.69 ± 0.089	13 %	-48 %
	$1 \rightarrow 10$	1.33	na	–	–	1.1 ± 0.21	20 %	-19 %
P24	$0.5 \rightarrow 2$	0.665	0.704 ± 0.11	15 %	5.8 %	na	–	–
	$0.5 \rightarrow 10$	0.665	0.764 ± 0.099	13 %	15 %	0.14 ± 0.026	19 %	-79 %

* Setting $K_d = 0.1$ nM for all data sets, the method is defined in section 9 with X the unknown and Y the known ratio. CV (coefficient of variation) is the standard deviation divided by the mean for $[B]_0^e$ converted to a percentage; PE (percentage error) $= 100 \times ([B]_0^e - [B]_0)/[B]_0$. The "na" is reported when the estimate has a CV of 100% or greater.

11.7 Pores P25-P26: Detection and quantitation above saliva background with 30 second incubation

The 217 bp DNA scaffold with PNA-peptide was incubated with 10% saliva and varying amounts of HGD65 Ab for 30 seconds, then diluted 10X to the stated concentrations at 1% saliva. These experiments reduced the incubation period from 5 minutes to see how rapidly a sample-to-answer workflow could produce quantitative results. The results from two experiments (pores P25 and P26) are summarized in Table S37. The tabulated results used the negative control on each pore to establish the tagging threshold. Using a common criteria max $\delta G > 2.5$ nS both pores did not appreciably change the results. Specifically, the only TTP values that changed (up to the reported two significant figures) was an increase from 17 sec to 25 sec ($Q \pm Q_{\mathrm{conf}} = 30.6 \pm 7\%$) for reagent #6 on P25, and a decrease from 3.1 min to 2.4 min ($Q \pm Q_{\mathrm{conf}} = 9.06 \pm 4.6\%$) for reagent #2 on P26. Additionally, the modeling and prediction performance was not appreciably affected by using a common tagging criteria value instead of the value established on each pore.

Table S37: **HGD65 quantitation after 30 second incubation in 10% saliva background followed by 10X dilution using test strips/MOM**

Pore ID, Diam., Setup	Reagents & Concentrations*	Ratio† X	Tagged % ‡ (99% CI)	No. Events	Rec. Time	TTP 99%♮
P25	1. DNA^b/PNA-pep 1.33 nM+ 1% SaB$^\flat$	0	1.88 ± 2.4%	213	5 min	np
40-44 nm	2. DNA^b/PNA-pep 1.33 nM + Ab 0.266 nM + SaB	0.2	37.7 ± 7%	321	5 min	12 sec
Test Strip	3. DNA^b/PNA-pep 1.33 nM + Ab 1.33 nM + SaB	1	49.9 ± 4.8%	725	5 min	1.6 sec
MOM	4. DNA^b/PNA-pep 1.33 nM + Ab 6.65 nM + SaB	5	79.6 ± 5.8%	318	4 min	0.83 sec
	5. DNA^b/PNA-pep 1.33 nM + Ab 2.66 nM + SaB	2	73.8 ± 7.4%	233	5 min	3.6 sec
	6. DNA^b/PNA-pep 1.33 nM + Ab 0.665 nM+ SaB	0.5	33.7 ± 7.1%	291	5 min	17 sec
	7. DNA^b/PNA-pep 1.33 nM + Ab 13.3 nM + SaB	10	74.2 ± 6%	353	5 min	7.7 sec
P26	1. DNA^b/PNA-pep 1.33 nM+ 1% SaB$^\flat$	0	1.79 ± 3.2%	112	5 min	np
40-46 nm	2. DNA^b/PNA-pep 1.33 nM + Ab 0.266 nM+ SaB	0.2	8.66 ± 4.5%	254	5 min	3.1 min
Test Strip	3. DNA^b/PNA-pep 1.33 nM + Ab 0.665 nM+ SaB	0.5	28.1 ± 7.7%	228	5 min	23 sec
MOM	4. DNA^b/PNA-pep 1.33 nM + Ab 1.33 nM + SaB	1	64.3 ± 7.6%	263	5 min	9.6 sec
	5. DNA^b/PNA-pep 1.33 nM + Ab 2.66 nM + SaB	2	75.9 ± 7.5%	216	5 min	7.9 sec
	6. DNA^b/PNA-pep 1.33 nM + Ab 6.65 nM + SaB	5	87.2 ± 5.7%	227	5 min	5.4 sec
	7. DNA^b/PNA-pep 1.33 nM + Ab 13.3 nM + SaB	10	89.9 ± 3.5%	504	5 min	10 sec

* Experiments were conducted at buffer pH 8.8, 1M LiCl, 10 mM Tris, 1 mM EDTA, and 100 mV.

† X is the dimensionless target to scaffold/fusion concentration ratio, defined only when the complete detection reagent (scaffold/fusion) competent to bind target is present.

‡ Events are tagged positive if: max $\delta G > 2.3$ nS for P25, and max $\delta G > 2.6$ nS for P26. The reported % are $Q \pm Q_{\mathrm{conf}}$ as defined in Section 6.

♮ TTP (time-to-positive) is the first time at which the tagged percentage minus the 99% CI is greater than, and remains greater than, a false-positive threshold of 2%, as detailed in Section 6. The "np" entry for each reagent set reflects when this criteria is not satisfied.

$^{a\text{-}c}$ DNA^a = 1074 bp, DNA^b = 217 bp, DNA^c = 108 bp.

$^\flat$ 1% SaB (or just, SaB) is 1:100 saliva sample background.

The tagged fraction model fitting performance is reported in Table S38. The P25 data was not as well fit as the P26 data. While P26 measured monotonically increasing amounts of target with detection reagent, P25 measured a more random order. Earlier results (P14, P15) show that target concentrations do not need to be measured in strictly increasing amounts for the models to fit the data well. Thus, the fitting performance discrepancy is unlikely due to the order of the target concentrations being tested. The model-based target concentration predictions are reported in Table S39. The prediction performance is better for P26 since the model fit is better.

To emulate a two nanopore array, we can average the Q values across the two pores and apply the model fitting and concentration prediction methods. Each pore can use the tagging thresholds established from each $X = 0$ case, i.e., max $\delta G > 2.3$ nS for P25 and max $\delta G > 2.6$ nS for P26. The average values for $X = (0.2, 0.5, 1, 2, 5, 10)$ are $Q = (0.25, 0.31, 0.54, 0.75, 0.83, 0.83)$ with error bars $Q_{\text{conf}} = (0.04, 0.04, 0.03, 0.04, 0.03, 0.02)$. Observe that the error bars are smaller for the mean data than for the individual pores. This is because the total number of events N is increased, being the sum of the number of events on both pores, and $Q_{\text{conf}} \propto 1/\sqrt{N}$. The results of model fitting applied to the average data is reported in Table S38. The fit performance and most of the fitted parameter values are between those values for P25 and P26 individually, which is not too surpising. The target concentration predictions for the averaged data is reported in Table S39.

Table S38: **Fitted parameter values from modeling tagged fraction**

Pore	3 parameter model equation (3)				2 parameter model equation (4)		
ID	α	K_d (nM)	q_1	NRMS	α	K_d (nM)	NRMS
P25	0.81 ± 0.02	7.7 ± 18	15 ± 33	17 %	0.77 ± 0.017	0.044 ± 0.039	25 %
P26	0.88 ± 0.019	0.069 ± 0.045	0.57 ± 0.1	3.5 %	0.92 ± 0.016	0.29 ± 0.076	6 %
MD$^\flat$	0.88 ± 0.015	0.7 ± 0.49	1.8 ± 0.66	6.9 %	0.85 ± 0.016	0.18 ± 0.067	9.1 %

* Parameters and normalized root-mean-square error (NRMS) are defined in section 7.1. The "NaN" value is reported when the model fails to fit the data.

$^\flat$ MD = mean data. The model fitting is applied to the averaged Q values and error bars, which are reported in the main text.

Table S39: **Target concentration estimates using tagged fraction models**

Pore ID	Ratio X	$[B]_0$ (nM)	3 parameter model equation (5)			2 parameter model equation (6)		
			$[B]_0^e$ (nM)	CV	PE	$[B]_0^e$ (nM)	CV	PE
P25	0.2	0.266	0.51 ± 0.085	17 %	90 %	0.69 ± 0.063	9.2 %	1.6e+02 %
	0.5	0.665	0.41 ± 0.074	18 %	-38 %	0.61 ± 0.063	10 %	-7.8 %
	1	1.33	0.93 ± 0.12	13 %	-30 %	0.94 ± 0.073	7.7 %	-29 %
P26	0.2	0.266	0.23 ± 0.052	23 %	-15 %	0.16 ± 0.03	19 %	-41 %
	0.5	0.665	0.65 ± 0.074	11 %	-1.9 %	0.54 ± 0.07	13 %	-19 %
	1	1.33	1.4 ± 0.14	9.8 %	6.4 %	1.6 ± 0.21	13 %	21 %
	2	2.66	2 ± 0.38	19 %	-27 %	2.5 ± 0.49	20 %	-6.1 %
MD$^\flat$	0.2	0.266	0.4 ± 0.048	12 %	51 %	0.46 ± 0.041	8.8 %	74 %
	0.5	0.665	0.54 ± 0.058	11 %	-19 %	0.59 ± 0.051	8.5 %	-11 %
	1	1.33	1.3 ± 0.091	7.3 %	-5.9 %	1.2 ± 0.098	8.5 %	-13 %
	2	2.66	3.3 ± 0.55	17 %	23 %	2.5 ± 0.53	21 %	-5.5 %

* CV (coefficient of variation) is the standard deviation divided by the mean for $[B]_0^e$ converted to a percentage, and PE (percentage error) = $100 \times ([B]_0^e - [B]_0)/[B]_0$. The "na" is reported when the estimate has a CV of 100% or greater.

$^\flat$ MD = mean data. The predicted values are based on the model fitted to the averaged Q values, reported in Table S38.

Since the error bars are smaller for the mean data, the CV values are smaller for the MD predictions than for the individual pore predictions. On the other hand, the PE values are not uniformly improved. When the P25 and P26 PE values have opposite sign and comparable magnitude, the prediction based on the averaged data tends to be an improvement over the individual pores. Of course, the mean PE is achieved when one averages the P25 and P26 predictions directly, instead of computing MD predictions which are based on fitting to the averaged data.

An interesting question is whether it is better to model and generate predictions in a distributed fashion, and then compute an aggregate consensus prediction and uncertainty estimates therefrom, or to aggregate the data for a single model-fitting-and-prediction analysis. Our data suggests that, in most cases, averaging the predictions based on modeling the individual pores will outperform predictions based on a single model fitted to averaged data. In the case of P25 and P26, averaging the 3-parameter $X = 0.2$ predictions produces the estimate 0.37 nM with PE = 39%, an improvement over 51% for the tabulated (MD) result. Averaging the 2-parameter $X = 1$ predictions is also an improvement over the mean data prediction, although the opposite is true for the 3-parameter predictions. A more comprehensive analysis of averaging predictions is explored in section 12.2. The data and model fits for P25, P26 and the mean data are shown in Figure S21.

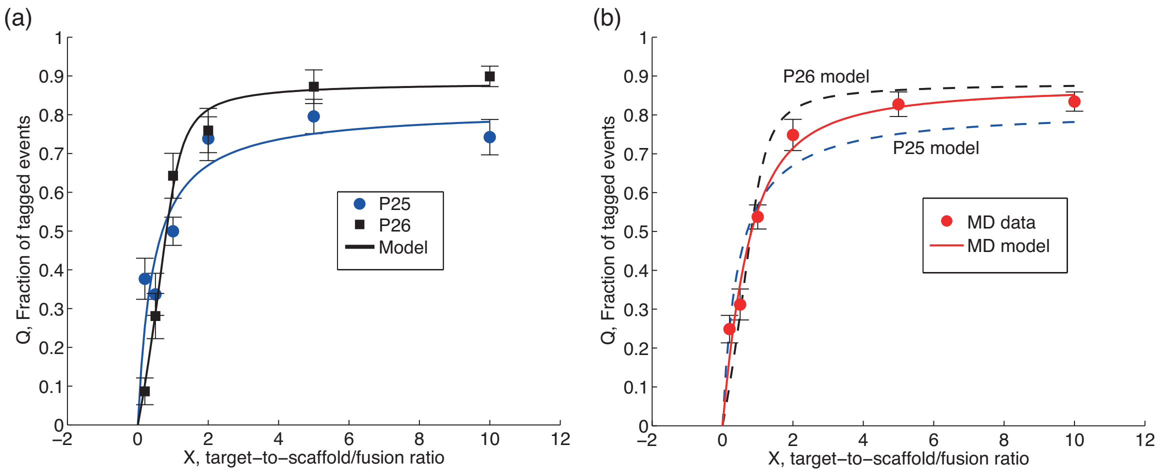

Figure S21: Data (95% CI) and three parameter model fit curves of tagged fraction Q vs. X for (a) pores P25 and P26, and (b) the mean data (MD) for pores P25 and P26. The P25 and P26 model fits are re-plotted (dashed lines) in (b).

The tagged capture rates for P25 and P26 are reported in Table S40, and the model fitting results are reported in Table S41. As with P21-P24, the model fitting performance is not as good with R_T data as it is with Q data, a result of higher fluctuations in capture rate, below and above saturation. The model-based target concentration predictions using R_T are reported in Table S42. The P26 model fitting and prediction results are superior to those of P25, though both are inferior to the results with Q data.

Table S40: **Tagged capture rate data**

Pore ID	Ratio X	Tagged capture rate R_T	fit R^2
P25	0.2	27.5 ± 2.97 min^{-1}	0.993
	1	70.4 ± 4.4 min^{-1}	0.998
	5	69.5 ± 5.2 min^{-1}	0.996
	2	35.4 ± 3.21 min^{-1}	0.981
	0.5	20 ± 2.41 min^{-1}	0.988
	10	54.5 ± 4.01 min^{-1}	0.995
P26	0.2	5.54 ± 1.41 min^{-1}	0.922
	0.5	14.3 ± 2.12 min^{-1}	0.991
	1	38.5 ± 3.53 min^{-1}	0.967
	2	38.9 ± 3.62 min^{-1}	0.966
	5	47.8 ± 4.04 min^{-1}	0.934
	10	103 ± 5.76 min^{-1}	0.998

* R_T and R^2 calculations are defined in section 8.1.

Table S41: **Fitted parameter values from modeling tagged capture rate**

Pore ID	3 parameter model equation (7)				2 parameter model equation (8)		
	$\alpha\beta$ (nM sec)$^{-1}$	K_d (nM)	q_1	NRMS	$\alpha\beta$ (nM sec)$^{-1}$	K_d (nM)	NRMS
P25	0.71 ± 0.048	9.5e-17 ± 16	1.1 ± 34	27 %	0.73 ± 0.047	0.067 ± 0.05	30 %
P26	1.9 ± 0.73	1.1e+02 ± 14	12 ± 7.2	12 %	2.1 ± 0.34	8.3 ± 2.5	12 %

* Parameters and normalized root-mean-square error (NRMS) are defined in section 8.1. The "NaN" value is reported when the model fails to fit the data.

Table S42: **Target concentration estimates from modeling tagged capture rate**

Pore ID	Ratio X	$[B]_0$ (nM)	3 parameter model equation (5)			2 parameter model equation (6)		
			$[B]_0^e$ (nM)	CV	PE	$[B]_0^e$ (nM)	CV	PE
P25	0.2	0.266	0.6 ± 0.13	22 %	1.2e+02 %	0.68 ± 0.1	15 %	1.5e+02 %
	0.5	0.665	0.43 ± 0.12	27 %	-36 %	na	–	–
	1	1.33	na	–	–	na	–	–
	2	2.66	0.78 ± 0.14	18 %	-71 %	0.9 ± 0.13	15 %	-66 %
	5	6.65	na	–	–	1.1 ± 0.073	6.7 %	-84 %
P26	0.2	0.266	0.32 ± 0.11	35 %	20 %	0.33 ± 0.091	28 %	24 %
	0.5	0.665	0.87 ± 0.22	26 %	31 %	0.89 ± 0.16	18 %	34 %
	1	1.33	2.9 ± 0.56	20 %	1.1e+02 %	2.8 ± 0.4	14 %	1.1e+02 %
	2	2.66	2.9 ± 0.56	19 %	8.6 %	2.8 ± 0.41	14 %	6.7 %
	5	6.65	3.8 ± 0.68	18 %	-42 %	3.7 ± 0.52	14 %	-44 %
	10	13.3	na	–	–	14 ± 4.5	32 %	6.9 %

* CV (coefficient of variation) is the standard deviation divided by the mean for $[B]_0^e$ converted to a percentage, and PE (percentage error) $= 100 \times ([B]_0^e - [B]_0)/[B]_0$. The "na" is reported when the estimate has a CV of 100% or greater.

The two-stage workflow results are provided in Table S43 for P25 and P26. The two-stage results show that the Q data outperformed the R_T data in most cases, as in the model-based prediction results. The P26 results suggest that inference of an X value is best when Y is large, and that combining the Q and R_T based estimates can improve the error. In particular, the combined estimates with the lowest CV and PE values are for $X + Y = 10$, i.e., the largest Y available. This trend is generally true for P25 also, despite the poor model fitting (NRMS $\geq$ 25%).

Table S43: **Target concentration estimates from two-stage workflow**

Pore ID	Ratio $X \to X+Y$	$[B]_0$ (nM)	Tagged fraction Q $[B]_0^e$ (nM)	CV	PE	Tagged capture rate R_T $[B]_0^e$ (nM)	CV	PE
P25	0.2 → 1	0.266	1.07 ± 0.15	14 %	3e+02 %	0.48 ± 0.071	15 %	80 %
	0.2 → 2	0.266	0.706 ± 0.062	8.8 %	1.7e+02 %	1.2 ± NaN	–	3.6e+02 %
	0.2 → 5	0.266	0.664 ± 0.057	8.5 %	1.5e+02 %	0.56 ± 0.082	15 %	1.1e+02 %
	0.2 → 10	0.266	0.718 ± 0.063	8.7 %	1.7e+02 %	0.73 ± 0.1	14 %	1.8e+02 %
	0.5 → 1	0.665	0.862 ± 0.11	13 %	30 %	0.24 ± 0.046	19 %	-63 %
	0.5 → 2	0.665	0.622 ± 0.056	9.1 %	-6.5 %	0.8 ± 0.15	18 %	21 %
	0.5 → 5	0.665	0.59 ± 0.051	8.6 %	-11 %	0.4 ± 0.059	15 %	-39 %
	0.5 → 10	0.665	0.637 ± 0.056	8.9 %	-4.2 %	0.52 ± 0.078	15 %	-21 %
	1 → 2	1.33	0.942 ± 0.06	6.4 %	-29 %	na	–	–
	1 → 5	1.33	0.899 ± 0.048	5.3 %	-32 %	na	–	–
	1 → 10	1.33	0.98 ± 0.053	5.4 %	-26 %	na	–	–
P26	0.2 → 0.5	0.266	0.163 ± 0.054	33 %	-39 %	0.24 ± 0.16	66 %	-9.9 %
	0.2 → 1	0.266	0.13 ± 0.028	22 %	-51 %	0.16 ± 0.049	31 %	-40 %
	0.2 → 2	0.266	0.157 ± 0.032	20 %	-41 %	0.32 ± 0.1	32 %	19 %
	0.2 → 5	0.266	0.156 ± 0.031	20 %	-42 %	0.5 ± NaN	–	90 %
	0.2 → 10	0.266	0.155 ± 0.03	20 %	-42 %	0.32 ± 0.086	27 %	21 %
	0.5 → 1	0.665	0.407 ± 0.074	18 %	-39 %	0.36 ± 0.097	27 %	-46 %
	0.5 → 2	0.665	0.538 ± 0.073	14 %	-19 %	0.91 ± 0.27	29 %	36 %
	0.5 → 5	0.665	0.534 ± 0.063	12 %	-20 %	1.5 ± 0.35	23 %	1.2e+02 %
	0.5 → 10	0.665	0.534 ± 0.063	12 %	-20 %	0.87 ± 0.15	17 %	31 %
	1 → 2	1.33	1.8 ± NaN	–	35 %	29 ± NaN	–	2.1e+03 %
	1 → 5	1.33	1.62 ± 0.19	12 %	22 %	8.9 ± NaN	–	5.7e+02 %
	1 → 10	1.33	1.61 ± 0.16	10 %	21 %	2.9 ± 0.46	16 %	1.2e+02 %
	2 → 5	2.66	2.47 ± NaN	–	-7.2 %	7.8 ± NaN	–	1.9e+02 %
	2 → 10	2.66	2.47 ± 0.45	18 %	-7.1 %	2.8 ± 0.43	16 %	3.5 %
	5 → 10	6.65	6.5 ± NaN	–	-2.3 %	2.9 ± 0.51	17 %	-56 %

* CV (coefficient of variation) is the standard deviation divided by the mean for $[B]_0^e$ converted to a percentage; PE (percentage error) = $100 \times ([B]_0^e - [B]_0)/[B]_0$. The "na" is reported when the estimate has a CV of 100% or greater. "NaN" is reported as the uncertainty estimate when the simulated data exceeds sautration, in which case inversion of the two parameter model function is undefined.

12 Summary of HIV Ab HGD65 results

12.1 Single pore quantitation performance

Summary statistics are computed by combining results from separate nanopore experiments where a common detection reagent type was used, or when a common sample type (saliva) was present. Only in one case (P14 and P15) was the same aliquot of reagents tested on different nanopores on the same date; in all other cases, the buffer and reagents were prepared and tested on a unique nanopore and date.

Precision refers to the dispersion of results from replicate measurements, where in this case a "measurement" is a reported target concentration estimate. One such measure of precision is the CV of replicate estimates, which is examined in the next section. Our methods also provide a CV value for each estimate that captures the uncertainty in both the data and the fitted model parameters, as described previously. A summary statistic for single-pore estimate precision is the average of CV values, which is reported in Table S44.

Trueness or accuracy refers to how close an estimate is to a true value. Since the estimates are generated using known target concentrations, we can use the percentage error (PE) values for combined nanopore data sets to generate a metric for trueness. The chosen metric is the median of the absolute values of PE. The absolute value is used to remove the sign but keep the magnitude of the bias, and the median was used due to the presence of outliers. Specifically, artificially small PE can occur when the number of data points is equal to the number of model parameters (e.g., P11 R_T data estimates from the 3 parameter model in Table S17). Significantly larger PE can occur for estimates near saturation also. The aggregate PE performance values are reported in Table S44.

Table S44: **Summary statistics for single-pore target quantitation performance**

Pore IDs*	$[B]_0$ range	Model	$n^\dagger$ (Q, R_T)	mean CV$^\sharp$ (Q, R_T)	median $\lvert$PE$\rvert^\flat$ (Q, R_T)
P7-P13	0.1-8 nM	3 param	(26, 24)	(13.3%, 16.4%)	(11.6%, 11.7%)
		2 param	(24, 23)	(15.9%, 21.6%)	(18.2%, 14.8%)
P14-P15	0.7-5.6 nM	3 param	(6, 10)	(9.1%, 5.2%)	(9.1%, 54%)
		2 param	(6, 7)	(10.5%, 14.8%)	(21.9%, 59%)
P21-P26	0.266-13.3 nM	3 param	(15, 18)	(20.7%, 20.5%)	(26%, 28.4%)
		2 param	(11, 15)	(14.8%, 19%)	(16.8%, 34.2%)
All	0.1-13.3 nM	3 param	(47,52)	(15.1%, 15.7%)	(12.1%, 17.5%)
		2 param	(41,45)	(14.8%, 19.7%)	(16.8%, 24.9%)

* Pores were grouped together by a common detection reagent, and in the cases of pores P21-P26 by the presence of saliva background.

† n is the number of target estimates $[B]_0^e$ included in the summary calculations. An estimate was included if the computed CV (coefficient of variation) value is less than 100%.

♯ The mean CV is the average of n CV values.

♯ The median |PE| is the median of the absolute values of n PE (percentage error) values. Median was used as a summary statistic for PE due to the presence of outliers.

The results in Table S44 suggest that target estimation performs better with Q data than with R_T data, and with the 3 parameter model rather than the 2 parameter model. The downside of the 3 parameter model is that more control data points are required for fitting the model prior to estimation. Figure S22 shows all of the individual estimates on $[B]_0^e$ vs. $[B]_0$ plots, with the Q data and the 3 parameter model showing data closest to the zero error line (Fig. S22a).

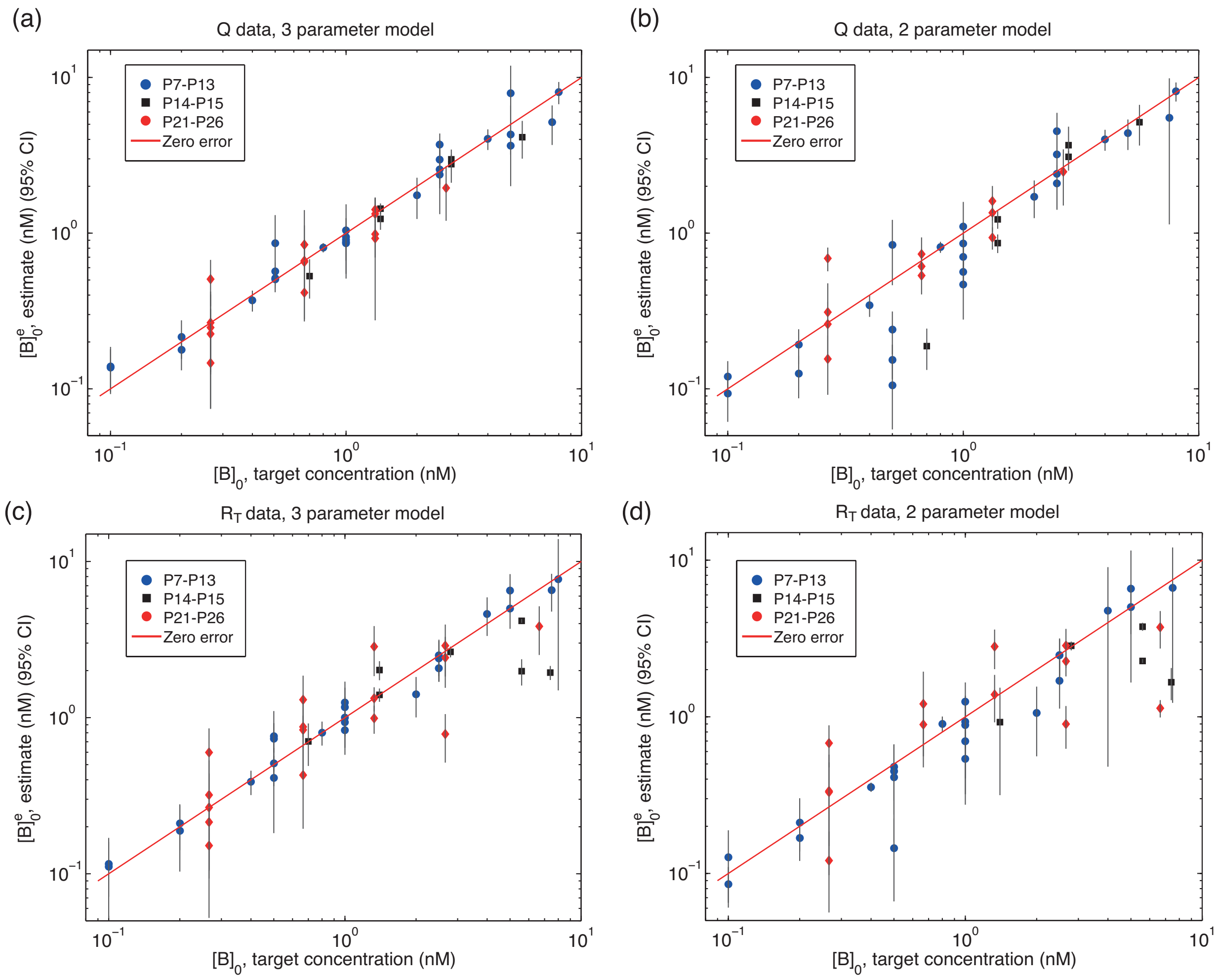

Figure S22: All $[B]_0^e$ vs. $[B]_0$ results with CV values below 100% are plotted with 95% CI. The zero error (PE = 0) line is plotted for comparison. The plots are grouped by model order and data type: (a) 3 parameter, Q data; (b) 2 parameter, Q data; (c) 3 parameter, R_T data; (d) 2 parameter, R_T data. Note: two 13.3 nM estimates are truncated from plots (c,d) to visually align the Q and R_T concentration ranges.

The quantitation performance using a single nanopore workflow is approximately 15% for CV and PE with the three parameter model. However, the spread in CV and PE values is large. A primary source for this is that estimation accuracy and precision vary across the quantitative range X. That is, if X is too low, precision goes down as the limit of positive-for-target detection is reached. Also, if X is too high, precision again suffers as the single pore measurement approaches saturation and thus prevents reliable quantitation. A plot of PE $\pm$ CV values versus X is shown in Figure S23. The best range is within $0.5 < X < 5$ for the presented results.

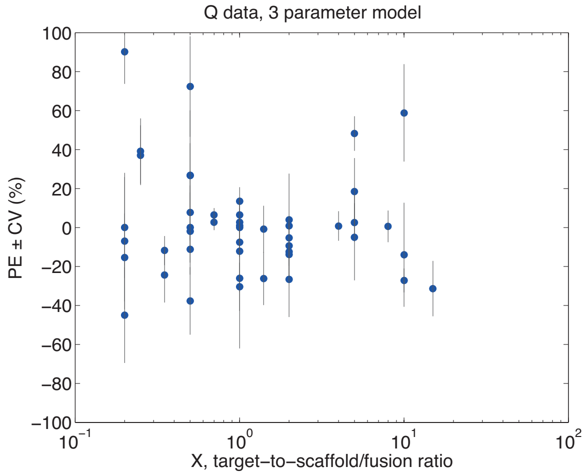

Figure S23: For all $n = 47$ target concentration estimates shown in Fig. S22a, the PE$\pm$CV values are plotted versus the corresponding X. Similar trends are observed for the other three sets in Fig. S22.

Future designs will incorporate multiple nanopores for enhanced performance and robustness. A test strip would have m independent pores that asynchronously detect molecules from a common pool. The design would then be used test replicates of controls and blinded samples to assess accuracy and precision, including inter- and intra-assay CV [16]. Until then, we can numerically combine single-pore estimates for common target concentrations to test how well quantitation performance can be improved.

12.2 Combining estimates to improve quantitation performance

Nanopore quantitation precision and accuracy is assessed by computing the average CV and PE values, respectively, from a set of estimates for common target concentrations. To assess the precision of an immunoassay workflow, an aggregate CV is typically computed from replicate estimates of controls and/or samples and compared to a standard of performance [17]. In our results, the aggregate CV and PE values computed from mean estimates for separate single nanopore experiments will capture varying amounts of **single pore reproducibility**, because experiments were under the following conditions:

1. Different people performed experiments, each using a unique MOM reader or a common 700B amplifier for measurement. Specifically: Person 1 ran P7 and P8; Person 2 ran P9, P12, and P16; Person 3 ran P10, P11, P13, P14 and P19; Person 4 ran P15, P17, P18 and P20; and Person 5 ran P21-P26.

2. Each experiment used one new test strip and nanopore chip, or a reusable holder with a new nanopore chip.

3. The nanopore size range varied across all experiments.

4. Each experiment was run on a unique date and with a unique set of prepared samples, with the exception of P14 and P15 that used aliquots from the same pool of reagents on the same date.

The average estimate results presented below emulate the performance of an m-pore arrayed test strip, where each pore would be fluidically isolated from the other pores, measuring a unique aliquot and dilution of sample and detection reagent after an incubation period. The estimates have more uncertainty than an m-pore strip due to conditions 1 and 4. Tables S45-S48 report averaged estimates and their CV and PE performance, using Q and R_T data and with the 3 parameter and 2 parameter models. Each reported average used at least two estimates. As in the previous section, pores are grouped by a common detection reagents (P7-P13, and P14-P15) and by the presence of saliva background (P21-P26).

The best performance was achieved with the Q data and the 3 parameter model shown in Table S45. The results suggest that the shorter 217 bp scaffold (pores P14-P15 and P21-P26) produced better estimation accuracy ($|\text{PE}| = 7\%$) than the longer scaffold (1074 bp, P7-P13, $|\text{PE}| = 15\%$). Meanwhile, the presence of saliva background for pores P21-P26 could be a primary cause for a loss in precision (mean CV = 32%) compared to buffer only (mean CV = 16% for pores P7-P13 and P14-P15). Aggregating all of the results, the mean CV = 21% and and the mean $|\text{PE}| = 11\%$.

Table S45: **Combined target concentration estimates: 3 param model, Q data**

Pore IDs*	$[B]_0$ (nM)	$m^\dagger$	Mean ± SD $[B]_0^e$ (nM)	CV	PE
P7-P13	0.1	2	0.138 ± 0.0015	1.09 %	38.2 %
	0.2	2	0.197 ± 0.0267	13.6 %	-1.71 %
	0.5	4	0.612 ± 0.169	27.6 %	22.4 %
	1	5	0.927 ± 0.0712	7.69 %	-7.33 %
	2.5	4	2.9 ± 0.589	20.3 %	16.1 %
	5	3	5.3 ± 2.32	43.7 %	5.91 %
Summary$^\flat$			$R^2 = 0.983$	19.0 %	|15.3| %
P14-P15	1.4	2	1.34 ± 0.143	10.7 %	-4.58 %
	2.8	2	2.88 ± 0.145	5.02 %	2.79 %
Summary$^\flat$			$R^2 = 0.99$	7.88 %	|3.68| %
P21-P26	0.266	5	0.278 ± 0.135	48.6 %	4.6 %
	0.665	5	0.684 ± 0.176	25.8 %	2.8 %
	1.33	4	1.16 ± 0.245	21 %	-12.5 %
Summary$^\flat$			$R^2 = 0.98$	31.8 %	|6.63| %
All$^\flat$			$R^2 = 0.985$	20.5 %	|10.8| %

* Pores were grouped together by a common detection reagent, and in the cases of pores P21-P26 by the presence of saliva background.

† m is the number of target estimates $[B]_0^e$ at a common $[B]_0$ used to compute the mean and standard deviation.

♭ $R^2 = 1 - \sum(x-y)^2 / \sum(x - \text{mean}(x))^2$ where x is the vector of the base-10 logarithm of the mean estimates and y is the base-10 logarithm of the corresponding knowns $[B]_0$. Also reported are the mean CV and the mean of the absolute PE values.

The USDA "Guidance for Industry: Bioanalytical Method Validation" documentation [17] states an accuracy and precision standard of 15% PE and CV, respectively, using a minimum of 5 replicate estimates per known concentration. Only three of the combined estimates in Table S45 have $m = 5$ estimates. Among those the mean $|\text{PE}| = 4.9\%$ is well below the 15% requirement, while the mean CV = 27% is too high. We expect both the accuracy and precision to improve when m single-pore estimates can be aggregated from a single experiment using an m-arrayed nanopore test strip. The mean estimate that results from the m-arrayed device will then constitute a single "measurement," and replicate experiments will generate 5 or more of such measurements that are averaged to test CV and PE performance. In that format, the m-pore reproducibility can also be tested, e.g., across different days and users, etc. Of the stated four conditions affecting reproducibility of the presented results, only condition 3 (variation in nanopore sizes) would persist in generating a single estimate "measurement" with an arrayed nanopore test strip. Note that the combined estimates from P14 and P15 used aliquots from the same pool of reagents, and despite having two different experimenters, showed accuracy and precision performance well below the 15% requirement.

Table S46 results with Q data and the 2 parameter model and Table S47 results with R_T data and the 3 parameter model show comparable mean CV and PE performance, across all data sets. The interesting result from Table S47 is how good the performance is for the longer scaffold (P7-P13), with the mean CV and PE below the 15% requirement. Further testing with samples will have to determine if this is a persistent trend. It could be related to the fact that 1074 bp DNA is a more resolvable molecule than 217 bp DNA in larger nanopores, and so the capture rate is a more reliable metric for the longer scaffold detection reagent. The increase in mean CV with sample background (comparing 38% without saliva P14-P15, with 64% with saliva P21-P26) suggests that the presence of sample can undermine quantitation based on the capture rate. Lastly, Table S48 with R_T data and the 2 parameter model shows the lowest performance.

Table S46: **Combined target concentration estimates: 2 param model, Q data**

Pore IDs*	$[B]_0$ (nM)	m†	Mean ± SD $[B]_0^e$ (nM)	CV	PE
P7-P13	0.1	2	0.106 ± 0.0187	17.6 %	6.42 %
	0.2	2	0.159 ± 0.0477	30 %	-20.6 %
	0.5	4	0.335 ± 0.342	102 %	-33 %
	1	5	0.74 ± 0.251	33.9 %	-26 %
	2.5	4	3.05 ± 1.09	35.6 %	22.2 %
Summary♭			$R^2 = 0.951$	43.8 %	|21.7| %
P14-P15	1.4	2	1.04 ± 0.256	24.5 %	-25.4 %
	2.8	2	3.38 ± 0.413	12.2 %	20.8 %
Summary♭			$R^2 = 0.82$	18.4 %	|23.1| %
P21-P26	0.266	4	0.354 ± 0.232	65.7 %	33 %
	0.665	3	0.627 ± 0.101	16 %	-5.77 %
	1.33	3	1.3 ± 0.338	26.1 %	-2.36 %
Summary♭			$R^2 = 0.90$	35.9 %	|13.7| %
All♭			$R^2 = 0.953$	36.4 %	|19.6| %

* Pores were grouped together by a common detection reagent, and in the cases of pores P21-P26 by the presence of saliva background.

† m is the number of target estimates $[B]_0^e$ at a common $[B]_0$ used to compute the mean and standard deviation.

♭ $R^2 = 1 - \sum(x-y)^2 / \sum(x - \text{mean}(x))^2$ where x is the vector of the base-10 logarithm of the mean estimates and y is the base-10 logarithm of the corresponding knowns $[B]_0$. Also reported are the mean CV and the mean of the absolute PE values.

Table S47: **Combined target concentration estimates: 3 param model, R_T data**

Pore IDs*	$[B]_0$ (nM)	m†	Mean ± SD $[B]_0^e$ (nM)	CV	PE
P7-P13	0.1	2	0.113 ± 0.00319	2.83 %	13 %
	0.2	2	0.199 ± 0.0151	7.58 %	-0.37 %
	0.5	4	0.603 ± 0.17	28.2 %	20.6 %
	1	5	1.04 ± 0.17	16.4 %	3.59 %
	2.5	3	2.32 ± 0.224	9.65 %	-7.09 %
	5	2	5.75 ± 1.07	18.5 %	15.1 %
Summary♭			$R^2 = 0.993$	13.9 %	|9.96| %
P14-P15	1.4	2	1.71 ± 0.437	25.6 %	22 %
	5.6	2	3.08 ± 1.54	50.2 %	-45.1 %
Summary♭			$R^2 = -1.3$	37.9 %	|33.5| %
P21-P26	0.266	5	0.31 ± 0.173	55.8 %	16.5 %
	0.665	4	0.86 ± 0.357	41.5 %	29.3 %
	1.33	3	1.72 ± 0.99	57.5 %	29.6 %
	2.66	3	2.03 ± 1.11	54.4 %	-23.6 %
	13.3	2	7.46 ± 8.29	111 %	-43.9 %
Summary♭			$R^2 = 0.897$	64.1 %	|28.6| %
All♭			$R^2 = 0.945$	36.9 %	|20.8| %

* Pores were grouped together by a common detection reagent, and in the cases of pores P21-P26 by the presence of saliva background.

† m is the number of target estimates $[B]_0^e$ at a common $[B]_0$ used to compute the mean and standard deviation.

♭ $R^2 = 1 - \sum(x-y)^2 / \sum(x - \text{mean}(x))^2$ where x is the vector of the base-10 logarithm of the mean estimates and y is the base-10 logarithm of the corresponding knowns $[B]_0$. Also reported are the mean CV and the mean of the absolute PE values.

Table S48: **Combined target concentration estimates: 2 param model, R_T data**

Pore IDs*	$[B]_0$ (nM)	$m^\dagger$	Mean ± SD $[B]_0^e$ (nM)	CV	PE
P7-P13	0.1	2	0.106 ± 0.0294	27.7 %	5.94 %
	0.2	2	0.19 ± 0.0303	16 %	-5.15 %
	0.5	4	0.371 ± 0.153	41.3 %	-25.7 %
	1	5	0.86 ± 0.267	31.1 %	-14 %
	2.5	3	2.21 ± 0.446	20.1 %	-11.4 %
	5	2	5.8 ± 1.12	19.2 %	16 %
Summary♭			$R^2 = 0.987$	25.9 %	|13.0| %
P14-P15	5.6	2	3.02 ± 1.05	34.9 %	-46.1 %
Summary♭			R^2 na	34.9 %	|46.1| %
P21-P26	0.266	4	0.366 ± 0.231	63.1 %	37.7 %
	0.665	2	1.05 ± 0.223	21.3 %	58 %
	1.33	2	2.1 ± 1.01	48 %	57.8 %
	2.66	3	2 ± 1	49.9 %	-24.7 %
	6.65	2	2.43 ± 1.84	75.5 %	-63.4 %
	13.3	2	13.8 ± 0.678	4.91 %	3.91 %
Summary♭			$R^2 = 0.77$	40.9 %	|43.8| %
All♭			$R^2 = 0.906$	34.8 %	|28.4| %

* Pores were grouped together by a common detection reagent, and in the cases of pores P21-P26 by the presence of saliva background.

† m is the number of target estimates $[B]_0^e$ at a common $[B]_0$ used to compute the mean and standard deviation.

♭ $R^2 = 1 - \sum(x-y)^2 / \sum(x - \text{mean}(x))^2$ where x is the vector of the base-10 logarithm of the mean estimates and y is the base-10 logarithm of the corresponding knowns $[B]_0$. Also reported are the mean CV and the mean of the absolute PE values.

Figure S24 shows the mean and standard deviation $[B]_0^e$ values reported in Tables S45-S48. The R^2 metric is used to show how closely the average estimates are to the zero error line. The plots reveal the same trends as the tables, namely, the (Q data, 3 parameter model) estimates are the closest to the zero line and with the least spread around the mean values.

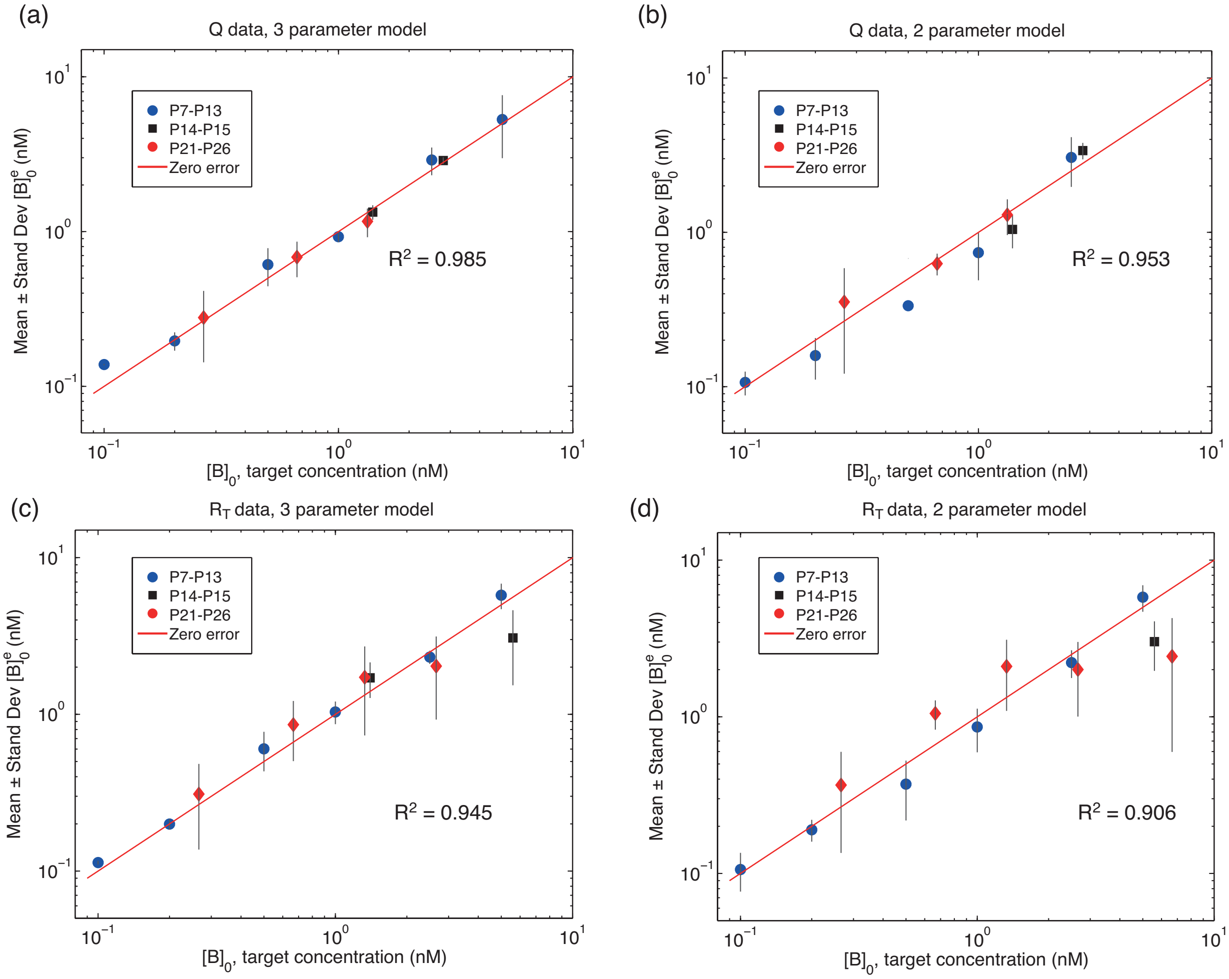

Figure S24: Mean and standard deviation of repeated $[B]_0^e$ estimates vs. $[B]_0$. The zero error (PE = 0) line is plotted for comparison. The plots are grouped by model order and data type: (a) 3 parameter, Q data; (b) 2 parameter, Q data; (c) 3 parameter, R_T data; (d) 2 parameter, R_T data.

A preliminary assessment of combining target estimates using the two-stage approach applied to Q data is presented in Table S49. The data were chosen from the saliva sample background group (P21-P26) where at least 3 estimates were available for averaging. The resulting PE and CV performance is promising, suggesting that further testing of this approach is worthwhile. As stated, the approach reduces the number of controls needed for quantitation to one. This reduction in turn reduces the total time to quantitative results, and would simplify the microfluidics requirements for an integrated sample-to-answer format where quantitation is required.

Table S49: **Combined two-stage estimates using Q data (P21-P26)**[*]

$X \rightarrow X + Y$	$[B]_0$ (nM)	$m^\dagger$	Mean ± SD $[B]_0^e$ (nM)	CV	PE
0.5 → 2	0.665	3	0.621 ± 0.083	13.4 %	-6.57 %
0.5 → 5	0.665	3	0.801 ± 0.415	51.8 %	20.5 %
0.5 → 10	0.665	3	0.645 ± 0.115	17.9 %	-3.01 %
1 → 5	1.33	4	1.33 ± 0.308	23.1 %	0.169 %
Summary[♭]				26.6 %	|7.56| %

[*] A subset of at least three of the six pores (P21-P26) had an estimate available for each row.

[†] m is the number of target estimates $[B]_0^e$ at a common $[B]_0$ used to compute the mean and standard deviation.

[♭] Reported are the mean CV and the mean of the absolute PE values.

12.3 Exploring detection limits (P27, P28) and binding affinity

In two parallel nanopore experiments, the detection reagent with 1074 bp DNA scaffold was incubated at 1.3 nM with varying amounts of HGD65 Ab, from 2X down to 0.05X. After 5 minutes of incubation, each reagent was diluted 1.3X into nanopore recording buffer to the concentrations shown in Table S50. The tabulated results suggests that 2.6 nM Ab at incubation is near the limit of reliable detection in buffer only. In the presence of 10% saliva, 2.66 nM Ab with 13.3 nM detection reagent at incubation was also detectable (P23, P25, and P26) after 10X dilution. Other experiments in buffer only (P7, P8) show that 5 nM Ab and 20 nM detection reagent can be incubated and then diluted 50-fold to 0.1 nM Ab and 0.4 nM detection reagent, and still show positive detection. The apparent lower limit of ∼2 nM Ab at incubation is likely driven by the affinity of the target for the binding domain (V3 peptide) of the detection reagent.

In consideration of the limit of detection of the instrument [18], the lowest concentration detected without optimization was 0.1 nM HGD65 in buffer only (P7, P8), which registered a Q with 95% CI that exceeded the Q with 95% CI for the corresponding set of negatives run on each pore. More precisely, the P7 and P8 negatives collectively registered a Q with 95% CI at 0.05 or lower, while the 0.1 nM Ab results had a Q (minus 95% CI) above 0.05. By the same logic, the limit of detection of the saliva assay was 0.266 nM HGD65 in 10% saliva (P21-P23, P25, P26).

We consider K_d estimation also. In the 2 parameter model, the K_d parameter is fit to the transition portion of the Q data, i.e., between $X = 0$ and where Q approaches saturation. The following experiments produced a fitted K_d estimate with a CV (standard deviation divided by mean) below 100% using the 2 parameter model and Q data: for 1074 bp scaffold, P8 and P10-P13; for 217 bp scaffold, P14-P16, P18, P21 and P24-P26. Combining these produces $K_d = 0.86 \pm 0.58$ nM for the 1074 bp scaffold and $K_d = 0.24 \pm 0.25$ nM for the 217 bp scaffold. Averaging all values regardless of scaffold produces $K_d = 0.48 \pm 0.5$ nM. An ELISA run on a plate coated with PNA[1]-peptide (Sec. 10.1.2), the bi-functional fusion molecule used in nanopore experiments, resulted in dissociation constant estimates of $K_d = 0.4 \pm 0.2$ nM for HGD65 and $K_d = 6.2 \pm 0.9$ nM for 447-52D. An ELISA run on a plate coated with the V3 peptide alone (i.e., without the PNA) resulted in an estimate of $K_d = 2.2 \pm 1.1$ nM for HGD65.

Table S50: **HGD65 detection limit experiments using test strips/MOM**

Pore ID, Diam., Setup	Reagents & Concentrations*	Ratio[†] X	Tagged % [‡] (99% CI)	No. Events	Rec. Time	TTP 99%[♮]
P27	1. DNA^a 1 nM	–	0.104 ± 0.27%	965	5 min	np
31-35 nm	2. DNA^a/PNA-pep 1 nM	0	2.02 ± 0.88%	1680	10 min	np
Test Strip	3. DNA^a/PNA-pep 1 nM + Ab 2 nM	2	16.4 ± 3.9%	605	10 min	1.5 min
MOM	4. DNA^a/PNA-pep 1 nM + Ab 1 nM	1	2.16 ± 0.86%	1896	10 min	np
	5. DNA^a/PNA-pep 1 nM + Ab 0.5 nM	0.5	0.994 ± 0.58%	1911	10 min	np
	6. DNA^a/PNA-pep 1 nM + Ab 0.1 nM	0.1	2.05 ± 0.91%	1607	10 min	np
	7. DNA^a/PNA-pep 1 nM + Ab 0.05 nM	0.05	1.07 ± 0.6%	1971	10 min	np
	8. DNA^a/PNA-pep 1 nM	0	0.963 ± 0.49%	2595	10 min	np
P28	1. DNA^a 1 nM	–	0.147 ± 0.38%	679	5 min	np
29-31 nm	2. DNA^a/PNA-pep 1 nM	0	2 ± 0.83%	1899	11 min	np
Test Strip	3. DNA^a/PNA-pep 1 nM + Ab 2 nM	2	24 ± 3%	1343	10 min	22 sec
MOM	4. DNA^a/PNA-pep 1 nM + Ab 1 nM	1	1.82 ± 0.71%	2360	11 min	np
	5. DNA^a/PNA-pep 1 nM + Ab 0.5 nM	0.5	1.36 ± 0.67%	1992	10 min	np
	6. DNA^a/PNA-pep 1 nM + Ab 0.1 nM	0.1	1.09 ± 0.61%	1927	10 min	np
	7. DNA^a/PNA-pep 1 nM + Ab 0.05 nM	0.05	0.5 ± 0.41%	2001	10 min	np
	8. DNA^a/PNA-pep 1 nM	0	1.21 ± 0.63%	1979	11 min	np

[*] Experiments were conducted at buffer pH 8.8, 1M LiCl, 10 mM Tris, 1 mM EDTA, and 100 mV.

[†] X is the dimensionless target to scaffold/fusion concentration ratio, defined only when the complete detection reagent (scaffold/fusion) competent to bind target is present.

[‡] Events are tagged positive if: max $\delta G > 3.31$ nS for P27, and max $\delta G > 2.84$ nS for P28. The reported % are $Q \pm Q_{\mathrm{conf}}$ as defined in Section 6.

[♮] TTP (time-to-positive) is the first time at which the tagged percentage minus the 99% CI is greater than, and remains greater than, a false-positive threshold of 2%, as detailed in Section 6. The "np" entry for each reagent set reflects when this criteria is not satisfied.

[a-c] DNA^a = 1074 bp, DNA^b = 217 bp, DNA^c = 108 bp.

12.4 Detection and quantitation ranges

The presented results showcase our methods using HIV Ab target as a model, without optimizing performance for clinical or commercial relevance. In any case, the detection and quantitation ranges can be compared to those ranges expected to occur in actual samples. The HGD65 Ab is 150 kDa, and 1 nM is equivalent to 0.15 mg/liter. Three pores (P23, P25, and P26) generated the largest single-nanopore range of HGD65 Ab concentration that was positively detected. The range was 50-fold, specifically 2.66-133 nM (0.4-20 mg/liter) at incubation in 10% saliva. Accounting for the 10-fold dilution, the demonstrated detectable range in 100% saliva sample would be 4-200 mg/liter. Prior work states that IgG concentrations in salivary specimens present at 0.5-100+ mg/liter in positive samples [19, 20]. Conceivably, the 0.5 mg/limit could be achieved with a single nanopore assay. This could be tested by spiking 0.4 mg/liter into 80% saliva with 5-fold more detection reagent, followed by 20X dilution into recording buffer. The resulting 4% saliva background is dilute enough that it is unlikely to clog the pore, while the 0.133 nM Ab above the pore is higher than the 0.1 nM Ab already shown detectable (P7, P8). In any case, the demonstrated range already substantially overlaps with the clinically relevant range and without optimization. No upper limit on the detectable range was identified in this study, and in principle increasing amounts of Ab can be accommodated as needed by incubation with a higher concentration of detection reagent, followed by higher dilution to the appropriate concentration for efficient nanopore detection. The demonstrated low end of detection was 2.6 nM Ab (0.39 mg/liter) at incubation as discussed in section 12.3.

Table S51 provides a summary of the detection range and quantitation range, precision and accuracy. The nanopores are grouped together by buffer only, saliva and serum background. The quantitative range is narrower than the detection range, only because quantitated values required replicate estimates in order to also assess precision and accuracy. Note that experiments were not designed to demonstrate the broadest possible range, for detection or quantitation; rather, the results are intended to present the efficacy of the method. The results summarized in Table S51 show a baseline of performance, and enhancements in all categories are anticipated by used arrays of pores in the consumable.

Table S51: **Summary of HGD65 Ab Detection and Quantitation Performance**

Performance Criteria	Buffer only	Spiked Saliva (10%)	Spiked Serum (10%)
Detection Range†	0.39-75 mg/liter	0.4-20 mg/liter	5.25-111 mg/liter
Quantitation Range‡	0.75-30 mg/liter	0.4-2 mg/liter	10-21 mg/liter♯
Precision	mean CV = 16 %	mean CV = 32 %	n/a
Accuracy	mean |PE| = 12 %	mean |PE| = 7 %	n/a

† Defined as the total range of HGD65 Ab concentration at incubation across subsets of nanopore results as follows: no sample, 13 pores are P7-P15, P27-P28; saliva, 6 pores are P21-P26; serum, 3 pores are P18-P20. HGD65 Ab is 150 kDa, and 1 nM is equivalent to 0.15 mg/liter. Post-incubation dilutions for nanopore recording: no sample, 12.5-50X; saliva, 10X; serum, 25X.

‡ Quantitative range, precision and accuracy values are derived from the combined single-pore estimates reported in Table S45 for the buffer only and spiked saliva results. Range is after converting to incubation concentrations. Nanopore IDs: no sample, 11 pores are P7-P15; saliva, 6 pores are P21-P26.

♯ Only one pore (P18) out of the three was tested with enough target concentrations to generate quantitative data (Table S28). Without replicate estimates at each target concentrations, CV and PE values are not available (n/a).

12.5 Time-to-positive and sample-to-result times

The time to positive (TTP) versus target concentration above the pore $[B]_0$ is plotted in Figure S25a for each reagent of pores P7-P26. For each $[B]_0$ measured at least twice, the average TTP is computed plotted in Figure S25b. In most cases the incubation time was 5-15 minutes, longer than nearly all TTP value across the demonstrated range.

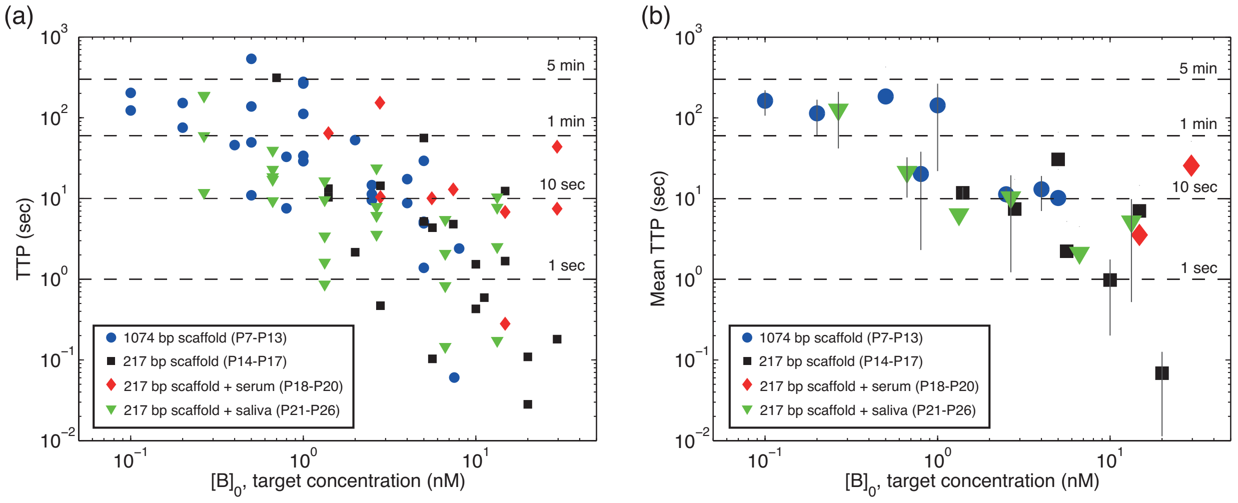

Figure S25: Single nanopore sensing time-to-positive (TTP) trends vs. Ab concentration $[B]_0$ above the pore. (a) Data points are the TTP values reported in the tables for the corresponding pore and reagent $[B]_0$ concentration. (b) Data points are the mean TTP values when at least two values were measured at the corresponding $[B]_0$, using the same scaffold and sample type.

Sample-to-results time can also be examined in the context of the Ab detection results in the presence of saliva. Only in the venue of background saliva did we attempt to reduce the incubation period prior to nanopore sensing. Figure S26 shows the sample-to-positive times for six nanopores, pores P21-P24 using a 5 minute incubation and pore P25-P26 using a 30 second incubation prior to sensing. The sample-to-positive time is the sum of the incubation period plus the TTP value (Fig. S25), where TTP requires positive detection with 99% CI. The pipetting and liquid transfer times, between incubation and dilution tubes and then into test strip channel, were negligible and not included. Even at 30 seconds incubation, the incubation period was longer than the sensing period required with a single nanopore to positively detect 1-200 mg/liter Ab with 99% confidence. Only at the lowest concentration considered (0.4 mg/liter) did the sensing time exceed the incubation period. An advantage of nanopore assays is that target binding kinetics occur in the bulk phase, rather than on a coated 2D surface or sensor. As a corollary, the nanopore does not require pressure driven flow to promote sufficient target/binding-domain interaction, therefore reducing the design complexity and cost of the sample-to-answer consumable.

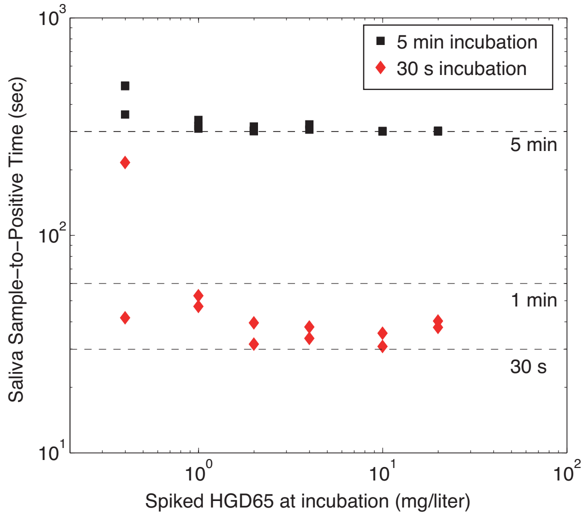

Figure S26: Sample-to-positive time vs. target concentration spiked into 10% saliva at incubation. Four nanopores (P21-P24) used a 5 minute incubation period and two nanopore (P25-P26) used a 30 second incubation period prior to dilution into the nanopore sensing channel. The plotted sample-to-positive time is the sum of the incubation period plus the time-to-positive (TTP) value reported in the tables for these nanopores.

13 Detailed results with TNFα

13.1 Methods

13.1.1 Construction and verification of TNFα detection reagent

An affibody molecule against human TNFα was purchased directly from the manufacturer (Affibody AB, Catalog No. 10.084101.0050). The affibody contains a C-terminal cysteine whose sulfhydryl group was used to conjugate to the N-terminus of a bis-peptide nucleic acid (bisPNA) of the following sequence via an intermediate succinimidyl 4-(N-maleimidomethyl)cyclohexane-1-carboxylate (SMCC) linker:

KK-O-TCCCCTCCTTTT-O-O-O-TTTTJJTJJJJT-O-KK

where K is the amino acid lysine, and O represents a 2-aminoethoxy-2-ethoxy acetic acid (AEEA) linker.

In order to form a dsDNA-PNA-affibody complex, the purified affibody-bisPNA conjugate was allowed to incubate with a 309bp dsDNA fragment containing the complementary sequence to the Watson-Crick strand of the bisPNA at a 60-fold molar excess for 2 hours at 60C. (10mM sodium phosphate, pH 7.4). Excess bisPNA-affibody was cleaned up by centrifugation in a 100kDa filter (EMD Millipore, UFC570024). Successful invasion of the bisPNA-affibody onto dsDNA was verified via an electrophoretic mobility shift assay (EMSA) on a 6% TBE gel (ThermoFisher, EC62652) at 200V for 40 minutes.

Following purification of the binding reagent, it was allowed to incubate with a titration series of human TNFα (ThermoFisher, PHC3016) for a period of 1 hour at room temperature (10mM sodium phosphate, pH 7.4). Confirmation of TNFα binding was conducted on a similarly run TBE gel. Figure S27 shows verification of TNFα binding to the scaffold/bisPNA-affibody reagent. As protein concentration increases, upper bands appear that are indicative of successful TNFα capture by the affibody binding reagent. The multiple bands that form are a consequence of the various invasion complexes formed by bisPNA on dsDNA [2], as well as the multimeric nature of TNFα. Following the incubation period between 50 nM scaffold/fusion and each TNFα concentration, the reagents were diluted 16.7-fold into nanopore buffer for experimentation.

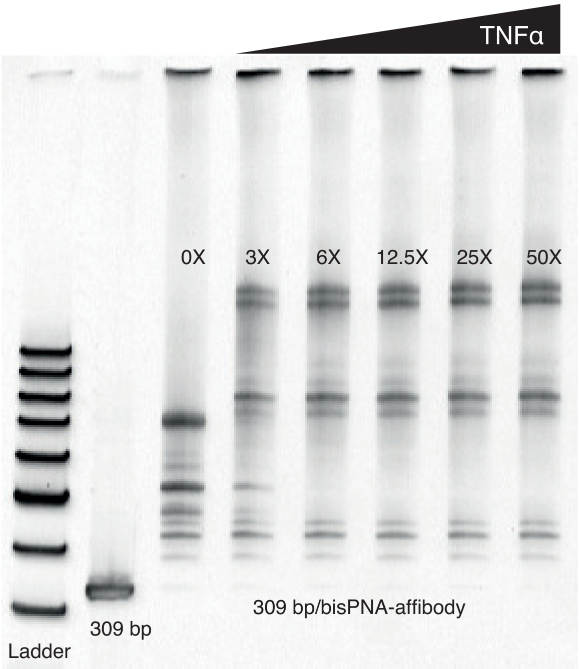

Figure S27: EMSA verification of TNFα binding to the scaffold/bisPNA-affibody reagent. Band intensity appears to saturate at 3X and higher ratios of TNFα to scaffold/bisPNA-affibody.

13.1.2 TNFα sandwich ELISA

TNFα specific affibody was coated at 250ng/well in a Nunc MaxiSorp 96-well plate (ThermoFisher, Cat. No. 442404) overnight at 4C. Subsequent to coating with affibody, the plates were blocked with 1% BSA in PBS for 2 hours at room temperature. The plates were then washed with PBS, and human TNFα (ThermoFisher, Cat. No. PHC3011) was titrated 1:1 across the plate in duplicate and allowed to incubate at room temperature for a period of 1.5 hours. A biotin-conjugated TNFα detection antibody (ThermoFisher, Cat. No. KHC3011) was added to the plate at a 1:2000 dilution in antibody diluent (PBS containing 0.2% BSA), and incubated at room temperature for 2 hours. For plate visualization, streptavidin-HRP was added, washed, and incubated with 100uL TMB chromagen/substrate for a period of 30 minutes (ThermoFisher, Cat. No. SB01). The enzymatic reaction was stopped using 100uL of stop solution (ThermoFisher, Cat. No. SS03100), and the plates were read at 450nm in a Victor2 1420 microplate reader. The resulting data was analyzed using GraphPad Prism 7 Software. The average predicted affinity K_d on two different days were 0.26 nM and 0.08 nM, each with estimate CV = 20%. Another ELISA tested the ability of the TNFα-to-affibody interaction to withstand nanopore running buffer (1M LiCl), and no difference in binding trends was observed compared to the standard TNFα-to-affibody binding buffers. This suggests that the nanopore high salt buffer does not interfere with the interaction between TNFα and affibody.

13.2 Pores P29-P31: Detection and quantitation

Experiments tested scaffold and scaffold/fusion without and with TNFα, with two pores (P29, P30) using standard holders and the 700B amplifier and one pore (P31) using a test strip and MOM. The results are in Table S52.

Table S52: **Detection and time-to-positive data for TNFα**

Pore ID, Diam. & Setup	Reagents & Concentrations*	Ratio† X	Tagged % ‡ (99% CI)	No. Events	Rec. Time	TTP 99%♮
P29	1. DNA^d 10 nM	–	0 ± 0%	392	16 min	np
25-30 nm	2. DNA^d/PNA-aff 3 nM	0	1.93 ± 1.6%	518	10 min	np
Holder	3. TNFα 37.5 nM	–	14.3 ± 34%	7	5 min	np
700B	4. DNA^d/PNA-aff 3 nM + TNFα 37.5 nM	12.5	33.1 ± 3.5%	1222	10 min	5.6 sec
	5. DNA^d/PNA-aff 3 nM + TNFα 18 nM	6	31 ± 3.3%	1336	10 min	5.3 sec
	6. DNA^d/PNA-aff 3 nM + TNFα 9 nM	3	27.5 ± 2.9%	1565	10 min	10 sec
	7. DNA^d/PNA-aff 3 nM + TNFα 3 nM	1	9.04 ± 2.6%	819	10 min	41 sec
	8. DNA^d/PNA-aff 3 nM + TNFα 1.5 nM	0.5	5.43 ± 3.1%	350	4 min	2.3 min
	9. DNA^d/PNA-aff 3 nM + TNFα 0.75 nM	0.25	1.59 ± 1.2%	690	10 min	np
	10. DNA^d 10 nM	–	0.568 ± 1.5%	176	21 min	np
P30	1. DNA^d 5 nM	–	0.278 ± 0.51%	719	11 min	np
18-23 nm	2. DNA^d/PNA-aff 3 nM	0	2.05 ± 1.7%	439	23 min	np
Holder	3. TNFα 37.5 nM	–	0 ± 0%	13	6 min	np
700B	4. DNA^d/PNA-aff 3 nM + TNFα 37.5 nM	12.5	35.5 ± 3.7%	1082	11 min	4.1 sec
	5. DNA^d/PNA-aff 3 nM + TNFα 18 nM	6	33.7 ± 4.8%	655	21 min	22 sec
	6. DNA^d/PNA-aff 3 nM + TNFα 9 nM	3	26.4 ± 4.6%	618	21 min	1 min
	7. DNA^d/PNA-aff 3 nM + TNFα 3 nM	1	10.3 ± 3.5%	513	21 min	31 sec
	8. DNA^d/PNA-aff 3 nM + TNFα 1.5 nM	0.5	4.45 ± 2.1%	651	16 min	13 min
	9. DNA^d/PNA-aff 3 nM + TNFα 0.75 nM	0.25	1.92 ± 1.5%	572	11 min	np
	10. DNA^d 5 nM	–	0.279 ± 0.51%	717	11 min	np
P31	1. DNA^d/PNA-aff 3 nM	0	2.03 ± 1.6%	542	12 min	np
27-31 nm	2. TNFα 37.5 nM	–	0 ± 0%	2	2 min	np
Test strip	3. DNA^d/PNA-aff 3 nM + TNFα 37.5 nM	12.5	13.9 ± 3.5%	656	7 min	42 sec
MOM	4. DNA^d/PNA-aff 3 nM + TNFα 18 nM	6	14.9 ± 3.4%	725	11 min	46 sec
	5. DNA^d/PNA-aff 3 nM + TNFα 9 nM	3	17.2 ± 4%	600	4 min	9.8 sec
	6. DNA^d/PNA-aff 3 nM + TNFα 3 nM	1	11.2 ± 3.4%	580	9 min	1.4 min
	7. DNA^d/PNA-aff 3 nM + TNFα 1.5 nM	0.5	5.11 ± 2.6%	470	9 min	5.1 min
	8. DNA^d/PNA-aff 3 nM	0	0 ± 0%	23	4 min	np

* Experiments were conducted at buffer pH 8.8, 1M LiCl, 10 mM Tris, 1 mM EDTA, and 100 mV.

† X is the dimensionless target to scaffold/fusion concentration ratio, defined only when the complete detection reagent (scaffold/fusion) competent to bind target is present.

‡ Events are tagged positive if: max $\delta G > 3.56$ nS for P29; max $\delta G > 4.1$ nS for P8; max $\delta G > 3.9$ nS for P31. The reported % are $Q \pm Q_{\text{conf}}$ as defined in Section 6.

♮ TTP (time-to-positive) is the first time at which the tagged percentage minus the 99% CI is greater than, and remains greater than, a false-positive threshold of 2%, as detailed in Section 6. The "np" entry for each reagent set reflects when this criteria is not satisfied.

^{d} DNA^d = 309 bp. PNA-aff is the bisPNA-affibody construct defined in the Methods section.

The same reagents were tested on each pore by three different experimenters. Reagents were also tested biochemically using EMSA assays prior to measurement on the nanopore (Fig. S27). All three pores showed saturation in Q for $X \geq 3$. The test strip nanopore performance was not as good as with holders, and was also not as good as average test strip pores. Specifically, the signal RMS at 10 kHz bandwidth was 24 pA, almost twice the typical 13 pA RMS (Table S2) for test strips. Despite the higher noise of P31, all three pores showed positive detection of TNFα concentrations at 1.5 nM and higher.

The all event scatter plot and max δG histogram for pore P29 are shown in Figure S28. The tagged events are not as deep or as long in duration as the tagged events for the HIV Ab assay. One reason is the size of TNFα is roughly one-third the size of HIV Ab. An assay using a secondary anti-TNFα antibody to bind and bulk-up the full complex could enhance the detectable signal for TNFα-bound complexes, and therefore improve the detection performance of the assay.

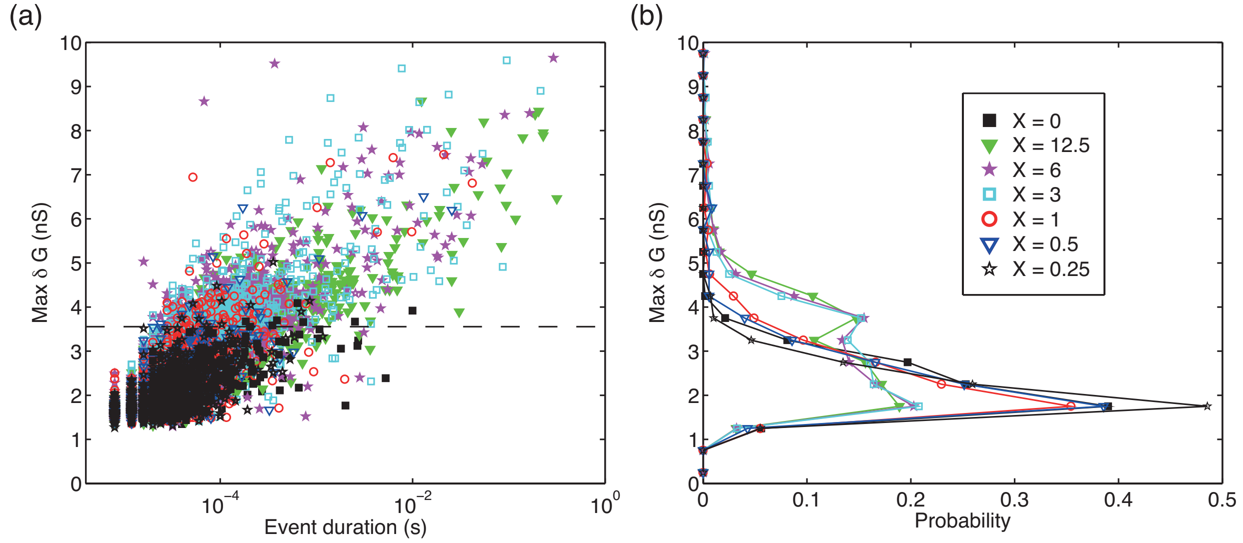

Figure S28: Pore P29 experiment with titration of TNFα. (a) All-event scatter plots of max δG vs. duration and (b) the corresponding histograms for max δG, varying the target to scaffold/fusion concentration ratio X. The horizontal line in (a) shows the max δG threshold above which events are tagged positive.

The tagged fraction model fitting performance is reported in Table S53. For the small data set size considered in this preliminary study, we looked only at modeling the Q data with the two and three parameter models. In all three cases, the three parameter model fits the data better. Figure S29 shows the two and three parameter model fits and the data.

Table S53: **Fitted parameter values from modeling tagged fraction**

Pore ID	3 parameter model equation (3)				2 parameter model equation (4)		
	α	K_d (nM)	q_1	NRMS	α	K_d (nM)	NRMS
P29	0.35 ± 0.016	0.34 ± 0.23	0.17 ± 0.072	3.8 %	0.35 ± 0.022	2.4 ± 0.95	12 %
P30	0.39 ± 0.023	0.57 ± 0.62	0.2 ± 0.091	1.7 %	0.39 ± 0.026	3.3 ± 1.4	9.6 %
P31	0.15 ± 0.0086	0.072 ± 0.12	0.51 ± 0.22	9.8 %	0.16 ± 0.01	0.42 ± 0.46	13 %

* Parameters and normalized root-mean-square error (NRMS) are defined in section 7.1. The "NaN" value is reported when the model fails to fit the data.

♭ MD = mean data. The model fitting is applied to the averaged Q values and error bars, which are reported in the main text.

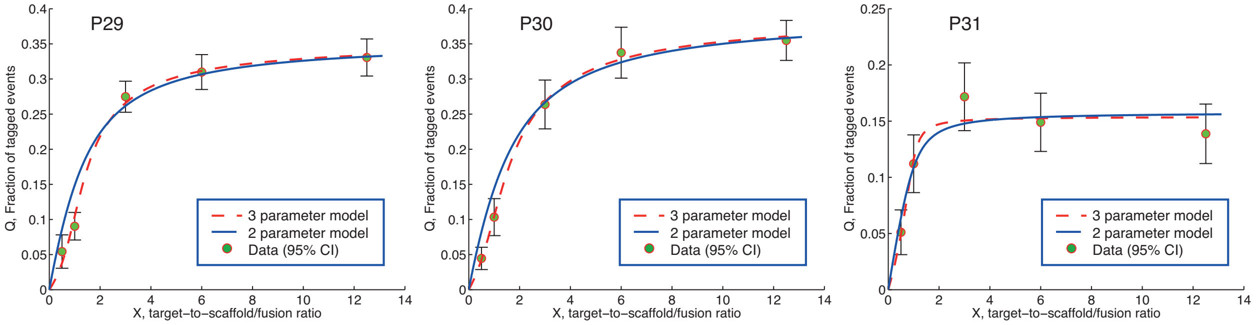

Figure S29: Data (95% CI) with two and three parameter model fit curves of tagged fraction Q vs. X for pores P29-P31.

Two separate ELISAs predicted K_d of 0.26 nM and 0.08 nM, each with estimate CV = 20%, for the same affibody and TNFα reagents used in our nanopore workflow. As previously discussed, the two and three parameter models derived here are phenomenological and the K_d parameter may or may not reflect the true bulk-phase binding constant. In any case, the three parameter model fit produces K_d estimates comparable to the ELISA values, though with comparatively higher CV for each estimate.

The target concentration estimates are reported in Table S54. Over the 1-log range considered, the quantitative performance is comparable to that achieved for the HIV Ab target. Table S55 shows the performance when combining the estimates reported in Table S54, achieving average precision and accuracy values below 10%.

Table S54: **Target concentration estimates using tagged fraction models**

Pore ID	Ratio X	$[B]_0$ (nM)	3 parameter model equation (5)			2 parameter model equation (6)		
			$[B]_0^e$ (nM)	CV	PE	$[B]_0^e$ (nM)	CV	PE
P29	0.5	1.5	1.9 ± 0.38	19 %	29 %	0.9 ± 0.26	29 %	-40 %
	1	3	2.7 ± 0.29	10 %	-9.3 %	1.6 ± 0.3	19 %	-47 %
	3	9	10 ± 2	20 %	11 %	11 ± 2.3	21 %	18 %
P30	0.5	1.5	1.6 ± 0.32	21 %	3.4 %	0.76 ± 0.2	26 %	-49 %
	1	3	3 ± 0.37	12 %	-1 %	2 ± 0.47	24 %	-35 %
	3	9	8.7 ± 1.9	22 %	-3 %	8.7 ± 2.2	26 %	-2.9 %
P31	0.5	1.5	1.5 ± 0.36	23 %	2.8 %	1.2 ± 0.28	24 %	-22 %
	1	3	2.9 ± NaN	–	-3.6 %	3.2 ± 1	33 %	5.4 %

* CV (coefficient of variation) is the standard deviation divided by the mean for $[B]_0^e$ converted to a percentage, and PE (percentage error) = $100 \times ([B]_0^e - [B]_0)/[B]_0$. The "na" is reported when the estimate has a CV of 100% or greater.

♭ MD = mean data. The predicted values are based on the model fitted to the averaged Q values, reported in Table S38.

Table S55: **Combined estimates for P29-P30 (3 param model, Q data)**

$[B]_0$ (nM)	m†	Mean ± SD for $[B]_0^e$ (nM)	CV	PE
1.5	3	1.67 ± 0.022	13.3 %	11.6 %
3	2	2.86 ± 0.128	4.46 %	-4.67 %
9	2	9.35 ± 0.87	9.31 %	3.83 %

* Estimates generated by combining values from Table S54.

† m is the number of target estimates $[B]_0^e$ at a common $[B]_0$ used to compute the mean and standard deviation.

13.3 Range and detection limits without optimization

Accounting for the dilution factor, 1.5-37.5 nM on the pore corresponds to 25-625 nM at incubation. The TNFα protein is 52 kDa, and 1 nM is equivalent to 0.052 mg/liter. Converting units, the detected concentration range is 78-1950 μg/liter on the pore and 1.3-32.5 mg/liter at incubation.

TNFα is a cell signaling protein involved in systemic inflammation. In one representative study [21], for example, a relationship between elevated Interleukin-6 and TNFα levels and metastatic prostate cancer was examined, finding that "...both cytokines correlate with the extent of disease and should be monitored in conjunction with other disease indicators (PSA)." The authors cite that the elevated threshold is 1.9 ng/liter, and a high sensitivity ELISA can detect TNFα as low as 0.09 ng/liter. The lower value on the pore (78μg/liter) is 4-log higher than the 1.9 ng/liter limit needed to detect elevated levels. Detection sensitivity can be optimized by incorporating a scaffold/fusion/target concentration step upstream of nanopore sensing.

By comparison, high sensitivity ELISA with an automated workflow has been realized commercially, but it is expensive in time and cost. The Quanterix Simoa tech (digital ELISA) [22] [23] achieves 3 ng/liter for TNFα. Total assay time is 6 hours, including 30-45 min incubation steps interlaced with 3 to 6-times bead washing steps, with a large and expensive instrument. An extension of the digital ELISA concept in [24] reduces the device cost ($2600) but at the price of losing sensitivity, and still requires hours of assay time by interleaving periods of pressurized reagent flow and wash steps. The significant work in [25] achieves a limit of 8 ng/liter TNFα using a surface-based sandwich assay format combined with the DNA-encoded antibody library (DEAL) technique. Moreover, the assay is multiplexed for 8-10 markers, with sample to answer in under 10 minutes. At the time of publication, the device readout used an expensive research-grade DNA microarray scanner. The nanopore approach could be the most cost and time efficient solution, provided the reagent and workflow optimization can achieve the needed detection sensitivity for this assay.

14 Detailed results with Tetanus Toxin (TT)

14.1 Methods

DNA-Antibody conjugate synthesis: 712bp DNA (biotin-712-thiol) was made in house at Two Pore Guys using proprietary template DNA and primers. The forward primer was modified to incorporate biotin on one end of the DNA and the reverse primer was modified to incorporate a thiol on the other. The thiol modified end of the DNA was utilized to attach it to an anti-Tetanus toxin IgG1 antibody (α-TTAb) utilizing SMCC/NHS-ester chemistry. The biotin modified end of the DNA was utilized to attach the DNA-antibody conjugate to Streptavidin coated magnetic beads. A restriction enzyme digest site was engineered at the biotin conjugated end of the DNA to allow for cleavage of the DNA-antibody conjugate from the streptavidin beads after Tetanus toxin (TT) (List Biological Laboratories, Inc. Cat #190B) binding in plasma. The DNA-Ab conjugate was prepared by first reacting the α-TTAb with sulfo-SMCC (sulfosuccinimidyl 4-(N-maleimidomethy)cyclohexane-1-carboxylate; Thermo Fisher, Cat #22322) in order to install a maleimide. Briefly, the α-TTAb was diluted to 100μM in 30μl of 10mM sodium phosphate pH 7.4 (NaPi) followed be the addition of 0.5μl of 70mM sulfo-SMCC (in DMSO) to achieve a 10:1 molar ratio of SMCC to α-TTAb. The reaction was incubated at room temperature for 1h. The α-TTAb-maleimide adduct was then purified using cation exchange liquid chromatography and concentrated to 50-100μM using a 30Kd molecular weight cutoff spin column. The purified α-TTAb-maleimide was then reacted with biotin-712-thiol. Biotin-712-thiol was diluted in 10mM NaPi, followed by the addition of α-TTAb-maleimide at a final concentration of 5-10μM (200:1 α-TTAb-maleimide to DNA). The reaction was incubated at room temperature for 1-2h.

Magnetic bead coating: Streptavidin coated magnetic beads (Invitrogen Cat # 65001) were washed 3X with magnetic bead wash buffer following the protocol provided with the beads. 5μg of the biotinylated DNA-antibody conjugate were incubated with 1mg of the washed beads overnight at 4°C to ensure complete conjugation to the beads. The beads were washed 3X with 100 μl NaPi, and divided up into the appropriate volume to yield aliquots containing 280ng of DNA-antibody conjugate bound to beads. The 280ng aliquots were then used for all TT nanopore experiments without and with spiked plasma.

Nanopore experiment sample preparation: TT studies with plasma were carried out using 20nM 712bp DNA or DNA-antibody conjugate (280ng) on bead with a final concentration of 600nM TT in NaPi and pH 7.4. Absent plasma, TT studies were carried out using 50nM DNA-antibody conjugate with a final concentration of 75 and 200nM TT. Control sample sets included 712bp DNA plus TT, DNA-antibody conjugate without TT and DNA-antibody conjugate with TT. Where stated, the control samples were spiked into 10 or 20% pooled human plasma. All samples were incubated at room temperature for 45 minutes. After incubation the samples were washed 1X with 100μL NaPi, removing plasma background. Following the wash, samples were cleaved off the bead by restriction enzyme digest (New England BioLabs Cat #R3193L) yielding DNA with a length of 594bp. Post cleavage, reagents were quality checked using EMSA (Figure S30a) to confirm full complex formation (10% polyacrylamide,120 mV in 1x TBE for 45 min). The cleaved samples were diluted into lithium chloride buffer for nanopore analysis at the final concentrations reported in the tables. The detection reagent in nanopore experiments with 594bp DNA is labeled DNA-α-TTAb in the tables. The false positive threshold was increased from 2% to 5%, to increase the margin of detection and reduce the time to positive detection.

To test an alternative to restriction enzyme digestion, a photocleavable linker was incorporated between the scaffold and the biotin, which can be cleaved by a timed exposure of UV light. Light-based liberation of detection reagent off beads is a simpler and less expensive workflow, amenable to integration into future test strip designs. As a test of this concept, UV light (365 nm) was exposed to the DNA-bead complexes for varying durations that matched restriction enzyme digestion times. PAGE analysis of the cleaved DNA by both methods (Figure S30b) suggests that the reactions are equally efficient, reaching completion in 15 seconds. Moreover, prolonged exposure (> 5 min) appears to degrade the DNA.

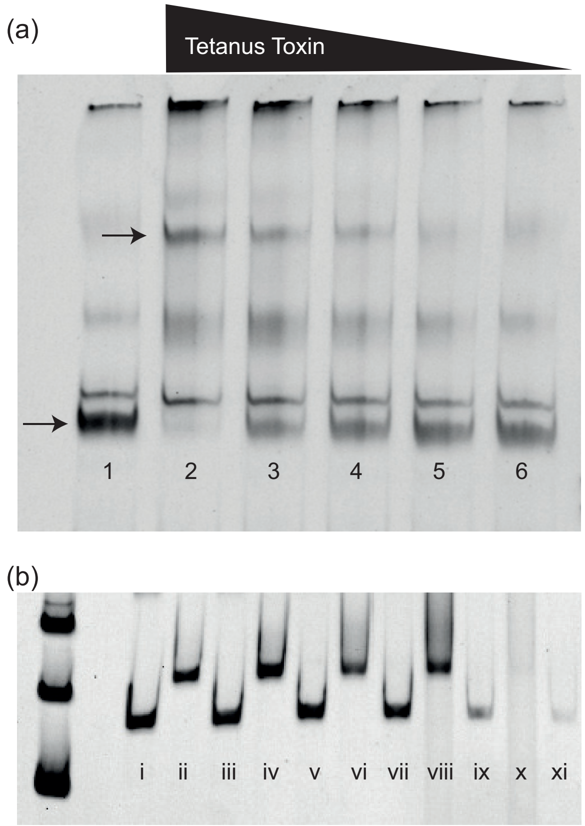

Figure S30: (a) EMSA showing dominant bands for DNA-α-TTAb alone (lane 1, arrow) and TT-bound DNA-α-TTAb (upper arrow) after incubation with varying TT concentrations (lane 2: 100 nM, 3: 75 nM, 4: 50 nM, 5: 25 nM, 6: 10 nM). (b) PAGE shows comparable performance for liberation of scaffold from streptavidin beads by restriction digest (RD) cleavage (594bp product) and by UV light cleavage of photocleavable linker (712bp product). Lanes adjacent to the ladder: i) positive RD control; ii) 15 sec UV; iii) 15 sec RD; iv) 30 sec UV; v) 30 sec RD; vi) 1 min UV; vii) 1 min RD; viii) 5 min UV; ix) 5 min RD; x) 20 min UV; xi) 20 min RD. The bands are offset due to the difference in liberated scaffold lengths.

14.2 Pores P32-P34: Detection

Experiments tested the DNA-α-TTAb detection reagent without and with TT, and a TT alone control, in triplicate using test strips and MOMs. The results are in Table S56. Positively detected TT concentrations were 200 nM and 75 nM at incubation and 12 nM and 4.5 nM, respectively, after dilution for nanopore recording. The TT alone produced a very low event rate.

Table S56: **Detection and time-to-positive data for Tetanus Toxin (TT)**

Pore ID, Diam.	Reagents & Concentrations*	Ratio† X	Tagged % ‡ (99% CI)	No. Events	Rec. Time	TTP 99%♮
P32	1. DNA-α-TTAb 3 nM	0	5 ± 1.7%	1059	10 min	np
80-82 nm	2. DNA-α-TTAb 3 nM + TT 12 nM	4	55.3 ± 4.1%	962	15 min	14 sec
Test strip	3. DNA-α-TTAb 3 nM + TT 4.5 nM	1.5	25.6 ± 2.6%	1882	9 min	5.9 sec
MOM	4. TT 12 nM	–	0 ± 0%	4	4 min	np
P33	1. DNA-α-TTAb 3 nM	0	3.93 ± 2.3%	458	9 min	np
78-83 nm	2. DNA-α-TTAb 3 nM	0	4.98 ± 2.2%	662	6 min	np
Test strip	3. DNA-α-TTAb 3 nM + TT 12 nM	4	40.4 ± 5.8%	470	11 min	2.7 sec
MOM	4. DNA-α-TTAb 3 nM + TT 4.5 nM	1.5	14.9 ± 3.5%	700	10 min	1.2 min
	5. TT 12 nM	–	16.7 ± 20%	24	5 min	np
P34	1. DNA-α-TTAb 3 nM	0	4.98 ± 1.6%	1245	5 min	np
88-91 nm	2. DNA-α-TTAb 3 nM + TT 12 nM	4	38.5 ± 3.3%	1461	5 min	5 sec
Test strip	3. DNA-α-TTAb 3 nM + TT 4.5 nM	1.5	11.5 ± 2.2%	1430	5 min	1.4 min
MOM	4. TT 12 nM	–	26.1 ± 24%	23	5 min	np

* Experiments were conducted at buffer pH 8.8, 1.5M LiCl, 10 mM Tris, 1 mM EDTA, and 100 mV. DNA = 594 bp. DNA-α-TTAb = DNA conjugated to the anti-TT antibody, 50 nM at incubation.

† X is the dimensionless target to scaffold/fusion concentration ratio, defined only when the complete detection reagent (scaffold/fusion) competent to bind target is present.

‡ Events are tagged positive if: max $\delta G > 1.78$ nS for P32; max $\delta G > 2.29$ nS for P33; max $\delta G > 3.2$ nS for P34. The reported % are $Q \pm Q_{\mathrm{conf}}$ as defined in Section 6.

♮ TTP (time-to-positive) is the first time at which the tagged percentage minus the 99% CI is greater than, and remains greater than, a false-positive threshold of 5%, as detailed in Section 6. The “np” entry for each reagent set reflects when this criteria is not satisfied.

Larger nanopores were used since both the TT target and the α-TTAb binding domain in the detection reagent are large molecules (150 kDa), resulting in a larger full complex than for any of the other targets studied. A representative scatter plot (P33) in Figure S31 shows that, despite the use of larger nanopores (80 nm diameter), the full complex events produce deep max δG values. The results support that the full complex produces a distinct max δG event signature that can be used for TT detection.

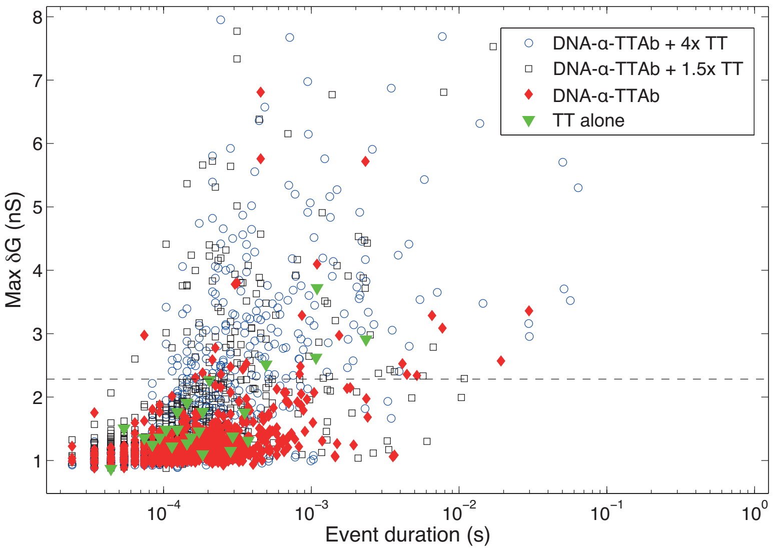

Figure S31: Pore P33 all-event scatter plot of max δG vs. duration for reagents 2-5. The horizontal line shows the max δG threshold above which events are tagged positive.

14.3 Pores P35-P36: Detection following on-bead incubation in plasma

Experiments in the presence of plasma tested the DNA-α-TTAb detection reagent without and with TT in duplicate, using test strips and MOMs. To test for specificity, DNA without the conjugated α-TTAb was tested in the presence of TT (absent plasma), and DNA-α-TTAb in the presence of plasma without TT was also measured. The results are reported in Table S57. Reagent sets incubated on bead in the presence of 10% or 20% plasma, prior to wash and cleavage off bead, are denoted by parentheses.

Table S57: **Detection and time-to-positive data for TT in plasma**

Pore ID, Diam.	Reagents & Concentrations*	Ratio† X	Tagged % ‡ (99% CI)	No. Events	Rec. Time	TTP 99%♮
P35	1. DNA-α-TTAb 4 nM	0	5.02 ± 1.9%	877	7 min	np
66-68 nm	2. DNA-α-TTAb 4 nM	0	4.69 ± 1.8%	896	7 min	np
Test strip	3. DNA 12 nM + TT 360 nM	–	1.66 ± 1.6%	421	10 min	np
MOM	4. DNA-α-TTAb 4 nM (10% plasma)	0	0.899 ± 1%	556	10 min	np
	5. DNA-α-TTAb 4 nM (20% plasma)	0	2.76 ± 1.7%	616	10 min	np
	6. DNA-α-TTAb 12 nM + TT 360 nM (10% plasma)	30	25 ± 4.1%	732	10 min	1.5 min
	7. DNA-α-TTAb 12 nM + TT 360 nM (20% plasma)	30	28.7 ± 4.4%	690	10 min	18 sec
P36	1. DNA-α-TTAb 4 nM	0	2.98 ± 2.3%	369	11 min	np
66-67 nm	2. DNA 12 nM + TT 360 nM	–	0.583 ± 1.1%	343	9 min	np
Test strip	3. DNA-α-TTAb 4 nM (10% plasma)	0	4.19 ± 2.4%	453	9 min	np
MOM	4. DNA-α-TTAb 4 nM (20% plasma)	0	4.9 ± 2.8%	388	9 min	np
	5. DNA-α-TTAb 12 nM + TT 360 nM (10% plasma)	30	33.2 ± 6.5%	346	10 min	29 sec
	6. DNA-α-TTAb 12 nM + TT 360 nM (20% plasma)	30	55.1 ± 7.7%	276	9 min	26 sec

* Experiments were conducted at buffer pH 8.8, 1.5M LiCl, 10 mM Tris, 1 mM EDTA, and 100 mV. DNA = 594 bp. DNA-α-TTAb = DNA conjugated to the anti-TT antibody.

† X is the dimensionless target to scaffold/fusion concentration ratio, defined only when the complete detection reagent (scaffold/fusion) competent to bind target is present.

‡ Events are tagged positive if: max $\delta G > 3.69$ nS for P35; max $\delta G > 2.9$ nS for P36; max $\delta G > 3.9$ nS for P31. The reported % are $Q \pm Q_{\text{conf}}$ as defined in Section 6.

♮ TTP (time-to-positive) is the first time at which the tagged percentage minus the 99% CI is greater than, and remains greater than, a false-positive threshold of 5%, as detailed in Section 6. The "np" entry for each reagent set reflects when this criteria is not satisfied.

A representative scatter plot (P35) is shown in Figure S32. Consistent with the results for buffer only, the presence of target to create full complex during incubation in plasma produces a marked increase in the fraction of events deep max δG values. The nanopore results also validate the specificity for the antibody binding domain for its target.

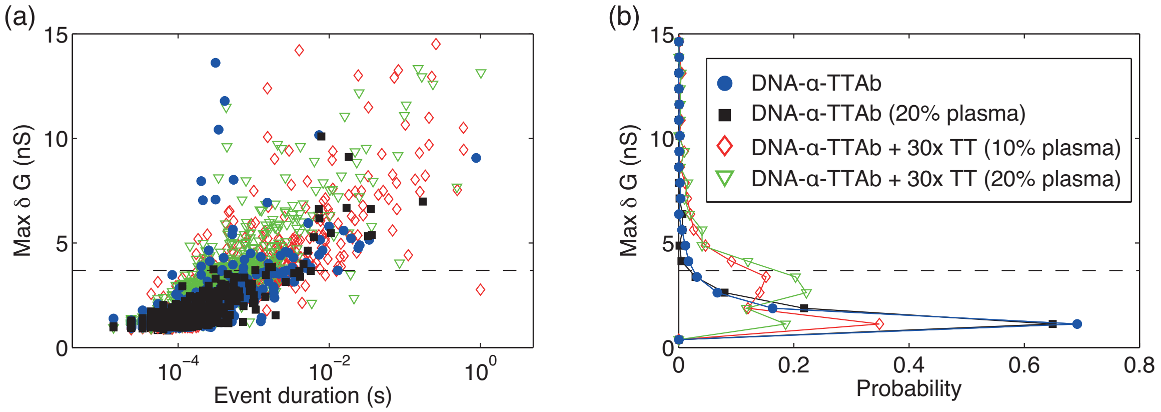

Figure S32: Pore P35 (a) all-event scatter plot of max δG vs. duration and (b) max δG histogram, for reagents 1 and 5-7. The horizontal line shows the max δG threshold above which events are tagged positive.

References

[1] S Boyd and L Vandenberghe. *Convex optimization.* Cambridge University Press, 2004.

[2] Trevor J Morin, Tyler Shropshire, Xu Liu, Kyle Briggs, Cindy Huynh, Vincent Tabard-Cossa, Hongyun Wang, and William B Dunbar. Nanopore-based target sequence detection. *PloS ONE*, 11(5):e0154426–21, May 2016.

[3] Min-Hyun Lee, Ashvani Kumar, Kyeong-Beom Park, Seong-Yong Cho, Hyun-Mi Kim, Min-Cheol Lim, Young-Rok Kim, and Ki-Bum Kim. A Low-Noise Solid-State Nanopore Platform Based on a Highly Insulating Substrate. *Scientific Reports*, 4:7448–7, December 2014.

[4] D R Garalde, C R O'Donnell, R D Maitra, D M Wiberg, Gang Wang, and William B Dunbar. Modeling the Biological Nanopore Instrument for Biomolecular State Estimation. *Control Systems Technology, IEEE Transactions on*, 21(6):2038–2051, 2013.

[5] Jacob K Rosenstein, Meni Wanunu, Christopher A Merchant, Marija Drndić, and Kenneth L Shepard. Integrated nanopore sensing platform with sub-microsecond temporal resolution. *Nature Methods*, 9(5):487–492, March 2012.

[6] Siddharth Shekar, David J Niedzwiecki, Chen-Chi Chien, Peijie Ong, Daniel A Fleischer, Jianxun Lin, Jacob K Rosenstein, Marija Drndić, and Kenneth L Shepard. Measurement of DNA Translocation Dynamics in a Solid-State Nanopore at 100 ns Temporal Resolution. *Nano Letters*, 16(7):4483–4489, July 2016.

[7] Joseph Larkin, Robert Y Henley, Murugappan Muthukumar, Jacob K Rosenstein, and Meni Wanunu. High-Bandwidth Protein Analysis Using Solid-State Nanopores. *Biophysj*, 106(3):696–704, February 2014.

[8] Mohammad Taherzadeh-Sani, Said M Hussain Hussaini, Hamidreza Rezaee-Dehsorkh, Frederic Nabki, and Mohamad Sawan. A 170-dBΩ CMOS TIA With 52-pA Input-Referred Noise and 1-MHz Bandwidth for Very Low Current Sensing. *IEEE Transactions on Very Large Scale Integration (VLSI) Systems*, pages 1–11, February 2017.

[9] Itaru Yanagi, Rena Akahori, Toshiyuki Hatano, and Ken-ichi Takeda. Fabricating nanopores with diameters of sub-1 nm to 3nm using multilevel pulse-voltage injection. *Scientific Reports*, 4:5000, May 2014.

[10] Eric Beamish, Harold Kwok, Vincent Tabard-Cossa, and Michel Godin. Precise control of the size and noise of solid-state nanopores using high electric fields. *Nanotechnology*, 23(40):405301–8, September 2012.

[11] J Kim, R Maitra, K D Pedrotti, and William B Dunbar. A Patch-Clamp ASIC for Nanopore-Based DNA Analysis. *IEEE Transactions on Biomedical Circuits and Systems*, 7(3):285–295, May 2013.

[12] Tom Fawcett. An introduction to ROC analysis. *Pattern Recognition Letters*, 27(8):861–874, June 2006.

[13] Richard Wyatt, Peter D Kwong, Elizabeth Desjardins, Raymond W Sweet, James Robinson, Wayne A Hendrickson, and Joseph G Sodroski. The antigenic structure of the HIV gp120 envelope glycoprotein. *Nature*, 393(6686):705–711, June 1998.

[14] Stefan W Kowalczyk, Alexander Y Grosberg, Yitzhak Rabin, and Cees Dekker. Modeling the conductance and DNA blockade of solid-state nanopores. *Nanotechnology*, 22(31):315101, July 2011.

[15] Meni Wanunu, Will Morrison, Yitzhak Rabin, Alexander Y Grosberg, and Amit Meller. Electrostatic focusing of unlabelled DNA into nanoscale pores using a salt gradient. *Nature Nanotechnology*, 5(2):160–165, February 2010.

[16] G F Reed, F Lynn, and B D Meade. Use of Coefficient of Variation in Assessing Variability of Quantitative Assays. *Clinical and Diagnostic Laboratory Immunology*, 9(6):1235–1239, November 2002.

[17] US Food Administration and Drug. *Guidance for industry, bioanalytical method validation.* https://www.fda.gov/downloads/Drugs/GuidanceComplianceRegulatoryInformation/Guidances/ucm070107.pdf, 2001.

[18] David A Armbruster and Terry Pry. Limit of blank, limit of detection and limit of quantitation. *The Clinical biochemist. Reviews / Australian Association of Clinical Biochemists*, 29 Suppl 1:S49–52, August 2008.

[19] Philip P Mortimer and John V Parry. Detection of antibody to HIV in saliva: a brief review. *Clinical and diagnostic virology*, 2(4-5):231–243, August 1994.

[20] R L Hodinka, T Nagashunmugam, and D Malamud. Detection of Human Immunodeficiency Virus Antibodies in Oral Fluids. *Clinical and Diagnostic Laboratory Immunology*, 5(4):419–426, July 1998.

[21] V Michalaki, K Syrigos, P Charles, and J Waxman. Serum levels of IL-6 and TNF-α correlate with clinicopathological features and patient survival in patients with prostate cancer. *British Journal of Cancer*, pages 1–5, May 2004.

[22] David M Rissin, Cheuk W Kan, Todd G Campbell, Stuart C Howes, David R Fournier, Linan Song, Tomasz Piech, Purvish P Patel, Lei Chang, Andrew J Rivnak, Evan P Ferrell, Jeffrey D Randall, Gail K Provuncher, David R Walt, and David C Duffy. Single-molecule enzyme-linked immunosorbent assay detects serum proteins at subfemtomolar concentrations. *Nature Biotechnology*, 28(6):595–599, May 2010.

[23] David M Rissin, Cheuk W Kan, Linan Song, Andrew J Rivnak, Matthew W Fishburn, Qichao Shao, Tomasz Piech, Evan P Ferrell, Raymond E Meyer, Todd G Campbell, David R Fournier, and David C Duffy. Multiplexed single molecule immunoassays. *Lab on a chip*, 13(15):2902–10, 2013.

[24] Francesco Piraino, Francesca Volpetti, Craig Watson, and Sebastian J Maerkl. A Digital-Analog Microfluidic Platform for Patient-Centric Multiplexed Biomarker Diagnostics of Ultralow Volume Samples. *ACS Nano*, 10(1):1699–1710, January 2016.

[25] Rong Fan, Ophir Vermesh, Alok Srivastava, Brian K H Yen, Lidong Qin, Habib Ahmad, Gabriel A Kwong, Chao-Chao Liu, Juliane Gould, Leroy Hood, and James R Heath. Integrated barcode chips for rapid, multiplexed analysis of proteins in microliter quantities of blood. *Nature Biotechnology*, 26(12):1373–1378, November 2008.
